# The macroeconomic burden of four alcohol-attributable diseases in China, 1990–2023, and projections to 2030: results from GBD 2023

**DOI:** 10.1080/07853890.2026.2741441

**Published:** 2026-10-16

**Authors:** Xue Yang, Limin Xin, Jianjin Yu, Qingyan Yang, Yanfeng Gong, Zhaoyu Guo, Kebing Yang

**Affiliations:** a Beijing Huilongguan Hospital, Capital medical university, Peking University HuiLongGuan Clinical Medical School, Beijing, China; b Precision and Intelligence Medical Imaging Lab, Beijing Friendship Hospital, Capital Medical University, Beijing, China; c Big Data Institute, Nuffield Department of Population Health, University of Oxford, UK Oxford

**Keywords:** Burden of disease, economic burden, alcohol-attributable diseases, value of lost welfare

## Abstract

**Background:**

Alcohol consumption contributes markedly to the global burden of disease, but macroeconomic assessments of alcohol-attributable diseases in China remain limited. Using Global Burden of Disease (GBD) data, this study quantified the macroeconomic burden of four alcohol-attributable diseases in China: alcohol use disorders (AUD), alcoholic cardiomyopathy (ACM), cirrhosis and other chronic liver diseases due to alcohol use (ALD), and liver cancer due to alcohol use (LCA).

**Materials and methods:**

Disability-adjusted life year (DALY) data for the four diseases from 1990 to 2023 were extracted from GBD 2023. The macroeconomic burden was estimated using the value of lost welfare (VLW) approach, which combined DALYs with age-adjusted value of a statistical life year (VSLY) estimates derived from willingness-to-pay methodology. Results are expressed in 2021 international dollars and as a percentage of GDP. Temporal trends were evaluated using Joinpoint regression analysis, and exploratory forecasts to 2030 were generated using ARIMA, XGBoost, and LightGBM models.

**Results:**

From 1990 to 2023, VLW increased substantially for all four diseases: AUD from $1.84 billion to $32.40 billion; ACM, $0.04 to $0.53 billion; ALD, $0.22 to $3.26 billion; and LCA, $0.09 to $2.48 billion. Joinpoint regression confirmed continuously increasing trends (all AAPC > 0, all *p* < 0.001). Males predominated across all conditions, with male-to-female VLW ratios of 7:1 (AUD), 10:1 (ALD), 3:1 (LCA), and 14:1 (ACM) in 2023. Age at peak VLW varied: 35–39 years for AUD, 50–59 years for ALD, 65–69 years for LCA, and 50–54 years for ACM (shifted from 35–39 years in 1990). Conditional on the continuation of their respective historical trends, the three models produced divergent exploratory projections to 2030.

**Conclusion:**

The macroeconomic burden of four alcohol-attributable diseases in China increased markedly from 1990 to 2023, with AUD consistently dominating and the burden overwhelmingly concentrated among males aged 35–64 years.

## KEY MESSAGES

- This is the first study to quantify the macroeconomic burden of four alcohol-attributable diseases (AUD, ACM, ALD, and LCA) in China from 1990 to 2023 with projections to 2030.
- The economic burden of each disease increased considerably over the 34-year period. AUD consistently accounted for a far higher VLW than the other three diseases, whereas LCA showed the most pronounced increase.
- This study suggests primary prevention (health education, reduced availability) for young adults aged 20–35 years, and secondary prevention for males aged 35–64 years: workplace-based AUD screening for ages 35–49 years and integrated hepatology-cardiology surveillance for ages 50–64 years.

## 1. Introduction

Alcohol consumption is a major contributor to disease burden, consistently ranking among the top ten risk factors in global comparative risk assessments [1–3]. In most regions, a small number of disease and injury categories, including alcohol use disorders (AUD), liver disease, cancer, cardiovascular disease, and injuries, account for over 90% of alcohol-attributable mortality and disability-adjusted life-years (DALYs) [4]. This study focuses on four alcohol-attributable diseases available from the Global Burden of Disease (GBD) study, including AUD, alcoholic cardiomyopathy (ACM), cirrhosis and other chronic liver diseases due to alcohol use (ALD), and liver cancer due to alcohol use (LCA). These four diseases represent the leading causes of alcohol-attributable mortality and disability in China [5,6].

AUD, characterized by compulsive heavy drinking and loss of control over intake, ranks among the most prevalent mental disorders globally [4,7]. The estimated global incidence of AUD among individuals aged 15–64 years in 2021 was 51.3 million [3]. The China Mental Health Survey (CMHS, 2013–2015) reported a 1.94% prevalence of alcohol and drug use disorders, ranking third among all mental disorders [8]. ALD, a major lethal consequence of alcohol use, ranges from simple steatosis to cirrhosis, with a global prevalence estimated at 3.5% [9,10]. In China, the age-standardized incidence rate of ALD was 3.59 per 100,000 population in 2019 [11]. Alcohol use is also a leading cause of hepatocellular carcinoma, accounting for 30.4% of cases globally [12]. In 2021, LCA contributed 99,544 incident cases and 2.3 million DALYs worldwide [13]. In China, liver cancer ranked fourth in incidence and second in mortality, with alcohol use as a major contributing factor [14]. ACM, an acquired dilated cardiomyopathy resulting from chronic excessive alcohol consumption, had a global prevalence of 368,691 cases and 1.47 million DALYs in 2021, representing increases of 31-48% over the preceding three decades [15]. In China, the prevalence of ACM in 2021 was 1.98 per 100,000 population [16]. The combined epidemiological burden of these four diseases underscores the need for macroeconomic impact assessment to inform equitable resource allocation and evidence-based policy formulation in China.

The value of lost welfare (VLW) approach is a standardized framework for estimating the economic losses attributable to disease burden [17]. The VLW model combines DALYs and the concept of value of statistical life (VSL), broadly defined as the value an individual is willing to pay (WTP) to decrease the risk of mortality [18]. The VLW method based on VSL estimates the economic burden of disease by quantifying the relationship between money and the risk of disability or death [19]. Compared to other estimation methods, this approach provides a more comprehensive reflection of the economic burden of disease, as it accounts for lost income, out-of-pocket spending related to medical care, the cost associated with pain and suffering, and the intrinsic value of life [20]. The VLW method is endorsed by the World Health Organization (WHO) for assessing the economic welfare impact of diseases [21].

Although VLW has been applied to quantify the macroeconomic burden of conditions such as congenital heart disease [22], musculoskeletal disorders [18], diabetes [23], and stroke [17], no study has systematically applied this framework to alcohol-attributable diseases in China. To address this gap, we used the VLW approach to estimate the macroeconomic burden of AUD, ACM, ALD, and LCA in China from 1990 to 2023, with exploratory projections to 2030. This integrated approach offers three contributions: (1) it monetizes the full welfare losses that conventional cost-of-illness studies may underestimate; (2) it enables direct comparison of relative burden across diseases and over time *via* GDP-standardized metrics; and (3) it generates forward-looking exploratory projections to inform policy prioritization.

## 2. Methods

### 2.1. Data collection and case definition

This study utilized data from the Global Burden of Diseases, Injuries, and Risk Factors Study 2023 (GBD 2023), accessed *via* the Global Health Data Exchange (GHDx) Results Tool (https://vizhub.healthdata.org/gbd-results/). The GBD 2023 study integrates evidence from over 160,000 sources, including vital registration, household surveys, disease registries, hospital records, and peer-reviewed literature. Its methodological robustness, particularly its handling of data heterogeneity and COVID-19 adjustments, ensures reliable estimates for policy-relevant research (see Supplementary Appendix S1.1 for details). For GBD studies, the Institutional Review Board of the University of Washington reviewed and approved a waiver of informed consent (https://www.healthdata.org/research-analysis/gbd). For this study, the DALYs for AUD, ACM, ALD, and LCA were extracted from the GBD 2023 for China. A detailed description of the ICD code classifications for these four diseases can be found in Supplementary Appendix S1.2. The detailed definitions for all metrics are provided in Supplementary Appendix S1.3. All procedures were conducted in accordance with the principles of the Helsinki Declaration.

### 2.2. VLW

#### 2.2.1. Annual projection of the VSL

To project VSL for each year *i* (${\text{VSL}}_{i}$), we applied an income elasticity (IE) model, scaling the baseline *VSL* by the adjusted gross domestic product (GDP) data of China in year *i* compared with the base year 2021:

$$\text{VS}L_{i}=\text{VS}L_{\text{base}}\;\times \;{\left(\frac{\text{GD}P_{i}}{\text{GD}P_{\text{base}}}\right)}^{\text{IE}}$$

The baseline *VSL* for China (${\text{VSL}}_{\text{base}}$ = 1,962,200 yuan) was derived from a systematic review of 28 empirical studies eliciting WTP from Chinese respondents across diverse risk contexts [24]. Its key determinants, including income, education, age, and risk perception, are incorporated through IE adjustment, age-adjustment functions, and sensitivity analyses, ensuring estimates reflect contemporary Chinese preferences and socioeconomic conditions [18,24]. Based on the 2021 average China-US exchange rate (approximately 1 yuan = $ 0.155) published by the Federal Reserve [25], ${\text{VSL}}_{\text{base}}$ is calculated to be $304,141. The purchasing power parity (PPP)-adjusted GDP data were obtained from the World Bank [26]. All results are presented in 2021 international dollars (PPP-adjusted). In this study, the term *IE* refers specifically to the income elasticity of the Value per Statistical Life (IE-VSL), defined as the percentage change in VSL associated with a 1% change in income. It measures how individuals’ WTP for reducing mortality risk changes in response to income growth [18].

#### 2.2.2. Calculation of VSLY

To account for the variation in the value of life across different ages, we applied an age-adjustment function f(a), which scales the *VSL* according to the proportion of life lived [18]:

$$\begin{array}{l} f\left(a\right)=\text{if}\frac{a}{\text{LE}}\leq 0.236;19.41{\left(0.236\right)}^{4}-43.170{\left(0.236\right)}^{3}+27.65{\left(0.236\right)}^{2}-4.33\left(0.236\right)+0.44;\text{if}\frac{a}{\text{LE}}\\ >0.236;19.41{\left(\frac{a}{LE_{i}}\right)}^{4}-43.170{\left(\frac{a}{LE_{i}}\right)}^{3}+27.65{\left(\frac{a}{LE_{i}}\right)}^{2}-4.33\left(\frac{a}{LE_{i}}\right)+0.44 \end{array}$$

where a denotes age (using midpoints of age groups) and ${\text{LE}}_{i}$ represents life expectancy at birth in China for year *i*. The life expectancy data were obtained from the World Bank.

The age-specific value of a statistical life-year (${\text{VSLY}}_{a}$) was then computed as:

$$\text{VSL}Y_{a}=\text{VS}L_{i}\;\times \;f\left(a\right)\;\times \;\frac{-r}{e^{-r\times L_{\text{Remaining},a}}-1}$$

where r is the discount rate, set at 3% per annum in alignment with standard health economic practice [23], and $L_{\text{Remaining},a}$ denotes the average remaining life expectancy at age a in China, sourced from United Nations Population Prospects [27].

#### 2.2.3. Aggregation of VLW

The total macroeconomic burden attributable to the four alcohol‑attributable diseases was calculated by summing the product of age-specific DALYs and the corresponding VSLY across all age groups:

$$\text{VLW}=\overset{\text{max}}{\underset{a=0}{\sum }}(\text{VSL}Y_{a}\times \;\text{DAL}Y_{a})$$

Furthermore, the VLW was expressed as a percentage of China’s annual GDP. This VLW-to-GDP ratio reflects the relative economic weight of the disease burden within the national economy over time, controlling for changes in economic scale and enabling consistent intertemporal comparison. As VLW captures welfare-based valuation, including pain, suffering, and the intrinsic value of life, rather than direct medical costs or productivity losses alone, this ratio should not be interpreted as a measure of forgone national output, but rather as a standardized metric for comparing relative welfare burden across diseases and over time [18].

Sensitivity analyses tested the robustness of our results to key assumptions, including alternative income elasticity values (IE = 0.55, 1.5) beyond our base case of 1.0. This range captures varying assumptions regarding the relationship between income and WTP for mortality risk reduction, in line with practices for extrapolating VSL estimates across income settings [18]. The VLW uncertainty intervals were derived by applying age-specific VSLY values to the lower and upper bounds of GBD DALY estimates, following standard VLW practice [18,23]. Uncertainty from VSL, income elasticity, life expectancy, and discount rate assumptions was not incorporated into the main UIs but was evaluated through sensitivity analyses, consistent with prior VLW studies [18,23]. The study adhered to the Consolidated Health Economic Evaluation Reporting Standards (CHEERS) 2022 guidelines.

### 2.3. ARIMA

The autoregressive integrated moving average (ARIMA) model is a well-established statistical framework for analyzing and forecasting time-series data [28]. As a classical time-series forecasting method, the ARIMA (p, d, q) model is particularly applicable to stationary time series or those that can be rendered stationary through differencing [29]. Given its extensive application as a conventional statistical framework within the GBD analytical system, ARIMA was employed in this study to assess the predictive performance of established statistical methodologies in forecasting VLW trends. The ARIMA model can be expressed as:

$$y_{t}=c+\varphi_{1}y_{t-1}+\varphi_{2}y_{t-2}+\ldots +\varphi_{p}y_{t-p}+a_{t}-\theta_{1}a_{t-1}-\theta_{2}a_{t-2}-\ldots -\theta_{q}a_{t-q}$$

where c denotes a constant term, while $y_{t}$ represents the observed value of the time series at time *t*. The parameter *p* indicates the order of the autoregressive (AR) component, with $\varphi_{1}$, $\varphi_{2}$, …, $\varphi_{p}$ corresponding to the respective AR coefficients. Similarly, q denotes the order of the moving average (MA) component, with $\theta_{1}$, $\theta_{2}$, …, $\theta_{q}$ representing the MA coefficients. A foundational assumption of the model is that the random error term $a_{t}$ follows a white noise process, that is, independently and identically distributed with a mean of zero and a constant variance *σ*^2^.

### 2.4. XGBoost

XGBoost is an efficient, flexible, and scalable gradient-boosting decision tree algorithm [30]. It uses parallel tree boosting techniques, which are known for their ability to solve data science problems quickly and effectively [31]. Both L1 (lasso) and L2 (ridge) regularization terms are integrated into XGBoost, enabling it to effectively alleviate overfitting risks. This regularization framework enhances the model’s generalizability for future mortality predictions while improving forecasting accuracy and stability [29]. The mathematical formulation of XGBoost is as follows:

$$\hat{y_{i}}=\sum_{j=1}^{K}\left(f_{j})(X_{i}\right)$$

where $\hat{y_{i}}\;$ is the predicted value for the i-th sample. $y_{i}$ is the corresponding true observed value. *K* is the number of decision trees, and $\left(f_{j})(X_{i}\right)$ represents the predicted value from the *j*-th decision tree for the *i*-th sample.

### 2.5. LightGBM

LightGBM, a recent improvement of the gradient boosting algorithm, inherited its high predictivity but resolved its scalability issues and long computational time by adopting a leaf-wise tree growth strategy and introducing novel techniques [32]. LightGBM is a lightweight gradient boosting algorithm similar to XGBoost. LightGBM calculates the histogram of a node by performing histogram subtraction on its sibling nodes and parent nodes, which allows the node histograms to be reused (only one node needs to be built for each split), resulting in additional performance improvements [33]. The mathematical formulation was:

$${ℶ}^{\left(t\right)}=\sum_{i=1}^{n}l(y_{i},{\hat{y}}_{i}^{\left(t-1\right)}+f_{t}(x_{i}))+\epsilon (f_{t})$$

where n denotes the number of training samples, $x_{i}\;$ represents the feature vector of the *i*-th sample, $y_{i}\;$ corresponds to the true response (target value) of the *i*-th sample, ${\hat{y}}_{i}^{\left(t-1\right)}$ denotes the predicted value of the same sample obtained from the previous *(t-1)*-th iteration, $f_{t}\;$ represents the regression tree added at iteration *t*, which maps the input feature vector $x_{i}$ to a corresponding leaf weight, l is a differentiable loss function that quantifies the discrepancy between the true value $y_{i}\;$ and the predicted value, and ϵ denotes the regularization term, which is introduced to control model complexity and reduce the risk of overfitting [34].

### 2.6. Statistical analysis

As shown in Figure 1, following the calculation of VLW, Joinpoint regression analysis was employed to monitor and evaluate the temporal trends in the VLW from 1990 to 2023. This technique identifies changes in data trends over time and fits the most appropriate model linking different line segments on a logarithmic scale [35]. The average annual percentage change (AAPC) was calculated for the entire period (1990–2023), and the annual percentage change (APC) was calculated for each segment by the model. The hypothesis test assessed whether the AAPC or APC was significantly different from zero; AAPC or APC > 0 means an increasing trend, and AAPC or APC < 0 means decreasing trend [35]. The analysis was performed with Joinpoint Regression Program software (version 5.4.0.0).

**Figure 1.**
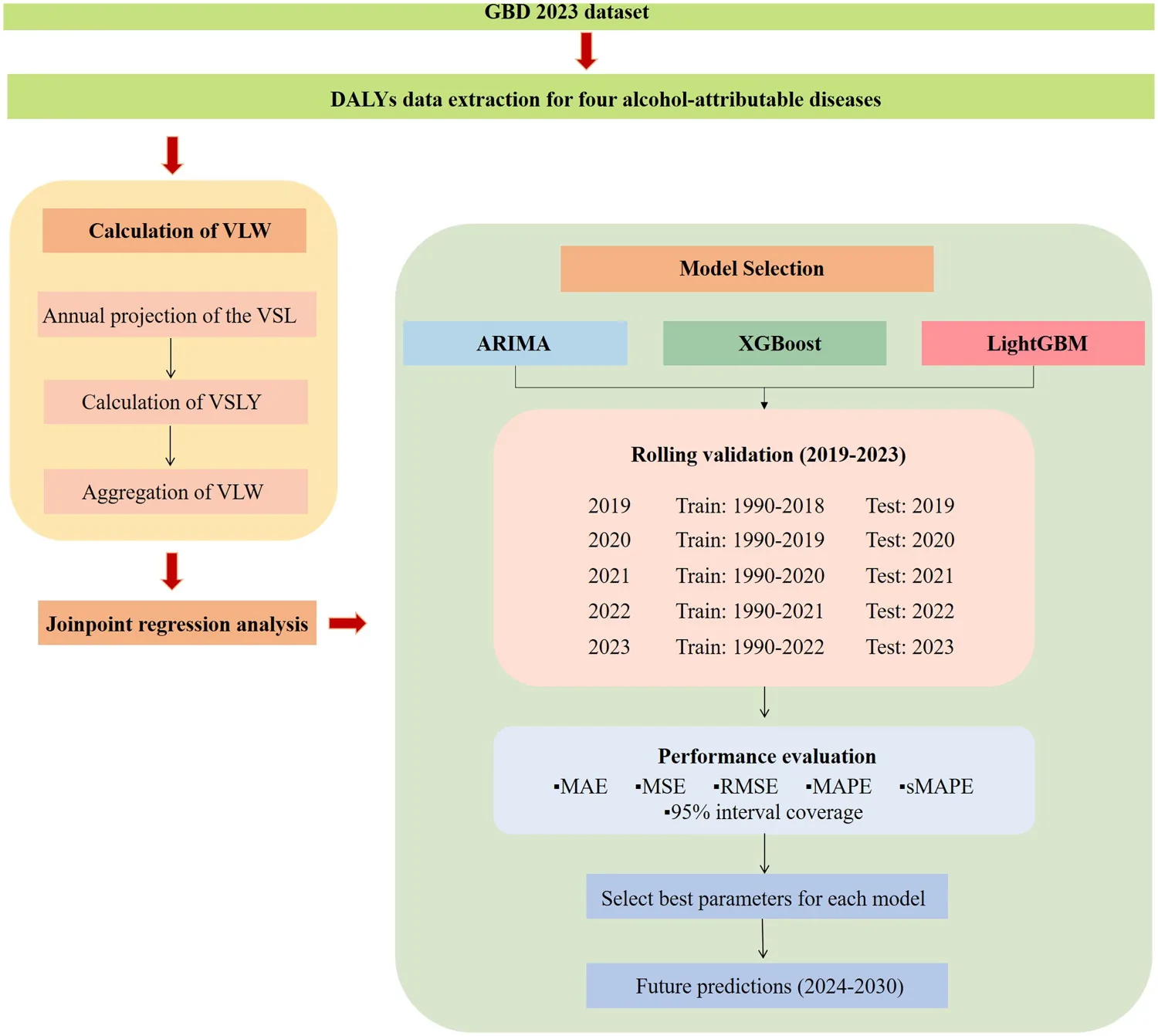
Study design and analytical framework.

Then, three machine learning models, including ARIMA, XGBoost, and LightGBM, were employed for exploratory forecasting. For ARIMA, optimal orders (p, d, q) were automatically selected by minimizing the corrected Akaike Information Criterion (AICc) using the auto.arima() function. Model adequacy was assessed *via* the Ljung-Box Q test and residual ACF test. For XGBoost and LightGBM, a comprehensive feature engineering strategy captured temporal dynamics, including scaled time indices, polynomial trends, seasonal components, lagged variables, first-order differences, growth rates, and rolling averages. Model development followed a rolling validation framework (2019–2023) with expanding training windows. Hyperparameters were optimized by minimizing RMSE using adaptive hybrid search (XGBoost) and Bayesian optimization (LightGBM). Uncertainty intervals were constructed using model-based bootstrap or residual-based approaches; detailed implementation procedures for both models are provided in Supplementary Appendix S4. The decision to present an alternative ALD forecasting model was made post hoc based on the forecast results. Because the AICc-preferred ARIMA(0,2,0) model produced negative lower uncertainty bounds in 2029–2030, ARIMA(0,1,1) was presented as a nonnegative alternative, while ARIMA(0,2,0) was retained as a sensitivity analysis.

Model comparisons were primarily conducted using the Diebold–Mariano (DM) test. To characterize the magnitude and nature of forecast performance, descriptive metrics, including MAE, MSE, RMSE, MAPE, sMAPE, and 95% UI coverage, were additionally reported for each model. After validation, the final models were retrained on the full dataset (1990–2023) with optimal parameters to generate out-of-sample forecasts for 2024–2030, along with 95% UI to quantify uncertainty. The full executable code is provided in Supplementary Materials 2, along with the raw and derived datasets and all calculation parameters. These analyses were conducted using R (version 4.5.2).

## 3. Results

### 3.1. Macroeconomic burden of four alcohol‑attributable diseases

From 1990 to 2023, the economic burden attributable to the four alcohol‑attributable diseases exhibited an increasing trend. In 1990, the VLW for AUD, ACM, ALD, and LCA was $1.840 billion, $0.041 billion, $0.217 billion, and $0.088 billion, respectively; by 2023, these values had increased to $32.395 billion, $0.529 billion, $3.262 billion, and $2.483 billion, respectively. AUD consistently accounted for the predominant burden across all time points, with its VLW far exceeding that of the other three diseases and this disparity widened substantially over time. However, when expressed as a share of GDP, the economic burden of these four diseases remained relatively stable over the 34-year period. AUD had the highest GDP share (0.097% in 1990 and 0.101% in 2023), followed by ALD (0.012% and 0.010%), LCA (0.005% and 0.008%), and ACM (0.002% in both years) (these percentages indicate relative burden and do not represent actual GDP losses). Further details are presented in Table 1.

**Table 1.** Value of lost welfare (VLW) and VLW/gross domestic product (GDP) attributable to four alcohol‑attributable diseases in China, 1990–2023.

year	AUD		ACM		ALD		LCA	
	VLW ($ billion)	VLW/GDP (%)	VLW ($ billion)	VLW/GDP (%)	VLW ($ billion)	VLW/GDP (%)	VLW ($ billion)	VLW/GDP (%)
1990	1.840(1.263,2.605)	0.097(0.067,0.138)	0.041(0.012,0.134)	0.002(0.001,0.007)	0.217(0.130,0.355)	0.012(0.007,0.019)	0.088(0.059,0.127)	0.005(0.003,0.007)
1991	2.083(1.427,2.955)	0.101(0.069,0.143)	0.046(0.014,0.147)	0.002(0.001,0.007)	0.233(0.142,0.372)	0.011(0.007,0.018)	0.097(0.065,0.140)	0.005(0.003,0.007)
1992	2.445(1.660,3.463)	0.103(0.070,0.146)	0.052(0.016,0.170)	0.002(0.001,0.007)	0.262(0.162,0.407)	0.011(0.007,0.017)	0.112(0.075,0.160)	0.005(0.003,0.007)
1993	2.840(1.925,4.020)	0.105(0.071,0.149)	0.059(0.018,0.194)	0.002(0.001,0.007)	0.295(0.183,0.449)	0.011(0.007,0.017)	0.130(0.087,0.185)	0.005(0.003,0.007)
1994	3.262(2.214,4.617)	0.107(0.073,0.152)	0.066(0.020,0.221)	0.002(0.001,0.007)	0.331(0.210,0.492)	0.011(0.007,0.016)	0.151(0.102,0.212)	0.005(0.003,0.007)
1995	3.639(2.454,5.140)	0.108(0.073,0.152)	0.072(0.023,0.245)	0.002(0.001,0.007)	0.367(0.236,0.538)	0.011(0.007,0.016)	0.173(0.116,0.244)	0.005(0.003,0.007)
1996	3.983(2.701,5.603)	0.107(0.073,0.151)	0.078(0.024,0.260)	0.002(0.001,0.007)	0.404(0.262,0.592)	0.011(0.007,0.016)	0.194(0.131,0.277)	0.005(0.004,0.008)
1997	4.255(2.905,5.951)	0.105(0.071,0.146)	0.083(0.027,0.274)	0.002(0.001,0.007)	0.443(0.289,0.648)	0.011(0.007,0.016)	0.213(0.144,0.300)	0.005(0.004,0.007)
1998	4.469(3.125,6.221)	0.102(0.071,0.142)	0.087(0.029,0.290)	0.002(0.001,0.007)	0.484(0.315,0.698)	0.011(0.007,0.016)	0.229(0.156,0.320)	0.005(0.004,0.007)
1999	4.690(3.325,6.510)	0.099(0.070,0.138)	0.091(0.030,0.308)	0.002(0.001,0.007)	0.530(0.351,0.776)	0.011(0.007,0.016)	0.247(0.170,0.348)	0.005(0.004,0.007)
2000	5.044(3.589,6.986)	0.098(0.070,0.136)	0.097(0.033,0.321)	0.002(0.001,0.006)	0.586(0.392,0.842)	0.011(0.008,0.016)	0.271(0.188,0.379)	0.005(0.004,0.007)
2001	5.497(3.962,7.561)	0.099(0.071,0.136)	0.101(0.036,0.335)	0.002(0.001,0.006)	0.637(0.433,0.909)	0.012(0.008,0.016)	0.299(0.206,0.419)	0.005(0.004,0.008)
2002	6.063(4.386,8.286)	0.100(0.072,0.136)	0.108(0.038,0.349)	0.002(0.001,0.006)	0.701(0.487,0.998)	0.012(0.008,0.016)	0.324(0.222,0.452)	0.005(0.004,0.007)
2003	6.798(4.952,9.203)	0.102(0.074,0.138)	0.120(0.045,0.369)	0.002(0.001,0.006)	0.781(0.555,1.089)	0.012(0.008,0.016)	0.351(0.240,0.489)	0.005(0.004,0.007)
2004	7.596(5.604,10.243)	0.103(0.076,0.139)	0.131(0.051,0.390)	0.002(0.001,0.005)	0.877(0.634,1.206)	0.012(0.009,0.016)	0.381(0.262,0.520)	0.005(0.004,0.007)
2005	8.552(6.314,11.422)	0.104(0.077,0.139)	0.151(0.064,0.436)	0.002(0.001,0.005)	0.973(0.709,1.324)	0.012(0.009,0.016)	0.433(0.299,0.598)	0.005(0.004,0.007)
2006	9.670(7.157,12.877)	0.105(0.077,0.139)	0.183(0.081,0.487)	0.002(0.001,0.005)	1.078(0.784,1.437)	0.012(0.009,0.016)	0.510(0.353,0.712)	0.006(0.004,0.008)
2007	11.021(8.128,14.731)	0.104(0.077,0.140)	0.224(0.097,0.527)	0.002(0.001,0.005)	1.205(0.880,1.606)	0.011(0.008,0.015)	0.618(0.428,0.869)	0.006(0.004,0.008)
2008	12.019(8.888,16.051)	0.104(0.077,0.139)	0.261(0.105,0.565)	0.002(0.001,0.005)	1.289(0.951,1.714)	0.011(0.008,0.015)	0.714(0.491,1.007)	0.006(0.004,0.009)
2009	13.085(9.646,17.510)	0.103(0.076,0.138)	0.296(0.112,0.608)	0.002(0.001,0.005)	1.365(1.009,1.792)	0.011(0.008,0.014)	0.802(0.550,1.127)	0.006(0.004,0.009)
2010	14.350(10.598,19.128)	0.102(0.076,0.137)	0.333(0.120,0.663)	0.002(0.001,0.005)	1.458(1.079,1.898)	0.010(0.008,0.014)	0.890(0.609,1.256)	0.006(0.004,0.009)
2011	15.622(11.378,20.989)	0.102(0.074,0.137)	0.368(0.125,0.715)	0.002(0.001,0.005)	1.581(1.160,2.081)	0.010(0.008,0.014)	0.964(0.659,1.356)	0.006(0.004,0.009)
2012	16.919(12.174,22.719)	0.102(0.074,0.137)	0.394(0.128,0.755)	0.002(0.001,0.005)	1.768(1.285,2.345)	0.011(0.008,0.014)	1.063(0.726,1.508)	0.006(0.004,0.009)
2013	18.305(13.071,24.770)	0.103(0.073,0.139)	0.419(0.130,0.786)	0.002(0.001,0.004)	1.943(1.414,2.578)	0.011(0.008,0.015)	1.175(0.800,1.670)	0.007(0.005,0.009)
2014	19.894(14.175,27.019)	0.104(0.074,0.141)	0.439(0.133,0.811)	0.002(0.001,0.004)	2.149(1.569,2.840)	0.011(0.008,0.015)	1.339(0.915,1.900)	0.007(0.005,0.010)
2015	21.577(15.408,29.406)	0.105(0.075,0.143)	0.459(0.137,0.840)	0.002(0.001,0.004)	2.333(1.700,3.109)	0.011(0.008,0.015)	1.511(1.037,2.131)	0.007(0.005,0.010)
2016	23.398(16.737,32.028)	0.107(0.077,0.146)	0.483(0.146,0.889)	0.002(0.001,0.004)	2.506(1.826,3.390)	0.011(0.008,0.016)	1.689(1.156,2.387)	0.008(0.005,0.011)
2017	25.332(18.069,34.837)	0.108(0.077,0.149)	0.495(0.154,0.917)	0.002(0.001,0.004)	2.649(1.914,3.594)	0.011(0.008,0.015)	1.807(1.228,2.592)	0.008(0.005,0.011)
2018	27.107(19.304,37.234)	0.109(0.077,0.149)	0.484(0.156,0.897)	0.002(0.001,0.004)	2.818(2.022,3.823)	0.011(0.008,0.015)	1.853(1.260,2.660)	0.007(0.005,0.011)
2019	28.635(20.136,39.532)	0.108(0.076,0.149)	0.465(0.154,0.896)	0.002(0.001,0.003)	2.962(2.144,3.981)	0.011(0.008,0.015)	1.831(1.255,2.586)	0.007(0.005,0.010)
2020	29.106(20.375,40.171)	0.107(0.075,0.148)	0.445(0.149,0.886)	0.002(0.001,0.003)	3.023(2.206,4.053)	0.011(0.008,0.015)	1.787(1.244,2.524)	0.007(0.005,0.009)
2021	31.845(22.481,43.887)	0.108(0.076,0.149)	0.496(0.166,1.006)	0.002(0.001,0.003)	3.332(2.383,4.478)	0.011(0.008,0.015)	2.096(1.446,2.981)	0.007(0.005,0.010)
2022	33.120(23.249,45.851)	0.109(0.077,0.151)	0.537(0.172,1.089)	0.002(0.001,0.004)	3.516(2.314,5.074)	0.012(0.008,0.017)	2.395(1.584,3.561)	0.008(0.005,0.012)
2023	32.395(22.721,44.707)	0.101(0.071,0.140)	0.529(0.170,1.114)	0.002(0.001,0.004)	3.262(2.139,4.806)	0.010(0.007,0.015)	2.483(1.594,3.808)	0.008(0.005,0.012)

Abbreviations: AUD, Alcohol use disorders; ACM, Alcoholic cardiomyopathy; ALD, Alcohol-induced liver cirrhosis and other chronic liver diseases; LCA, Alcohol-induced liver cancer; VLW, value of lost welfare; GDP, gross domestic product.

Sex-stratified analysis (Figure 2) revealed that the VLW for all four diseases increased more rapidly in males than in females from 1990 to 2023. This was particularly pronounced for AUD, for which the VLW in males rose from $1.536 billion to $27.757 billion over the period, compared with an increase from $0.280 billion to $3.989 billion in females. In 2023, the male-to-female VLW ratios were approximately 7:1 for AUD, 3:1 for LCA, 10:1 for ALD, and 14:1 for ACM.

**Figure 2.**
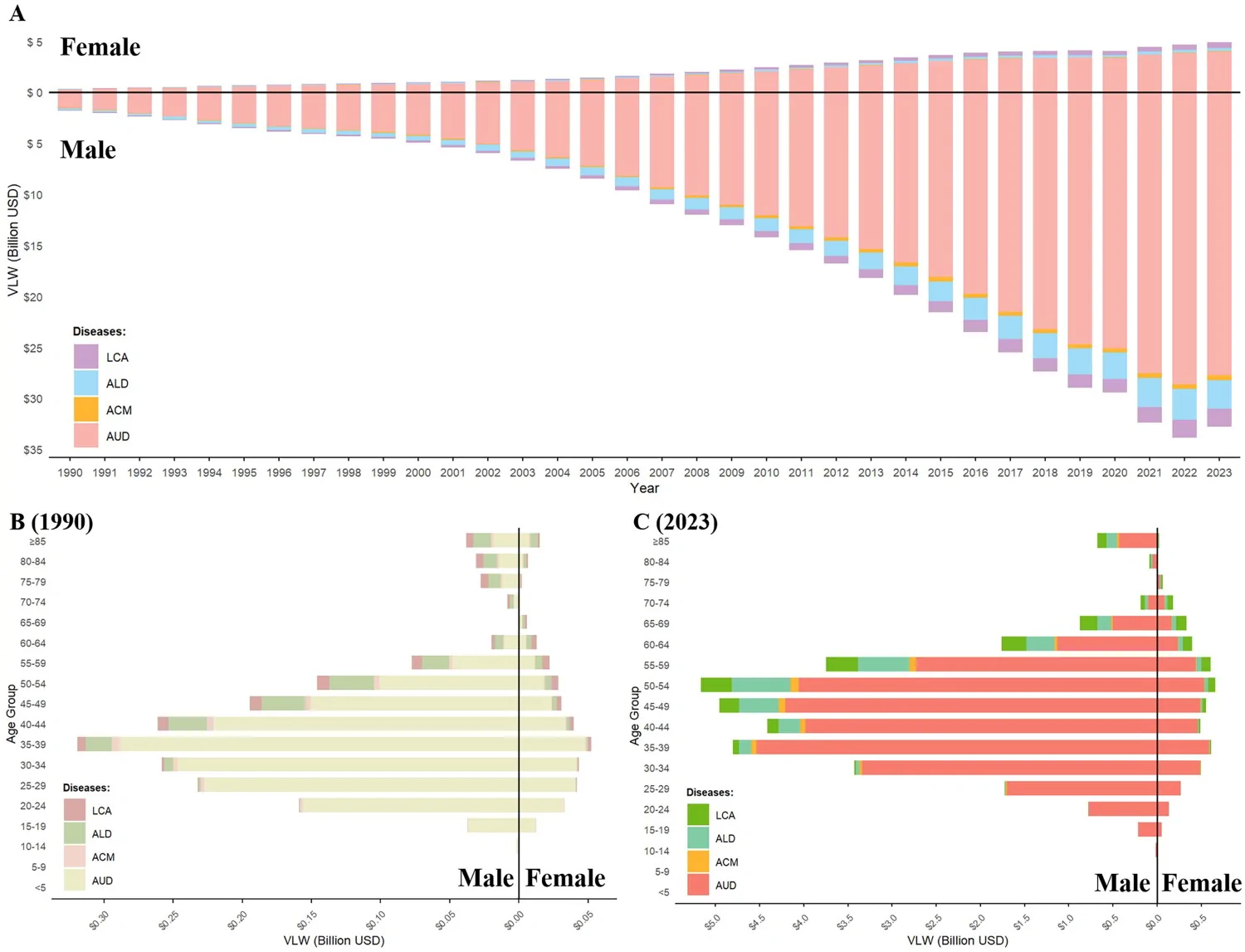
Economic burden of four alcohol‑attributable diseases in China, 1990–2023. (A: Annual VLW stratified by year, sex, and cause, 1990–2023; B: VLW by sex and cause in 1990; C: VLW by sex and cause in 2023. Abbreviations: VLW, value of lost welfare; AUD, Alcohol use disorders; ACM, Alcoholic cardiomyopathy; ALD, Alcohol-induced liver cirrhosis and other chronic liver diseases; LCA, Alcohol-induced liver cancer.

Analysis by sex and age group in 1990 and 2023 showed that VLW was higher in males than in females across all age groups, with the exception of the 65–69 age group in 1990 and the 75–79 age group in 2023. By disease causes, AUD burden peaked in males aged 35–39 years in both 1990 and 2023. ACM burden peaked in males aged 35–39 years in 1990 but shifted to 50–54 years in 2023. ALD burden was highest in males aged 50–54 years in both 1990 and 2023, and also in the 55–59 age group in 2023. LCA burden peaked in the 60–64 age group in 1990 and in the 65–69 age group in 2023. Sex-stratified VLW and VLW/GDP results are presented in Supplementary Table S1.1. Sensitivity analyses using alternative IE values (0.55 and 1.5) yielded VLW and VLW/GDP estimates broadly consistent with the main analysis, confirming the robustness of our findings (Supplementary Tables S1.2–3).

### 3.2. Joinpoint regression analysis

Joinpoint regression analysis confirmed increasing trends for all four alcohol-attributable diseases in China (all AAPC > 0, all *p* < 0.001) (Figure 3). Among these, LCA exhibited the most pronounced increase (AAPC = 10.852), followed by AUD (AAPC = 9.130), ALD (AAPC = 8.612), and ACM (AAPC = 8.157). Notably, LCA’s growth accelerated over time, whereas the upward trends for AUD and ALD showed gradual deceleration in recent years. ACM displayed a fluctuating pattern, with a temporary decline during 2016–2020 followed by resumed growth. Sex-stratified analyses revealed largely consistent patterns, except that LCA maintained a persistently increasing trend in males throughout the study period. Detailed APC estimates for each segment are presented in Supplementary Table S1.4.

**Figure 3.**
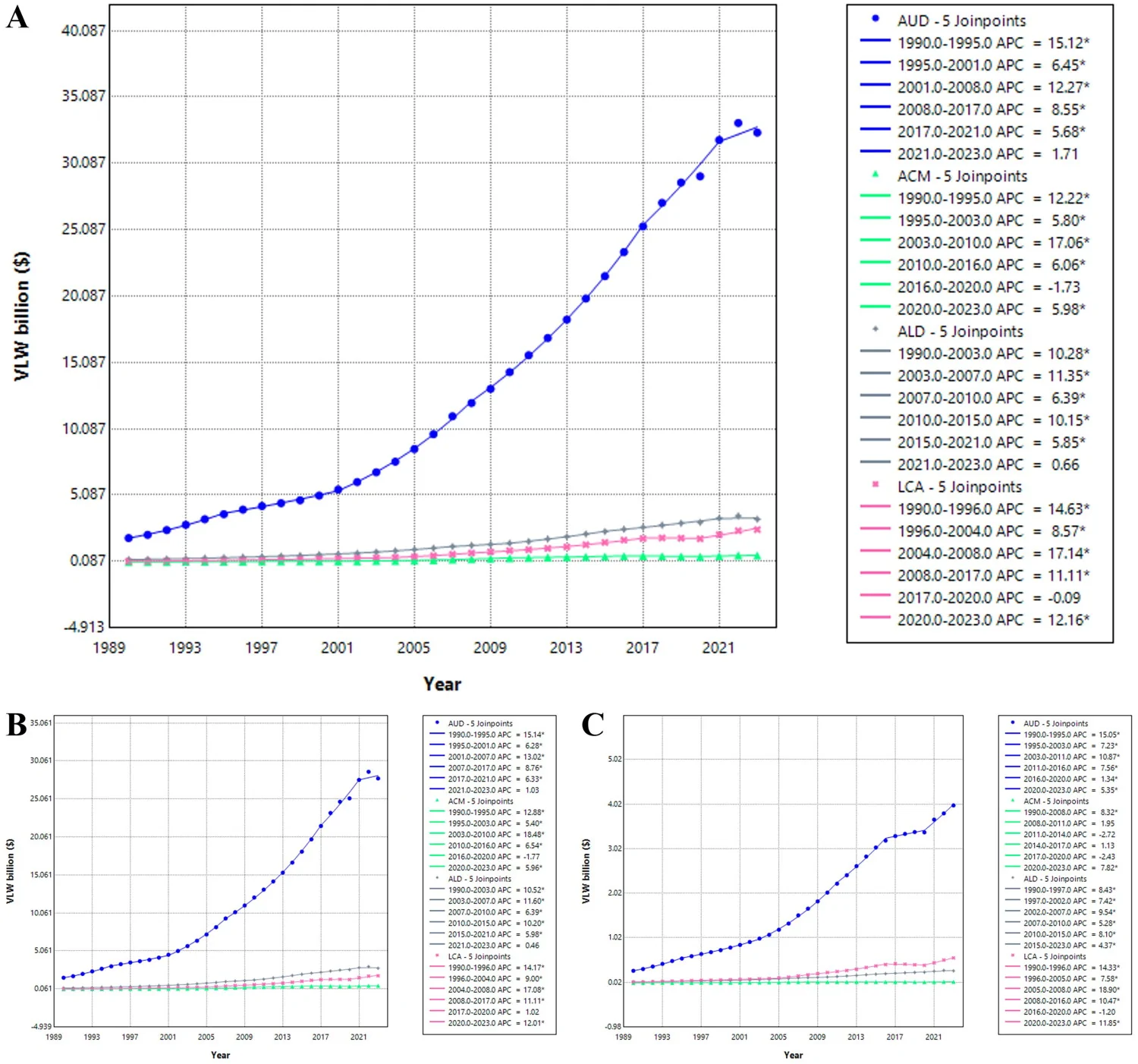
Joinpoint regression analysis of VLW for four alcohol‑attributable diseases stratified by sex in China, 1990–2023. (A: Total; B: Male; C: Female. Abbreviations: VLW, value of lost welfare; AUD, Alcohol use disorders; ACM, Alcoholic cardiomyopathy; ALD, Alcohol-induced liver cirrhosis and other chronic liver diseases; LCA, Alcohol-induced liver cancer; AAPC: average annual percentage change; APC: annual percentage change). Notable trend shifts include LCA’s accelerated growth over time, gradual deceleration of AUD and ALD in recent years, and a temporary decline in ACM during 2016–2020 followed by resumed growth. All diseases exhibited significant increasing trends (all AAPC > 0, all *p* < 0.001).

### 3.3. Forecasts for burden of alcohol‑attributable diseases

All three modelling approaches, LightGBM, ARIMA and XGBoost, are exploratory in nature and should be interpreted as providing reference-only projections, contingent upon the continuation of the historical trends and underlying assumptions embedded in each model. In exploratory pairwise DM tests (Supplementary Table S1.6), LightGBM significantly outperformed XGBoost across all 12 disease-sex series. ARIMA significantly outperformed XGBoost in 10 of 12 series, while none of the ARIMA-versus-LightGBM comparisons was statistically significant.

The three models did not converge on a uniform direction of change. LightGBM projected a modest increase for AUD and ACM but a slight decline for ALD and LCA; ARIMA predicted sustained increases across all four conditions; and XGBoost estimated modest declines for each disease. This divergence underscores the inherent uncertainty in long-term forecasts and cautions against overinterpretation of any single model’s results. ARIMA,XGBoost, and LightGBM exploratory projections are presented in Supplementary Table S1.7– S1.9, respectively.

Notably, for ALD, the preferred ARIMA(0,2,0) model violated the admissibility criterion by producing negative lower uncertainty bounds; consequently, the highest-ranked admissible model, ARIMA(0,1,1), was selected as the primary model, with projections from the preferred model retained as a sensitivity analysis (Supplementary Tables S1.5 and S1.7). Sex-specific analyses revealed consistent patterns, with males contributing the majority of the economic burden across all diseases. The trends in VLW from 1990 to 2030 as estimated by the three models are displayed in Figure 4.

**Figure 4.**
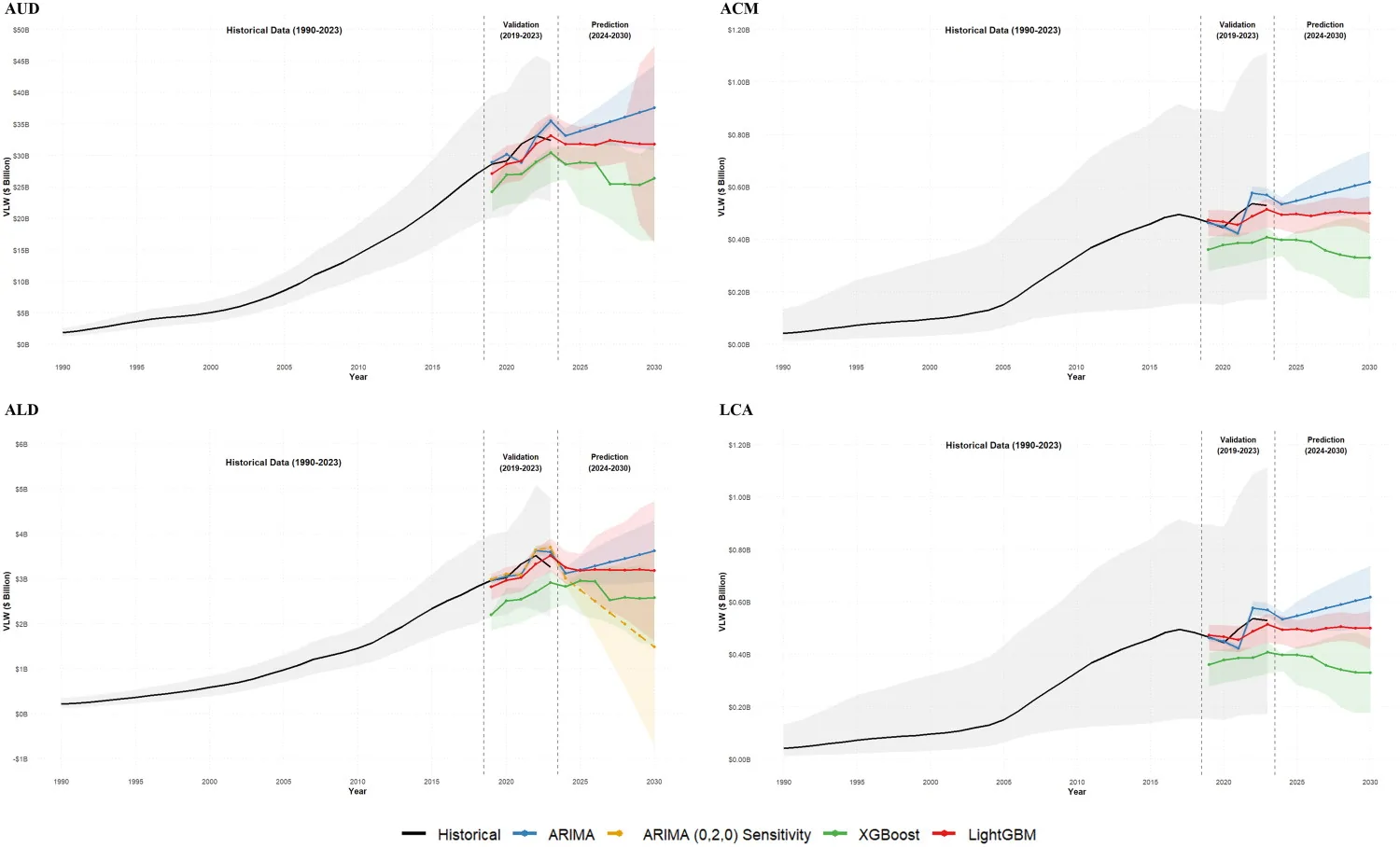
Temporal trends in the economic burden of four alcohol-attributable diseases in China, 1990–2030. Historical data (1990–2019) are shown alongside rolling validation predictions (2019–2023) and forecasted values (2024–2030) from ARIMA, XGBoost, and LightGBM models. Shaded areas represent 95% uncertainty intervals. Abbreviations: VLW, value of lost welfare; AUD, Alcohol use disorders; ACM, Alcoholic cardiomyopathy; ALD, Alcohol-induced liver cirrhosis and other chronic liver diseases; LCA, Alcohol-induced liver cancer.

## 4. Discussion

This study provides the first comprehensive macroeconomic assessment of four alcohol-attributable diseases in China from 1990 to 2023, with exploratory projections extending to 2030. AUD consistently accounted for a higher VLW than the other three diseases, whereas LCA exhibited the most pronounced increase over the study period. The dominance of AUD reflects its larger DALY burden and younger age distribution, which yield higher VSLY values under the VLW framework. In contrast, ALD, LCA, and ACM predominantly affect older populations. The relative stability of VLW/GDP ratios from 1990 to 2023 suggests that the epidemiological burden has not outpaced economic growth. Thus, a substantial portion of the absolute VLW increase appears to be driven by GDP-linked VSL growth rather than worsening population health alone, highlighting that increases in VLW primarily reflect welfare valuation and economic growth, whereas the underlying epidemiological burden of disease differs fundamentally in that it is measured by DALYs, incidence, or mortality independent of economic conditions.

The divergent projections across LightGBM, XGBoost and ARIMA likely reflect differences in algorithmic structure, sensitivity to historical data, and handling of temporal dependencies. This divergence is not an isolated finding. In the context of infectious disease forecasting, multimodel ensemble studies have similarly demonstrated that individual models often produce variable projections in magnitude and uncertainty, even when they agree on the relative ranking of interventions [36]. These projections should therefore be interpreted as exploratory scenarios conditional on the continuation of observed trends, not as definitive predictions.

Given that AUD consistently contributes the largest VLW burden, screening initiatives should be explicitly linked to evidence-based treatment pathways. However, U.S. data have shown persistently low pharmacotherapy uptake despite increasing AUD admissions, underscoring a treatment-delivery gap that warrants attention [37]. This highlights the need for integrated screening-to-treatment strategies to translate early detection into effective burden reduction in China.

The most striking finding across all four diseases was the extreme and persistent sex disparity. In 2023, the male-to-female VLW ratios were approximately 7:1 for AUD, 10:1 for ALD, 3:1 for LCA, and 14:1 for ACM. This disparity is consistent with the CMHS showing that the prevalence of AUD among Chinese males was over ten times higher than among females [38]. This gap reflects a combination of biological, behavioral, and sociocultural factors. Alcohol consumption remains deeply embedded in Chinese social culture, particularly at traditional festivals, weddings, and business gatherings. A large cross-sectional study across 10 Chinese provinces reported that mean weekly alcohol consumption among men ranged from 195 g to 422 g, and the prevalence of weekly drinking ranged from 7% to 51% [39]. The prevalence of heavy episodic drinking in China was much higher than in many other middle-income countries [40]. These findings underscore that Chinese males bear the predominant burden of these four diseases, highlighting the urgent need for targeted interventions prioritizing this high-risk population.

Superimposed on this male predominance, distinct age-specific burden patterns further refine the priorities. Age-stratified analysis showed that the highest VLW for AUD occurred in males aged 35–39 years, the peak working-age population, highlighting the potential of workplace-based screening and brief interventions as a targeted strategy for early detection [41]. ALD burden peaked at ages 50–54 and 55–59 years, reflecting the 10- to 20-year latency between sustained heavy drinking and the development of advanced liver fibrosis or cirrhosis [42]. LCA burden peaked at 65–69 years, consistent with the multi-stage carcinogenesis process in which cumulative alcohol exposure leads to hepatocellular carcinoma after decades of chronic liver injury [43]. ACM burden shifted from the 35–39 age group in 1990 to the 50–54 age group in 2023, suggesting an age shift toward older age groups, consistent with the increasing burden observed in older populations in recent years [16].

Taken together, these findings indicate that the cumulative burden of the four diseases is concentrated among males spanning from early midlife (35–39 years, driven by AUD) through late midlife (50–64 years, driven by ALD and ACM) to early older age (65–69 years, driven by LCA). However, given the 10- to 40-year latency between sustained heavy drinking and disease onset, primary prevention should target males as early as young adulthood (ages 20–35 years) to prevent the establishment of hazardous drinking patterns. For secondary prevention, interventions targeting males aged 35–64 years, a demographic encompassing the peak burden periods of AUD, ALD, and ACM, should be prioritized in national alcohol control strategies. Within this broad age range, differentiated approaches are warranted: workplace-based screening and brief interventions for AUD are most relevant for those aged 35–49 years, whereas integrated hepatology and cardiology surveillance programs for ALD, LCA, and ACM are particularly critical for those aged 50–64 years. A systematic review reported that only 20.8% of patients with alcohol-attributable hepatocellular carcinoma received regular surveillance, compared with 35.0% for hepatitis B virus-associated cases, highlighting a critical gap in clinical practice that must be addressed [12].

The “Healthy China 2030” strategic plan explicitly calls for strengthening alcohol control health education, reducing excessive alcohol use, and decreasing alcohol-attributable harm [44]. The WHO Global Alcohol Action Plan 2022–2030 recommends a suite of evidence-based “best buys” for alcohol policy, including: (1) increasing excise taxes on alcoholic beverages; (2) enacting and enforcing comprehensive restrictions on alcohol advertising, sponsorship, and promotion; (3) enacting and enforcing restrictions on the physical availability of alcohol (e.g. reduced hours of sale, minimum legal purchase age); and (4) implementing drink-driving countermeasures, including random breath testing, and lowering blood alcohol concentration limits [45]. Unlike drink-driving enforcement, which has achieved notable success since 2011, China’s alcohol taxation, marketing restrictions, and availability controls remain substantially underdeveloped relative to WHO recommendations [40]. Compared with other countries, China’s excise taxes on alcoholic beverages remain relatively low, and import duties and tariffs on alcohol have been progressively reduced, with tariff exemptions extended to alcoholic products from multiple countries. In addition, digital advertising has emerged as a new form of alcohol marketing that currently remains unregulated. Strengthening alcohol taxation and marketing regulation therefore warrants serious consideration as part of a comprehensive national alcohol control strategy aligned with the Healthy China 2030 goals.

This paper still has several limitations. First, the GBD 2023 data rely on modeling assumptions and may underestimate the true burden of the four alcohol-attributable diseases in China due to underdiagnosis in primary care settings and incomplete vital registration in some regions. Second, the VLW approach, based on WTP methodology, may undervalue lower-income populations’ health. Moreover, VLW estimates are sensitive to key parametric assumptions, including VSL, income elasticity, and discount rate. Although sensitivity analyses confirmed robust VLW/GDP ratios, absolute dollar values varied across scenarios. Thus, VLW figures should be interpreted as order-of-magnitude estimates for comparative purposes rather than precise point estimates of actual losses. Third, the forecasting models did not account for potential major policy interventions (e.g. sudden implementation of a national alcohol tax increase or advertising ban) or unexpected social behavior shifts (e.g. post-pandemic changes in drinking patterns). Fourth, given the limited validation period, neither empirical coverage-based recalibration nor explicit multi-step uncertainty accumulation could be implemented for either model. The intervals reported are therefore model-based uncertainty intervals and should be interpreted with caution. Furthermore, the DM test based on only five forecast errors yields inherently imprecise inference. Fifth, this study was limited to four alcohol-attributable diseases and did not include other alcohol-attributable conditions, such as pancreatitis, tuberculosis, and injuries, which represent additional economic burden not captured in the present estimates, as disaggregated data specific to these conditions were not directly available from the GBD database. Sixth, the ICD code classifications for the four diseases may have limited sensitivity for detecting early-stage alcohol-related organ damage that does not yet meet diagnostic coding thresholds. Seventh, as a forecasting study based on historical trend extrapolation, our analysis does not establish causal relationships between specific policy interventions and changes in disease burden. Eighth, and critically, these disease-specific VLW estimates cannot be summed to produce a total alcohol-attributable burden due to potential comorbidity overlap. Future studies should employ individual-level multi-morbidity adjustment or population attributable fraction methods to address this limitation.

## 5. Conclusion

In summary, this study quantifies, for the first time, the macroeconomic burden of four alcohol-attributable diseases in China using the VLW framework, as measured by welfare-based VLW rather than direct health outcomes alone. From 1990 to 2023, VLW rose substantially across all four diseases, with AUD consistently contributing the largest burden. The stable VLW/GDP ratios suggest that much of this absolute growth likely reflects economic-driven valuation growth. Exploratory projections to 2030 diverged across three models, underscoring forecast uncertainty and the need for cautious interpretation. This study underscores the need for prioritized, age-stratified interventions targeting males. Primary prevention, including school and community-based health education and reduced alcohol availability, should target males as early as young adulthood (ages 20–35 years) to prevent the establishment of hazardous drinking patterns. Secondary prevention should focus on males aged 35–64 years, encompassing workplace-based AUD screening and brief interventions for those aged 35–49 years, and integrated hepatology-cardiology surveillance for ALD, LCA, and ACM for those aged 50–64 years. This evidence base supports policy prioritization and resource allocation for alcohol control in China.

## Supplementary Material

Supplementary Materials2_VLW calculation all analysis datasets and the codes of the three prediction models.xlsx

Supplementary Materials 1_202060924_clean.docx

## Data Availability

The data were obtained through an online query tool from the website of IHME (https://ghdx.healthdata.org/), and no permissions were required to access the data.
