# Supplementary material for: The macroeconomic burden of four alcohol-attributable diseases in China, 1990–2023, and projections to 2030: results from GBD 2023
Source: Ann Med. 2026 Oct 16;58(1):2741441. doi: 10.1080/07853890.2026.2741441 (PMC13678882; doi:10.1080/07853890.2026.2741441)
Supplement: Supplementary file 1 [file IANN_A_2741441_SM7793.docx]

# *Contents*

| **Appendix 1.** Data sources, processing workflow, and methodological enhancements in GBD 2023 | -2- |
| --- | --- |
| **Appendix 2.** List of International Classification of Diseases (ICD) codes mapped to alcohol related diseases in GBD 2021 (Latest version) | -6- |
| **Appendix 3.** Metric definitions of this study | -7- |
| Appendix 4. Statistical analysis of XGBoost and LightGBM | -9- |
| **Table 1.** The VLW and VLW/GDP for alcohol-attributable diseases in China between 1990 and 2023 based on GBD 2023 | -11- |
| **Table 2.** Sensitivity analysis of VLW and VLW/GDP estimates for alcohol-attributable diseases in China, 1990–2023, based on GBD 2023 under the assumption of IE = 0.55 | -20- |
| **Table 3.** Sensitivity analysis of VLW and VLW/GDP estimates for alcohol-attributable diseases in China, 1990–2023, based on GBD 2023 under the assumption of IE = 1.5 | -22- |
| Table 4. Joinpoint regression analysis of VLW from 1990 to 2023 for alcohol-attributable diseases in China | -24- |
| **Table 5.** Forecasting accuracy of three models for predicting VLW (Billion USD) using a 5-year rolling window approach | -28- |
| Table 6. Comparative performance of forecasting models across diseases and sexes with Diebold-Mariano test | -36- |
| **Table 7.** ARIMA model-based projections of VLW (Billion USD) for alcohol-attributable diseases in China, 2024–2030 | -37- |
| **Table 8.** XGBoost model-based projections of VLW (Billion USD) for alcohol-attributable diseases in China, 2024–2030 | -39- |
| **Table 9.** LightGBM model-based projections of VLW (Billion USD) for alcohol-attributable diseases in China, 2024–2030 | -41- |

## Appendix 1. Data sources, processing workflow, and methodological enhancements in GBD 2023

In Global Burden of Disease (GBD) 2023, the data processing workflow is a crucial step to ensure the accuracy and consistency of global disease burden estimates. This process includes systematic adjustments to epidemiological data to correct for biases arising from differences in data sources, definitions, and measurement methods. These adjustments are implemented using complex statistical models such as Meta-Regression—Bayesian, Regularized, Trimmed (MR-BRT) and Disease Modeling—Meta-Regression (DisMod-MR 2.1), ensuring internal consistency in estimates of disability-adjusted life years (DALYs) across different regions, ages, genders, and years. This process aims to minimize heterogeneity in the study results through standardization and correction steps.

**1. Identification and extraction of data**

The GBD 2023 builds upon over 160,000 data sources, integrating vital registration, survey data (e.g., Demographic and Health Surveys, DHS), hospital records, and disease registries to estimate both fatal and nonfatal burdens worldwide. To ensure cross-national comparability, raw data are harmonized using advanced statistical tools. For fatal burden estimation, models like the Cause of Death Ensemble model (CODEm) and Spatio-Temporal Gaussian Process Regression (ST-GPR) are refined to account for disruptions such as the COVID-19 pandemic. For nonfatal conditions, DisMod-MR 2.1 and network meta-analyses synthesize evidence across diverse settings. This comprehensive, model-driven approach generates consistent estimates of Disability-Adjusted Life Years (DALYs) for every location, even where direct data are sparse.

**2. Data processing and heterogeneity control**

The GBD 2023 fundamentally retains the advanced Meta-Regression, Bayesian, Regularized, Trimmed (MR-BRT) framework from GBD 2021 for standardizing heterogeneous epidemiological data by estimating correction factors from paired observations. The primary evolution lies in its significantly expanded scope and enhanced sophistication. MR-BRT models are now applied more extensively to a wider array of diseases and risk factors, systematically adjusting for a broader spectrum of biases including measurement error and sampling bias. A key methodological advancement is the increased use of ensemble modeling and model averaging, which integrates results from multiple model specifications to produce more robust estimates and, crucially, to more fully propagate model selection uncertainty into the final confidence intervals. Furthermore, the entire data processing pipeline has achieved greater automation and integration, ensuring more consistent and reproducible harmonization across the vast GBD cause list. These refinements strengthen the comparability and validity of estimates as the study incorporates an ever-growing and more diverse global data universe.

**3. Epidemiological estimation**

Building upon the robust framework of GBD 2021, the GBD 2023 study introduces several key methodological refinements across its estimation processes while maintaining core analytical engines. For epidemiological estimation and years lived with disability (YLD) calculation, the study continues to rely primarily on DisMod-MR 2.1 and Spatio-Temporal Gaussian Process Regression (ST-GPR). A notable advancement is the enhanced modeling of sequela distributions. Rather than relying predominantly on fixed proportions from sources like the Medical Expenditure Panel Survey (MEPS), GBD 2023 employs more dynamic, disease-specific models to estimate the proportion of cases falling into each severity level (from asymptomatic to severe), allowing for greater adaptability to varying epidemiological contexts across locations and over time. The multiplication of sequela-specific prevalence by corresponding disability weights remains the foundation for crude YLD estimation.

For comorbidity adjustment, GBD 2023 retains the simulation-based approach but integrates it more seamlessly with the updated sequela models. It continues to simulate a large number of hypothetical individuals (e.g., 20,000 per demographic group) to estimate the coexistence of multiple nonfatal conditions. The method for calculating cumulative disability weight for an individual with multiple sequelae has been refined, ensuring the additivity of YLDs within the cause hierarchy with greater computational efficiency and theoretical consistency.

In the estimation of YLLs and DALYs, GBD 2023 upholds the principle of calculating Years of Life Lost (YLLs) by multiplying deaths by a standard life expectancy at age of death. The Cause of Death Ensemble model (CODEm) remains the primary tool for mortality estimation for most causes. However, the algorithms for redistributing deaths assigned to "garbage codes" (non-specific or implausible causes) have been updated and refined using the latest available data on multiple causes of death and expert review, improving the accuracy of underlying cause attribution. The process of summing YLLs and YLDs to obtain DALYs continues, with uncertainty propagated through 500 draws. A significant development in GBD 2023 is the explicit modeling and reporting of the impact of the COVID-19 pandemic on cause-of-death data completeness and reporting patterns, which required specialized adjustments to maintain time-series consistency for many causes.

Finally, uncertainty and heterogeneity analysis in GBD 2023 remains rigorous. The study continues to report 95% uncertainty intervals (UIs) derived from 500 simulation draws, comprehensively capturing uncertainty from data input, model selection, and estimation processes. The use of advanced bias-adjustment tools, such as Meta-Regression—Bayesian, Regularized, Trimmed (MR-BRT), is even more extensive to address data heterogeneity arising from differing case definitions, measurement methods, and source types. This ensures that observed trends in disease burden estimates more accurately reflect true epidemiological changes rather than artifacts of data variability.

**4. Data release and application**

The GBD 2023 findings, building upon the GBD 2021 framework, have been fully integrated into the Global Health Data Exchange (GHDx). This includes the release of new data visualization tools and updated results for over 160,000 sources. The primary goal remains to provide an evidence-based, comprehensive framework for tracking disease burden and risk factors, directly supporting global and national health policy and resource allocation.

Through this continuously refined process, GBD 2023 reinforces the scientific rigor and global comparability of disease burden estimates by implementing more extensive and sophisticated standardization techniques. The study provides an updated, reliable foundation for global health policy and planning by further controlling for heterogeneity through advanced bias-adjustment models and by explicitly accounting for major disruptions such as the COVID-19 pandemic in its uncertainty analysis. The enhanced methodological precision, including dynamic sequela modeling and refined mortality redistribution algorithms, reflects the ongoing commitment to high standards necessary for accurately understanding and addressing evolving global health challenges.

**5. Data extraction**

We extracted data on alcohol-related disease burden for China from the GBD 2023 using the GBD Results Tool (<https://vizhub.healthdata.org/gbd-results/>). The extraction parameters were configured as follows: Measure – Disability-Adjusted Life Years (DALYs); Causes – all causes with "alcohol" in the disease name, encompassing alcohol use disorders and other alcohol-attributable conditions; Location – China; Age – all age groups from under 5 years, then 5–9, 10–14, 15–19, 20–24, … 80–84, and 85 years and older; Sex – both sexes combined, male, and female separately; Years – 1990 to 2023; Metric – all available options including Number, Percent, and Rate. We then clicked the download button to obtain the complete raw dataset. We have deposited the original GBD 2023 downloaded files, all derived analytic datasets generated during the VLW calculation process, and the complete analysis code for VLW computation in **Supplementary Materials 2**, along with all relevant parameters used in the calculations, to ensure full transparency and reproducibility.

## Appendix 2. List of International Classification of Diseases (ICD) codes mapped to alcohol related diseases in GBD 2021 (Latest version)

| **Cause Name** | **ICD10** | **ICD10 Used in Hospital/Claims Analyses** | **ICD9** | **ICD9 Used in Hospital/Claims Analyses** |
| --- | --- | --- | --- | --- |
| Alcohol use disorders | E24.4, F10-F10.99, G31.2, G62.1, R78.0, X65-X65.9, Y15-Y15.9 | F10.2-F10.8, G31.2, X65-X65.9, Y15-Y15.9 | 291-291.9, 303-303.93, 305-305.03, 790.3, E860-E860.19, V11.3 | 291.0-291.9, 303.0-303.93 |
| Alcoholic cardiomyopathy | I42.6 | _ | 425.5 | _ |
| Cirrhosis and other chronic liver diseases due to alcohol use | The absence of direct ICD codes for "alcohol-attributable cirrhosis" and "alcohol-attributable liver cancer" is because the ICD system classifies diseases by their diagnostic manifestation (e.g., cirrhosis, liver cancer), while the GBD study reclassifies and attributes these cases to specific underlying causes (e.g., alcohol use) through statistical estimation. | | | |
| Liver cancer due to alcohol use |  |  |  |  |

## Appendix 3. Metric definitions of this study

| **Measure** | **D****efinition** | **Number** | **Percent** | | **Rate/Criteria** |
| --- | --- | --- | --- | --- | --- |
| Disability adjusted life years (DALYs) | The sum of years lost due to premature death (YLLs) and years lived with disability (YLDs). One DALY equals one lost year of healthy life. | Number of DALYs in the population | Proportion of DALYs for a particular cause relative to DALYs for all causes | | DALYs per 100,000 population |
| Mean Absolute Error (MAE) | A measure of average error magnitude without considering direction. | |   *n* = number of observations  $\hat{y}$= predicted value  *y_i_* = actual value | | |
| Mean Squared Error (MSE) | The Mean Squared Error measures the average squared difference between predicted values and actual observed values, providing a metric that heavily penalizes larger errors. | | $MSE=\frac{1}{n}\sum_{i=1}^{n} {(\hat{y}_{i}-y_{i})}^{2}$  *n* = number of observations  $\hat{y}$= predicted value  *y_i_* = actual value | | |
| Absolute Percentage Error (APE) | The percentage deviation of individual predictions from actual values. | |   $\hat{y}_{i}$= predicted value  *y_i_* = actual value | | |
| Root Mean Square Error (RMSE) | A quadratic scoring rule that measures the average magnitude of error, giving higher weight to larger errors. | |   *n* = number of observations  $\hat{y}_{i}$= predicted value  *y_i_* = actual value | | |
| Mean Absolute Percentage Error (MAPE) | The average of absolute percentage errors across all predictions. |   *n* = number of observations  $\hat{y}_{i}$= predicted value  *y_i_* = actual value | | MAPE < 2%: Excellent MAPE 2-5%: Good MAPE 5-10%: Acceptable MAPE > 10%: Poor | |
| Symmetric Mean Absolute Percentage Error (sMAPE) | The sMAPE is an improved version of the traditional MAPE that addresses its limitations by incorporating a symmetric treatment, thereby avoiding bias when predicted or actual values approach zero. | $sMAPE=\frac{100\%}{n}\sum_{i=1}^{n} \frac{2\vert\hat{y}_{i}-y_{i}\vert}{\left\vert\hat{y}_{i} \right\vert+\vert y_{i}\vert}$  *n* = number of observations  $\hat{y}_{i}$= predicted value  *y_i_* = actual value  $\left\vert\hat{y}_{i} \right\vert+ \vert y_{i}\vert$= sum of absolute predicted and actual values (symmetric treatment) | | sMAPE < 2%: Excellent sMAPE 2-5%: Good sMAPE 5-10%: Acceptable sMAPE 10-15%:  Fair  sMAPE≥15%:  Poor | |
| 95% Prediction Interval Coverage (95% Interval Coverage) | The proportion of actual values that fall within the 95% prediction intervals. | Coverage ≥ 95%: Excellent Coverage 90-95%: Good Coverage 80-90%: Acceptable Coverage < 80%: Poor | | | |
| 95% uncertainty intervals (UI) | To estimate the 95% UIs for the predictions, 500 bootstrap samples were generated, with models trained on each sample. The 95% UI was defined using the 2.5th and 97.5th percentiles of the predicted outcomes. | | | | |

## Appendix 4. Statistical analysis of XGBoost and LightGBM

**1. XGBoost**

For XGBoost, a comprehensive feature engineering strategy was implemented to capture temporal dynamics, including time index, scaled year, polynomial trends (linear, quadratic, cubic), seasonal components (periods 5, 7, and 10), lagged variables (1-3 years), first‑order differences, growth rates, and rolling averages (3‑ and 5‑year). Hyperparameters were optimized using an adaptive hybrid search procedure that combined global exploration, local fine‑tuning, and multi‑stage refinement, targeting RMSE minimization over the rolling validation period (2019-2023), with expanding training windows. The optimized hyperparameters included learning rate (eta), tree depth (max_depth), subsample fractions (subsample, colsample_bytree), regularization parameters (lambda, alpha, gamma, min_child_weight), and the number of boosting rounds (nrounds). Uncertainty intervals (UIs) were constructed using a bootstrap procedure with 500 iterations: training residuals were resampled with replacement to generate bootstrap samples, the model was refit on each sample, and the 2.5th and 97.5th percentiles of the resulting forecast distribution were taken as the 95% UI. When the number of residuals was insufficient for bootstrap resampling (fewer than 5), a normal approximation based on the residual standard deviation was used as a fallback.

No empirical coverage‑based recalibration procedure was implemented in the final code, as the validation set (2019-2023) contained only five annual observations per disease‑sex series, which was insufficient to reliably estimate and apply a calibration factor. Consequently, the reported intervals are model‑based bootstrap UIs rather than empirically calibrated prediction intervals.

**2. LightGBM**

For LightGBM, the same feature engineering strategy was applied. Hyperparameters were optimized using Bayesian optimization to minimize RMSE over the rolling validation period (2019–2023), with training data defined as year < test_year (excluding the target year). The optimized parameters included learning rate (learning_rate), tree structure (num_leaves, max_depth, min_data_in_leaf), sampling fractions (feature_fraction, bagging_fraction), regularization (lambda_l1, lambda_l2, min_gain_to_split), and the number of boosting rounds (nrounds). UIs were derived from the standard deviation of training residuals using a normal approximation.

No bootstrap resampling was performed for interval estimation, and no empirical coverage‑based recalibration or explicit multi‑step uncertainty accumulation was implemented in the final code. As with XGBoost, the limited validation window (five annual forecasts) precluded reliable estimation of empirical coverage for calibration purposes. The intervals should therefore be interpreted as model‑based UIs s derived from training residuals, rather than empirically calibrated 95% prediction intervals.

## Table 1. The VLW and VLW/GDP for alcohol-attributable diseases in China between 1990 and 2023 based on GBD 2023

| Diseases | Year | Sex | VLW ($ billion) | VLW 95% UI | VLW/GDP (%) | VLW/GDP (%) 95% UI |
| --- | --- | --- | --- | --- | --- | --- |
| AUD | 1990 | Female | 0.2800 | (0.1900,0.3975) | 0.0148 | (0.0100,0.0210) |
| AUD | 1990 | Male | 1.5364 | (1.0358,2.1903) | 0.0812 | (0.0547,0.1157) |
| AUD | 1990 | Total | 1.8402 | (1.2628,2.6055) | 0.0972 | (0.0667,0.1377) |
| AUD | 1991 | Female | 0.3188 | (0.2154,0.4549) | 0.0154 | (0.0104,0.0220) |
| AUD | 1991 | Male | 1.7372 | (1.1790,2.4845) | 0.0839 | (0.0570,0.1201) |
| AUD | 1991 | Total | 2.0825 | (1.4266,2.9552) | 0.1006 | (0.0689,0.1428) |
| AUD | 1992 | Female | 0.3752 | (0.2543,0.5359) | 0.0159 | (0.0108,0.0227) |
| AUD | 1992 | Male | 2.0392 | (1.3755,2.9072) | 0.0862 | (0.0582,0.1229) |
| AUD | 1992 | Total | 2.4447 | (1.6596,3.4627) | 0.1034 | (0.0702,0.1464) |
| AUD | 1993 | Female | 0.4352 | (0.2923,0.6268) | 0.0162 | (0.0108,0.0233) |
| AUD | 1993 | Male | 2.3692 | (1.5948,3.3746) | 0.0879 | (0.0592,0.1252) |
| AUD | 1993 | Total | 2.8402 | (1.9251,4.0201) | 0.1054 | (0.0714,0.1492) |
| AUD | 1994 | Female | 0.4990 | (0.3362,0.7127) | 0.0164 | (0.0110,0.0234) |
| AUD | 1994 | Male | 2.7205 | (1.8372,3.8607) | 0.0893 | (0.0603,0.1267) |
| AUD | 1994 | Total | 3.2615 | (2.2140,4.6175) | 0.1070 | (0.0727,0.1515) |
| AUD | 1995 | Female | 0.5544 | (0.3728,0.7956) | 0.0164 | (0.0110,0.0235) |
| AUD | 1995 | Male | 3.0366 | (2.0428,4.3036) | 0.0898 | (0.0604,0.1272) |
| AUD | 1995 | Total | 3.6388 | (2.4540,5.1395) | 0.1076 | (0.0725,0.1519) |
| AUD | 1996 | Female | 0.6069 | (0.4046,0.8717) | 0.0163 | (0.0109,0.0234) |
| AUD | 1996 | Male | 3.3234 | (2.2422,4.6960) | 0.0893 | (0.0603,0.1262) |
| AUD | 1996 | Total | 3.9825 | (2.7012,5.6029) | 0.1070 | (0.0726,0.1506) |
| AUD | 1997 | Female | 0.6556 | (0.4365,0.9513) | 0.0161 | (0.0107,0.0234) |
| AUD | 1997 | Male | 3.5436 | (2.4070,4.9695) | 0.0871 | (0.0592,0.1222) |
| AUD | 1997 | Total | 4.2546 | (2.9052,5.9510) | 0.1046 | (0.0714,0.1463) |
| AUD | 1998 | Female | 0.6982 | (0.4676,1.0150) | 0.0159 | (0.0107,0.0231) |
| AUD | 1998 | Male | 3.7131 | (2.5943,5.1953) | 0.0846 | (0.0591,0.1184) |
| AUD | 1998 | Total | 4.4687 | (3.1246,6.2211) | 0.1018 | (0.0712,0.1418) |
| AUD | 1999 | Female | 0.7427 | (0.4987,1.0746) | 0.0157 | (0.0105,0.0227) |
| AUD | 1999 | Male | 3.8882 | (2.7618,5.4189) | 0.0822 | (0.0584,0.1146) |
| AUD | 1999 | Total | 4.6904 | (3.3254,6.5095) | 0.0992 | (0.0703,0.1377) |
| AUD | 2000 | Female | 0.7999 | (0.5368,1.1700) | 0.0156 | (0.0105,0.0228) |
| AUD | 2000 | Male | 4.1797 | (2.9962,5.7696) | 0.0814 | (0.0584,0.1124) |
| AUD | 2000 | Total | 5.0442 | (3.5887,6.9860) | 0.0983 | (0.0699,0.1361) |
| AUD | 2001 | Female | 0.8605 | (0.5742,1.2656) | 0.0155 | (0.0103,0.0228) |
| AUD | 2001 | Male | 4.5640 | (3.2969,6.2549) | 0.0821 | (0.0593,0.1125) |
| AUD | 2001 | Total | 5.4971 | (3.9624,7.5608) | 0.0989 | (0.0713,0.1360) |
| AUD | 2002 | Female | 0.9263 | (0.6184,1.3578) | 0.0152 | (0.0102,0.0224) |
| AUD | 2002 | Male | 5.0537 | (3.6665,6.9072) | 0.0832 | (0.0604,0.1137) |
| AUD | 2002 | Total | 6.0626 | (4.3864,8.2865) | 0.0998 | (0.0722,0.1364) |
| AUD | 2003 | Female | 1.0034 | (0.6723,1.4844) | 0.0150 | (0.0101,0.0222) |
| AUD | 2003 | Male | 5.6976 | (4.1734,7.6692) | 0.0852 | (0.0624,0.1146) |
| AUD | 2003 | Total | 6.7977 | (4.9525,9.2027) | 0.1016 | (0.0740,0.1376) |
| AUD | 2004 | Female | 1.0870 | (0.7188,1.6059) | 0.0148 | (0.0098,0.0218) |
| AUD | 2004 | Male | 6.3979 | (4.7450,8.5608) | 0.0868 | (0.0644,0.1162) |
| AUD | 2004 | Total | 7.5963 | (5.6036,10.2433) | 0.1031 | (0.0761,0.1390) |
| AUD | 2005 | Female | 1.2015 | (0.7929,1.7821) | 0.0146 | (0.0097,0.0217) |
| AUD | 2005 | Male | 7.2223 | (5.3681,9.5395) | 0.0880 | (0.0654,0.1162) |
| AUD | 2005 | Total | 8.5523 | (6.3143,11.4220) | 0.1042 | (0.0769,0.1391) |
| AUD | 2006 | Female | 1.3398 | (0.8764,1.9821) | 0.0145 | (0.0095,0.0214) |
| AUD | 2006 | Male | 8.1833 | (6.0881,10.8048) | 0.0884 | (0.0658,0.1168) |
| AUD | 2006 | Total | 9.6701 | (7.1567,12.8773) | 0.1045 | (0.0774,0.1392) |
| AUD | 2007 | Female | 1.5234 | (0.9780,2.2779) | 0.0144 | (0.0093,0.0216) |
| AUD | 2007 | Male | 9.3301 | (6.9274,12.4278) | 0.0883 | (0.0656,0.1177) |
| AUD | 2007 | Total | 11.0209 | (8.1281,14.7314) | 0.1044 | (0.0770,0.1395) |
| AUD | 2008 | Female | 1.6731 | (1.0670,2.4897) | 0.0144 | (0.0092,0.0215) |
| AUD | 2008 | Male | 10.1601 | (7.5650,13.4998) | 0.0877 | (0.0653,0.1166) |
| AUD | 2008 | Total | 12.0187 | (8.8876,16.0513) | 0.1038 | (0.0767,0.1386) |
| AUD | 2009 | Female | 1.8366 | (1.1662,2.7309) | 0.0145 | (0.0092,0.0216) |
| AUD | 2009 | Male | 11.0400 | (8.2770,14.6434) | 0.0871 | (0.0653,0.1156) |
| AUD | 2009 | Total | 13.0847 | (9.6464,17.5101) | 0.1033 | (0.0761,0.1382) |
| AUD | 2010 | Female | 2.0340 | (1.2909,3.0357) | 0.0145 | (0.0092,0.0217) |
| AUD | 2010 | Male | 12.0898 | (9.0130,15.9737) | 0.0863 | (0.0643,0.1140) |
| AUD | 2010 | Total | 14.3496 | (10.5982,19.1280) | 0.1024 | (0.0756,0.1365) |
| AUD | 2011 | Female | 2.2333 | (1.4083,3.3677) | 0.0146 | (0.0092,0.0220) |
| AUD | 2011 | Male | 13.1330 | (9.6851,17.4075) | 0.0856 | (0.0631,0.1135) |
| AUD | 2011 | Total | 15.6221 | (11.3780,20.9894) | 0.1018 | (0.0742,0.1368) |
| AUD | 2012 | Female | 2.4232 | (1.5387,3.6396) | 0.0146 | (0.0093,0.0220) |
| AUD | 2012 | Male | 14.2084 | (10.3739,18.8521) | 0.0859 | (0.0627,0.1139) |
| AUD | 2012 | Total | 16.9189 | (12.1738,22.7190) | 0.1023 | (0.0736,0.1373) |
| AUD | 2013 | Female | 2.6272 | (1.6500,3.9331) | 0.0147 | (0.0093,0.0221) |
| AUD | 2013 | Male | 15.3577 | (11.0892,20.5075) | 0.0861 | (0.0622,0.1150) |
| AUD | 2013 | Total | 18.3053 | (13.0711,24.7699) | 0.1027 | (0.0733,0.1389) |
| AUD | 2014 | Female | 2.8426 | (1.7770,4.2621) | 0.0148 | (0.0093,0.0222) |
| AUD | 2014 | Male | 16.6863 | (12.0769,22.4422) | 0.0871 | (0.0630,0.1171) |
| AUD | 2014 | Total | 19.8942 | (14.1748,27.0189) | 0.1038 | (0.0740,0.1410) |
| AUD | 2015 | Female | 3.0448 | (1.9042,4.5855) | 0.0149 | (0.0093,0.0224) |
| AUD | 2015 | Male | 18.1227 | (13.1458,24.5275) | 0.0884 | (0.0641,0.1196) |
| AUD | 2015 | Total | 21.5771 | (15.4080,29.4058) | 0.1053 | (0.0752,0.1434) |
| AUD | 2016 | Female | 3.2010 | (2.0087,4.8167) | 0.0146 | (0.0092,0.0220) |
| AUD | 2016 | Male | 19.7386 | (14.3397,26.8568) | 0.0902 | (0.0655,0.1227) |
| AUD | 2016 | Total | 23.3983 | (16.7369,32.0275) | 0.1069 | (0.0765,0.1463) |
| AUD | 2017 | Female | 3.3016 | (2.0860,4.8969) | 0.0141 | (0.0089,0.0209) |
| AUD | 2017 | Male | 21.4986 | (15.4850,29.2990) | 0.0919 | (0.0662,0.1252) |
| AUD | 2017 | Total | 25.3317 | (18.0692,34.8372) | 0.1083 | (0.0772,0.1489) |
| AUD | 2018 | Female | 3.3503 | (2.1345,4.9755) | 0.0134 | (0.0085,0.0199) |
| AUD | 2018 | Male | 23.2146 | (16.6883,31.5282) | 0.0929 | (0.0668,0.1262) |
| AUD | 2018 | Total | 27.1073 | (19.3042,37.2336) | 0.1085 | (0.0773,0.1491) |
| AUD | 2019 | Female | 3.3920 | (2.1425,5.0481) | 0.0128 | (0.0081,0.0191) |
| AUD | 2019 | Male | 24.6884 | (17.6294,33.7802) | 0.0932 | (0.0665,0.1275) |
| AUD | 2019 | Total | 28.6348 | (20.1363,39.5323) | 0.1081 | (0.0760,0.1492) |
| AUD | 2020 | Female | 3.3854 | (2.1438,5.0449) | 0.0125 | (0.0079,0.0186) |
| AUD | 2020 | Male | 25.1359 | (17.7650,34.4550) | 0.0927 | (0.0655,0.1271) |
| AUD | 2020 | Total | 29.1063 | (20.3749,40.1712) | 0.1073 | (0.0751,0.1482) |
| AUD | 2021 | Female | 3.6714 | (2.3090,5.4724) | 0.0125 | (0.0078,0.0186) |
| AUD | 2021 | Male | 27.5653 | (19.6366,37.8486) | 0.0936 | (0.0667,0.1286) |
| AUD | 2021 | Total | 31.8449 | (22.4811,43.8873) | 0.1082 | (0.0764,0.1491) |
| AUD | 2022 | Female | 3.8114 | (2.3870,5.6405) | 0.0126 | (0.0079,0.0186) |
| AUD | 2022 | Male | 28.6317 | (20.2257,39.3379) | 0.0943 | (0.0666,0.1296) |
| AUD | 2022 | Total | 33.1204 | (23.2486,45.8511) | 0.1091 | (0.0766,0.1510) |
| AUD | 2023 | Female | 3.9890 | (2.5072,5.9359) | 0.0125 | (0.0078,0.0185) |
| AUD | 2023 | Male | 27.7565 | (19.5493,38.0646) | 0.0867 | (0.0611,0.1189) |
| AUD | 2023 | Total | 32.3951 | (22.7208,44.7071) | 0.1012 | (0.0710,0.1397) |
| ACM | 1990 | Female | 0.0069 | (0.0012,0.0278) | 0.0004 | (0.0001,0.0015) |
| ACM | 1990 | Male | 0.0335 | (0.0092,0.1115) | 0.0018 | (0.0005,0.0059) |
| ACM | 1990 | Total | 0.0414 | (0.0123,0.1337) | 0.0022 | (0.0006,0.0071) |
| ACM | 1991 | Female | 0.0074 | (0.0013,0.0316) | 0.0004 | (0.0001,0.0015) |
| ACM | 1991 | Male | 0.0370 | (0.0102,0.1234) | 0.0018 | (0.0005,0.0060) |
| ACM | 1991 | Total | 0.0455 | (0.0139,0.1469) | 0.0022 | (0.0007,0.0071) |
| ACM | 1992 | Female | 0.0083 | (0.0015,0.0361) | 0.0004 | (0.0001,0.0015) |
| ACM | 1992 | Male | 0.0425 | (0.0117,0.1410) | 0.0018 | (0.0005,0.0060) |
| ACM | 1992 | Total | 0.0520 | (0.0161,0.1698) | 0.0022 | (0.0007,0.0072) |
| ACM | 1993 | Female | 0.0090 | (0.0017,0.0386) | 0.0003 | (0.0001,0.0014) |
| ACM | 1993 | Male | 0.0482 | (0.0134,0.1640) | 0.0018 | (0.0005,0.0061) |
| ACM | 1993 | Total | 0.0587 | (0.0182,0.1943) | 0.0022 | (0.0007,0.0072) |
| ACM | 1994 | Female | 0.0099 | (0.0020,0.0404) | 0.0003 | (0.0001,0.0013) |
| ACM | 1994 | Male | 0.0548 | (0.0149,0.1886) | 0.0018 | (0.0005,0.0062) |
| ACM | 1994 | Total | 0.0662 | (0.0204,0.2213) | 0.0022 | (0.0007,0.0073) |
| ACM | 1995 | Female | 0.0106 | (0.0022,0.0410) | 0.0003 | (0.0001,0.0012) |
| ACM | 1995 | Male | 0.0599 | (0.0159,0.2084) | 0.0018 | (0.0005,0.0062) |
| ACM | 1995 | Total | 0.0723 | (0.0225,0.2447) | 0.0021 | (0.0007,0.0072) |
| ACM | 1996 | Female | 0.0114 | (0.0025,0.0421) | 0.0003 | (0.0001,0.0011) |
| ACM | 1996 | Male | 0.0644 | (0.0168,0.2228) | 0.0017 | (0.0005,0.0060) |
| ACM | 1996 | Total | 0.0776 | (0.0242,0.2598) | 0.0021 | (0.0007,0.0070) |
| ACM | 1997 | Female | 0.0122 | (0.0029,0.0451) | 0.0003 | (0.0001,0.0011) |
| ACM | 1997 | Male | 0.0687 | (0.0182,0.2353) | 0.0017 | (0.0004,0.0058) |
| ACM | 1997 | Total | 0.0828 | (0.0265,0.2736) | 0.0020 | (0.0007,0.0067) |
| ACM | 1998 | Female | 0.0131 | (0.0033,0.0476) | 0.0003 | (0.0001,0.0011) |
| ACM | 1998 | Male | 0.0718 | (0.0193,0.2501) | 0.0016 | (0.0004,0.0057) |
| ACM | 1998 | Total | 0.0870 | (0.0286,0.2902) | 0.0020 | (0.0007,0.0066) |
| ACM | 1999 | Female | 0.0141 | (0.0036,0.0497) | 0.0003 | (0.0001,0.0011) |
| ACM | 1999 | Male | 0.0750 | (0.0206,0.2627) | 0.0016 | (0.0004,0.0056) |
| ACM | 1999 | Total | 0.0913 | (0.0303,0.3079) | 0.0019 | (0.0006,0.0065) |
| ACM | 2000 | Female | 0.0153 | (0.0041,0.0528) | 0.0003 | (0.0001,0.0010) |
| ACM | 2000 | Male | 0.0789 | (0.0217,0.2730) | 0.0015 | (0.0004,0.0053) |
| ACM | 2000 | Total | 0.0966 | (0.0334,0.3208) | 0.0019 | (0.0007,0.0062) |
| ACM | 2001 | Female | 0.0163 | (0.0045,0.0558) | 0.0003 | (0.0001,0.0010) |
| ACM | 2001 | Male | 0.0825 | (0.0226,0.2824) | 0.0015 | (0.0004,0.0051) |
| ACM | 2001 | Total | 0.1013 | (0.0358,0.3346) | 0.0018 | (0.0006,0.0060) |
| ACM | 2002 | Female | 0.0175 | (0.0051,0.0589) | 0.0003 | (0.0001,0.0010) |
| ACM | 2002 | Male | 0.0873 | (0.0247,0.2941) | 0.0014 | (0.0004,0.0048) |
| ACM | 2002 | Total | 0.1075 | (0.0379,0.3487) | 0.0018 | (0.0006,0.0057) |
| ACM | 2003 | Female | 0.0193 | (0.0060,0.0629) | 0.0003 | (0.0001,0.0009) |
| ACM | 2003 | Male | 0.0972 | (0.0309,0.3161) | 0.0015 | (0.0005,0.0047) |
| ACM | 2003 | Total | 0.1196 | (0.0447,0.3688) | 0.0018 | (0.0007,0.0055) |
| ACM | 2004 | Female | 0.0207 | (0.0066,0.0662) | 0.0003 | (0.0001,0.0009) |
| ACM | 2004 | Male | 0.1064 | (0.0365,0.3361) | 0.0014 | (0.0005,0.0046) |
| ACM | 2004 | Total | 0.1306 | (0.0514,0.3897) | 0.0018 | (0.0007,0.0053) |
| ACM | 2005 | Female | 0.0228 | (0.0075,0.0728) | 0.0003 | (0.0001,0.0009) |
| ACM | 2005 | Male | 0.1247 | (0.0450,0.3808) | 0.0015 | (0.0005,0.0046) |
| ACM | 2005 | Total | 0.1515 | (0.0636,0.4356) | 0.0018 | (0.0008,0.0053) |
| ACM | 2006 | Female | 0.0254 | (0.0085,0.0803) | 0.0003 | (0.0001,0.0009) |
| ACM | 2006 | Male | 0.1530 | (0.0554,0.4200) | 0.0017 | (0.0006,0.0045) |
| ACM | 2006 | Total | 0.1834 | (0.0812,0.4870) | 0.0020 | (0.0009,0.0053) |
| ACM | 2007 | Female | 0.0279 | (0.0094,0.0855) | 0.0003 | (0.0001,0.0008) |
| ACM | 2007 | Male | 0.1892 | (0.0648,0.4630) | 0.0018 | (0.0006,0.0044) |
| ACM | 2007 | Total | 0.2235 | (0.0974,0.5271) | 0.0021 | (0.0009,0.0050) |
| ACM | 2008 | Female | 0.0294 | (0.0097,0.0861) | 0.0003 | (0.0001,0.0007) |
| ACM | 2008 | Male | 0.2240 | (0.0713,0.4999) | 0.0019 | (0.0006,0.0043) |
| ACM | 2008 | Total | 0.2612 | (0.1047,0.5646) | 0.0023 | (0.0009,0.0049) |
| ACM | 2009 | Female | 0.0303 | (0.0100,0.0855) | 0.0002 | (0.0001,0.0007) |
| ACM | 2009 | Male | 0.2566 | (0.0772,0.5466) | 0.0020 | (0.0006,0.0043) |
| ACM | 2009 | Total | 0.2964 | (0.1117,0.6077) | 0.0023 | (0.0009,0.0048) |
| ACM | 2010 | Female | 0.0308 | (0.0102,0.0848) | 0.0002 | (0.0001,0.0006) |
| ACM | 2010 | Male | 0.2919 | (0.0821,0.6051) | 0.0021 | (0.0006,0.0043) |
| ACM | 2010 | Total | 0.3335 | (0.1202,0.6626) | 0.0024 | (0.0009,0.0047) |
| ACM | 2011 | Female | 0.0308 | (0.0103,0.0828) | 0.0002 | (0.0001,0.0005) |
| ACM | 2011 | Male | 0.3245 | (0.0869,0.6530) | 0.0021 | (0.0006,0.0043) |
| ACM | 2011 | Total | 0.3676 | (0.1251,0.7148) | 0.0024 | (0.0008,0.0047) |
| ACM | 2012 | Female | 0.0300 | (0.0101,0.0769) | 0.0002 | (0.0001,0.0005) |
| ACM | 2012 | Male | 0.3504 | (0.0918,0.6909) | 0.0021 | (0.0006,0.0042) |
| ACM | 2012 | Total | 0.3940 | (0.1278,0.7554) | 0.0024 | (0.0008,0.0046) |
| ACM | 2013 | Female | 0.0293 | (0.0098,0.0720) | 0.0002 | (0.0001,0.0004) |
| ACM | 2013 | Male | 0.3747 | (0.0968,0.7223) | 0.0021 | (0.0005,0.0041) |
| ACM | 2013 | Total | 0.4188 | (0.1301,0.7864) | 0.0023 | (0.0007,0.0044) |
| ACM | 2014 | Female | 0.0288 | (0.0097,0.0678) | 0.0002 | (0.0001,0.0004) |
| ACM | 2014 | Male | 0.3939 | (0.1001,0.7402) | 0.0021 | (0.0005,0.0039) |
| ACM | 2014 | Total | 0.4389 | (0.1330,0.8106) | 0.0023 | (0.0007,0.0042) |
| ACM | 2015 | Female | 0.0286 | (0.0095,0.0658) | 0.0001 | (0.0000,0.0003) |
| ACM | 2015 | Male | 0.4128 | (0.1058,0.7702) | 0.0020 | (0.0005,0.0038) |
| ACM | 2015 | Total | 0.4589 | (0.1372,0.8400) | 0.0022 | (0.0007,0.0041) |
| ACM | 2016 | Female | 0.0293 | (0.0096,0.0693) | 0.0001 | (0.0000,0.0003) |
| ACM | 2016 | Male | 0.4346 | (0.1130,0.8190) | 0.0020 | (0.0005,0.0037) |
| ACM | 2016 | Total | 0.4828 | (0.1461,0.8894) | 0.0022 | (0.0007,0.0041) |
| ACM | 2017 | Female | 0.0295 | (0.0096,0.0684) | 0.0001 | (0.0000,0.0003) |
| ACM | 2017 | Male | 0.4456 | (0.1190,0.8443) | 0.0019 | (0.0005,0.0036) |
| ACM | 2017 | Total | 0.4954 | (0.1541,0.9170) | 0.0021 | (0.0007,0.0039) |
| ACM | 2018 | Female | 0.0289 | (0.0092,0.0675) | 0.0001 | (0.0000,0.0003) |
| ACM | 2018 | Male | 0.4357 | (0.1228,0.8278) | 0.0017 | (0.0005,0.0033) |
| ACM | 2018 | Total | 0.4837 | (0.1565,0.8967) | 0.0019 | (0.0006,0.0036) |
| ACM | 2019 | Female | 0.0280 | (0.0088,0.0654) | 0.0001 | (0.0000,0.0002) |
| ACM | 2019 | Male | 0.4195 | (0.1214,0.8227) | 0.0016 | (0.0005,0.0031) |
| ACM | 2019 | Total | 0.4652 | (0.1538,0.8957) | 0.0018 | (0.0006,0.0034) |
| ACM | 2020 | Female | 0.0270 | (0.0084,0.0632) | 0.0001 | (0.0000,0.0002) |
| ACM | 2020 | Male | 0.4005 | (0.1181,0.8162) | 0.0015 | (0.0004,0.0030) |
| ACM | 2020 | Total | 0.4447 | (0.1485,0.8859) | 0.0016 | (0.0005,0.0033) |
| ACM | 2021 | Female | 0.0299 | (0.0089,0.0712) | 0.0001 | (0.0000,0.0002) |
| ACM | 2021 | Male | 0.4486 | (0.1312,0.9279) | 0.0015 | (0.0004,0.0032) |
| ACM | 2021 | Total | 0.4962 | (0.1660,1.0062) | 0.0017 | (0.0006,0.0034) |
| ACM | 2022 | Female | 0.0326 | (0.0093,0.0787) | 0.0001 | (0.0000,0.0003) |
| ACM | 2022 | Male | 0.4838 | (0.1425,1.0153) | 0.0016 | (0.0005,0.0033) |
| ACM | 2022 | Total | 0.5372 | (0.1721,1.0893) | 0.0018 | (0.0006,0.0036) |
| ACM | 2023 | Female | 0.0337 | (0.0094,0.0825) | 0.0001 | (0.0000,0.0003) |
| ACM | 2023 | Male | 0.4754 | (0.1389,1.0420) | 0.0015 | (0.0004,0.0033) |
| ACM | 2023 | Total | 0.5287 | (0.1701,1.1140) | 0.0017 | (0.0005,0.0035) |
| ALD | 1990 | Female | 0.0309 | (0.0174,0.0507) | 0.0016 | (0.0009,0.0027) |
| ALD | 1990 | Male | 0.1784 | (0.1015,0.3050) | 0.0094 | (0.0054,0.0161) |
| ALD | 1990 | Total | 0.2174 | (0.1296,0.3550) | 0.0115 | (0.0069,0.0188) |
| ALD | 1991 | Female | 0.0319 | (0.0181,0.0517) | 0.0015 | (0.0009,0.0025) |
| ALD | 1991 | Male | 0.1917 | (0.1116,0.3167) | 0.0093 | (0.0054,0.0153) |
| ALD | 1991 | Total | 0.2331 | (0.1419,0.3716) | 0.0113 | (0.0069,0.0180) |
| ALD | 1992 | Female | 0.0347 | (0.0199,0.0553) | 0.0015 | (0.0008,0.0023) |
| ALD | 1992 | Male | 0.2163 | (0.1299,0.3449) | 0.0091 | (0.0055,0.0146) |
| ALD | 1992 | Total | 0.2623 | (0.1621,0.4072) | 0.0111 | (0.0069,0.0172) |
| ALD | 1993 | Female | 0.0379 | (0.0218,0.0601) | 0.0014 | (0.0008,0.0022) |
| ALD | 1993 | Male | 0.2434 | (0.1467,0.3804) | 0.0090 | (0.0054,0.0141) |
| ALD | 1993 | Total | 0.2949 | (0.1834,0.4486) | 0.0109 | (0.0068,0.0166) |
| ALD | 1994 | Female | 0.0418 | (0.0241,0.0659) | 0.0014 | (0.0008,0.0022) |
| ALD | 1994 | Male | 0.2730 | (0.1682,0.4192) | 0.0090 | (0.0055,0.0138) |
| ALD | 1994 | Total | 0.3307 | (0.2098,0.4922) | 0.0109 | (0.0069,0.0162) |
| ALD | 1995 | Female | 0.0452 | (0.0264,0.0711) | 0.0013 | (0.0008,0.0021) |
| ALD | 1995 | Male | 0.3031 | (0.1894,0.4570) | 0.0090 | (0.0056,0.0135) |
| ALD | 1995 | Total | 0.3670 | (0.2355,0.5379) | 0.0108 | (0.0070,0.0159) |
| ALD | 1996 | Female | 0.0490 | (0.0291,0.0762) | 0.0013 | (0.0008,0.0020) |
| ALD | 1996 | Male | 0.3338 | (0.2124,0.5036) | 0.0090 | (0.0057,0.0135) |
| ALD | 1996 | Total | 0.4036 | (0.2621,0.5922) | 0.0108 | (0.0070,0.0159) |
| ALD | 1997 | Female | 0.0527 | (0.0318,0.0821) | 0.0013 | (0.0008,0.0020) |
| ALD | 1997 | Male | 0.3670 | (0.2351,0.5490) | 0.0090 | (0.0058,0.0135) |
| ALD | 1997 | Total | 0.4427 | (0.2889,0.6483) | 0.0109 | (0.0071,0.0159) |
| ALD | 1998 | Female | 0.0566 | (0.0344,0.0875) | 0.0013 | (0.0008,0.0020) |
| ALD | 1998 | Male | 0.4020 | (0.2545,0.5951) | 0.0092 | (0.0058,0.0136) |
| ALD | 1998 | Total | 0.4840 | (0.3145,0.6984) | 0.0110 | (0.0072,0.0159) |
| ALD | 1999 | Female | 0.0611 | (0.0385,0.0930) | 0.0013 | (0.0008,0.0020) |
| ALD | 1999 | Male | 0.4417 | (0.2854,0.6564) | 0.0093 | (0.0060,0.0139) |
| ALD | 1999 | Total | 0.5303 | (0.3506,0.7755) | 0.0112 | (0.0074,0.0164) |
| ALD | 2000 | Female | 0.0659 | (0.0419,0.0988) | 0.0013 | (0.0008,0.0019) |
| ALD | 2000 | Male | 0.4905 | (0.3241,0.7174) | 0.0096 | (0.0063,0.0140) |
| ALD | 2000 | Total | 0.5865 | (0.3919,0.8419) | 0.0114 | (0.0076,0.0164) |
| ALD | 2001 | Female | 0.0700 | (0.0447,0.1043) | 0.0013 | (0.0008,0.0019) |
| ALD | 2001 | Male | 0.5344 | (0.3567,0.7786) | 0.0096 | (0.0064,0.0140) |
| ALD | 2001 | Total | 0.6369 | (0.4333,0.9090) | 0.0115 | (0.0078,0.0163) |
| ALD | 2002 | Female | 0.0754 | (0.0481,0.1109) | 0.0012 | (0.0008,0.0018) |
| ALD | 2002 | Male | 0.5893 | (0.4020,0.8552) | 0.0097 | (0.0066,0.0141) |
| ALD | 2002 | Total | 0.7006 | (0.4865,0.9976) | 0.0115 | (0.0080,0.0164) |
| ALD | 2003 | Female | 0.0825 | (0.0530,0.1207) | 0.0012 | (0.0008,0.0018) |
| ALD | 2003 | Male | 0.6589 | (0.4594,0.9357) | 0.0098 | (0.0069,0.0140) |
| ALD | 2003 | Total | 0.7807 | (0.5552,1.0891) | 0.0117 | (0.0083,0.0163) |
| ALD | 2004 | Female | 0.0911 | (0.0600,0.1307) | 0.0012 | (0.0008,0.0018) |
| ALD | 2004 | Male | 0.7408 | (0.5274,1.0357) | 0.0101 | (0.0072,0.0141) |
| ALD | 2004 | Total | 0.8766 | (0.6342,1.2064) | 0.0119 | (0.0086,0.0164) |
| ALD | 2005 | Female | 0.0998 | (0.0667,0.1431) | 0.0012 | (0.0008,0.0017) |
| ALD | 2005 | Male | 0.8230 | (0.5934,1.1278) | 0.0100 | (0.0072,0.0137) |
| ALD | 2005 | Total | 0.9733 | (0.7086,1.3241) | 0.0119 | (0.0086,0.0161) |
| ALD | 2006 | Female | 0.1089 | (0.0739,0.1550) | 0.0012 | (0.0008,0.0017) |
| ALD | 2006 | Male | 0.9121 | (0.6547,1.2261) | 0.0099 | (0.0071,0.0133) |
| ALD | 2006 | Total | 1.0776 | (0.7840,1.4370) | 0.0116 | (0.0085,0.0155) |
| ALD | 2007 | Female | 0.1193 | (0.0812,0.1689) | 0.0011 | (0.0008,0.0016) |
| ALD | 2007 | Male | 1.0229 | (0.7415,1.3739) | 0.0097 | (0.0070,0.0130) |
| ALD | 2007 | Total | 1.2051 | (0.8805,1.6058) | 0.0114 | (0.0083,0.0152) |
| ALD | 2008 | Female | 0.1249 | (0.0863,0.1739) | 0.0011 | (0.0007,0.0015) |
| ALD | 2008 | Male | 1.0966 | (0.8020,1.4612) | 0.0095 | (0.0069,0.0126) |
| ALD | 2008 | Total | 1.2892 | (0.9515,1.7145) | 0.0111 | (0.0082,0.0148) |
| ALD | 2009 | Female | 0.1316 | (0.0928,0.1829) | 0.0010 | (0.0007,0.0014) |
| ALD | 2009 | Male | 1.1592 | (0.8522,1.5298) | 0.0091 | (0.0067,0.0121) |
| ALD | 2009 | Total | 1.3647 | (1.0091,1.7920) | 0.0108 | (0.0080,0.0141) |
| ALD | 2010 | Female | 0.1395 | (0.0980,0.1931) | 0.0010 | (0.0007,0.0014) |
| ALD | 2010 | Male | 1.2404 | (0.9110,1.6333) | 0.0089 | (0.0065,0.0117) |
| ALD | 2010 | Total | 1.4583 | (1.0785,1.8976) | 0.0104 | (0.0077,0.0135) |
| ALD | 2011 | Female | 0.1502 | (0.1045,0.2085) | 0.0010 | (0.0007,0.0014) |
| ALD | 2011 | Male | 1.3414 | (0.9832,1.7810) | 0.0087 | (0.0064,0.0116) |
| ALD | 2011 | Total | 1.5811 | (1.1603,2.0811) | 0.0103 | (0.0076,0.0136) |
| ALD | 2012 | Female | 0.1630 | (0.1131,0.2296) | 0.0010 | (0.0007,0.0014) |
| ALD | 2012 | Male | 1.5003 | (1.0900,2.0091) | 0.0091 | (0.0066,0.0121) |
| ALD | 2012 | Total | 1.7676 | (1.2854,2.3451) | 0.0107 | (0.0078,0.0142) |
| ALD | 2013 | Female | 0.1754 | (0.1218,0.2470) | 0.0010 | (0.0007,0.0014) |
| ALD | 2013 | Male | 1.6526 | (1.1972,2.2055) | 0.0093 | (0.0067,0.0124) |
| ALD | 2013 | Total | 1.9433 | (1.4139,2.5781) | 0.0109 | (0.0079,0.0145) |
| ALD | 2014 | Female | 0.1911 | (0.1321,0.2695) | 0.0010 | (0.0007,0.0014) |
| ALD | 2014 | Male | 1.8289 | (1.3350,2.4347) | 0.0095 | (0.0070,0.0127) |
| ALD | 2014 | Total | 2.1490 | (1.5695,2.8404) | 0.0112 | (0.0082,0.0148) |
| ALD | 2015 | Female | 0.2035 | (0.1397,0.2857) | 0.0010 | (0.0007,0.0014) |
| ALD | 2015 | Male | 1.9881 | (1.4479,2.6705) | 0.0097 | (0.0071,0.0130) |
| ALD | 2015 | Total | 2.3335 | (1.7001,3.1087) | 0.0114 | (0.0083,0.0152) |
| ALD | 2016 | Female | 0.2144 | (0.1463,0.3023) | 0.0010 | (0.0007,0.0014) |
| ALD | 2016 | Male | 2.1371 | (1.5548,2.9109) | 0.0098 | (0.0071,0.0133) |
| ALD | 2016 | Total | 2.5059 | (1.8259,3.3898) | 0.0114 | (0.0083,0.0155) |
| ALD | 2017 | Female | 0.2252 | (0.1539,0.3119) | 0.0010 | (0.0007,0.0013) |
| ALD | 2017 | Male | 2.2542 | (1.6204,3.0806) | 0.0096 | (0.0069,0.0132) |
| ALD | 2017 | Total | 2.6492 | (1.9142,3.5936) | 0.0113 | (0.0082,0.0154) |
| ALD | 2018 | Female | 0.2346 | (0.1609,0.3251) | 0.0009 | (0.0006,0.0013) |
| ALD | 2018 | Male | 2.4086 | (1.7313,3.2780) | 0.0096 | (0.0069,0.0131) |
| ALD | 2018 | Total | 2.8177 | (2.0224,3.8230) | 0.0113 | (0.0081,0.0153) |
| ALD | 2019 | Female | 0.2426 | (0.1683,0.3363) | 0.0009 | (0.0006,0.0013) |
| ALD | 2019 | Male | 2.5381 | (1.8344,3.4332) | 0.0096 | (0.0069,0.0130) |
| ALD | 2019 | Total | 2.9616 | (2.1442,3.9812) | 0.0112 | (0.0081,0.0150) |
| ALD | 2020 | Female | 0.2474 | (0.1704,0.3426) | 0.0009 | (0.0006,0.0013) |
| ALD | 2020 | Male | 2.5837 | (1.8779,3.4774) | 0.0095 | (0.0069,0.0128) |
| ALD | 2020 | Total | 3.0234 | (2.2061,4.0533) | 0.0112 | (0.0081,0.0149) |
| ALD | 2021 | Female | 0.2705 | (0.1800,0.3877) | 0.0009 | (0.0006,0.0013) |
| ALD | 2021 | Male | 2.8621 | (2.0388,3.8641) | 0.0097 | (0.0069,0.0131) |
| ALD | 2021 | Total | 3.3324 | (2.3827,4.4778) | 0.0113 | (0.0081,0.0152) |
| ALD | 2022 | Female | 0.2898 | (0.1858,0.4317) | 0.0010 | (0.0006,0.0014) |
| ALD | 2022 | Male | 3.0052 | (1.9597,4.3663) | 0.0099 | (0.0065,0.0144) |
| ALD | 2022 | Total | 3.5160 | (2.3138,5.0740) | 0.0116 | (0.0076,0.0167) |
| ALD | 2023 | Female | 0.2780 | (0.1718,0.4216) | 0.0009 | (0.0005,0.0013) |
| ALD | 2023 | Male | 2.7835 | (1.8003,4.1785) | 0.0087 | (0.0056,0.0131) |
| ALD | 2023 | Total | 3.2616 | (2.1386,4.8060) | 0.0102 | (0.0067,0.0150) |
| LCA | 1990 | Female | 0.0273 | (0.0170,0.0403) | 0.0014 | (0.0009,0.0021) |
| LCA | 1990 | Male | 0.0618 | (0.0405,0.0919) | 0.0033 | (0.0021,0.0049) |
| LCA | 1990 | Total | 0.0878 | (0.0592,0.1271) | 0.0046 | (0.0031,0.0067) |
| LCA | 1991 | Female | 0.0299 | (0.0185,0.0437) | 0.0014 | (0.0009,0.0021) |
| LCA | 1991 | Male | 0.0677 | (0.0445,0.1008) | 0.0033 | (0.0021,0.0049) |
| LCA | 1991 | Total | 0.0968 | (0.0653,0.1399) | 0.0047 | (0.0032,0.0068) |
| LCA | 1992 | Female | 0.0345 | (0.0217,0.0505) | 0.0015 | (0.0009,0.0021) |
| LCA | 1992 | Male | 0.0778 | (0.0512,0.1149) | 0.0033 | (0.0022,0.0049) |
| LCA | 1992 | Total | 0.1121 | (0.0754,0.1595) | 0.0047 | (0.0032,0.0067) |
| LCA | 1993 | Female | 0.0399 | (0.0256,0.0583) | 0.0015 | (0.0009,0.0022) |
| LCA | 1993 | Male | 0.0898 | (0.0584,0.1312) | 0.0033 | (0.0022,0.0049) |
| LCA | 1993 | Total | 0.1297 | (0.0869,0.1846) | 0.0048 | (0.0032,0.0069) |
| LCA | 1994 | Female | 0.0464 | (0.0302,0.0675) | 0.0015 | (0.0010,0.0022) |
| LCA | 1994 | Male | 0.1047 | (0.0680,0.1522) | 0.0034 | (0.0022,0.0050) |
| LCA | 1994 | Total | 0.1514 | (0.1024,0.2123) | 0.0050 | (0.0034,0.0070) |
| LCA | 1995 | Female | 0.0531 | (0.0348,0.0769) | 0.0016 | (0.0010,0.0023) |
| LCA | 1995 | Male | 0.1191 | (0.0776,0.1735) | 0.0035 | (0.0023,0.0051) |
| LCA | 1995 | Total | 0.1731 | (0.1161,0.2440) | 0.0051 | (0.0034,0.0072) |
| LCA | 1996 | Female | 0.0594 | (0.0393,0.0856) | 0.0016 | (0.0011,0.0023) |
| LCA | 1996 | Male | 0.1337 | (0.0870,0.1955) | 0.0036 | (0.0023,0.0053) |
| LCA | 1996 | Total | 0.1945 | (0.1314,0.2773) | 0.0052 | (0.0035,0.0075) |
| LCA | 1997 | Female | 0.0648 | (0.0432,0.0942) | 0.0016 | (0.0011,0.0023) |
| LCA | 1997 | Male | 0.1466 | (0.0971,0.2130) | 0.0036 | (0.0024,0.0052) |
| LCA | 1997 | Total | 0.2130 | (0.1442,0.3003) | 0.0052 | (0.0035,0.0074) |
| LCA | 1998 | Female | 0.0687 | (0.0461,0.0994) | 0.0016 | (0.0011,0.0023) |
| LCA | 1998 | Male | 0.1580 | (0.1049,0.2275) | 0.0036 | (0.0024,0.0052) |
| LCA | 1998 | Total | 0.2287 | (0.1559,0.3203) | 0.0052 | (0.0036,0.0073) |
| LCA | 1999 | Female | 0.0733 | (0.0494,0.1057) | 0.0016 | (0.0010,0.0022) |
| LCA | 1999 | Male | 0.1712 | (0.1147,0.2452) | 0.0036 | (0.0024,0.0052) |
| LCA | 1999 | Total | 0.2468 | (0.1698,0.3478) | 0.0052 | (0.0036,0.0074) |
| LCA | 2000 | Female | 0.0801 | (0.0534,0.1141) | 0.0016 | (0.0010,0.0022) |
| LCA | 2000 | Male | 0.1881 | (0.1258,0.2696) | 0.0037 | (0.0025,0.0053) |
| LCA | 2000 | Total | 0.2709 | (0.1878,0.3790) | 0.0053 | (0.0037,0.0074) |
| LCA | 2001 | Female | 0.0875 | (0.0592,0.1253) | 0.0016 | (0.0011,0.0023) |
| LCA | 2001 | Male | 0.2078 | (0.1387,0.2969) | 0.0037 | (0.0025,0.0053) |
| LCA | 2001 | Total | 0.2988 | (0.2057,0.4187) | 0.0054 | (0.0037,0.0075) |
| LCA | 2002 | Female | 0.0931 | (0.0630,0.1338) | 0.0015 | (0.0010,0.0022) |
| LCA | 2002 | Male | 0.2263 | (0.1502,0.3206) | 0.0037 | (0.0025,0.0053) |
| LCA | 2002 | Total | 0.3236 | (0.2222,0.4518) | 0.0053 | (0.0037,0.0074) |
| LCA | 2003 | Female | 0.0987 | (0.0665,0.1406) | 0.0015 | (0.0010,0.0021) |
| LCA | 2003 | Male | 0.2470 | (0.1643,0.3499) | 0.0037 | (0.0025,0.0052) |
| LCA | 2003 | Total | 0.3513 | (0.2397,0.4892) | 0.0053 | (0.0036,0.0073) |
| LCA | 2004 | Female | 0.1048 | (0.0713,0.1501) | 0.0014 | (0.0010,0.0020) |
| LCA | 2004 | Male | 0.2691 | (0.1796,0.3770) | 0.0037 | (0.0024,0.0051) |
| LCA | 2004 | Total | 0.3807 | (0.2624,0.5198) | 0.0052 | (0.0036,0.0071) |
| LCA | 2005 | Female | 0.1179 | (0.0804,0.1695) | 0.0014 | (0.0010,0.0021) |
| LCA | 2005 | Male | 0.3071 | (0.2085,0.4307) | 0.0037 | (0.0025,0.0052) |
| LCA | 2005 | Total | 0.4330 | (0.2995,0.5982) | 0.0053 | (0.0036,0.0073) |
| LCA | 2006 | Female | 0.1373 | (0.0938,0.1968) | 0.0015 | (0.0010,0.0021) |
| LCA | 2006 | Male | 0.3633 | (0.2459,0.5162) | 0.0039 | (0.0027,0.0056) |
| LCA | 2006 | Total | 0.5104 | (0.3527,0.7117) | 0.0055 | (0.0038,0.0077) |
| LCA | 2007 | Female | 0.1649 | (0.1130,0.2320) | 0.0016 | (0.0011,0.0022) |
| LCA | 2007 | Male | 0.4397 | (0.2957,0.6290) | 0.0042 | (0.0028,0.0060) |
| LCA | 2007 | Total | 0.6177 | (0.4283,0.8694) | 0.0058 | (0.0041,0.0082) |
| LCA | 2008 | Female | 0.1895 | (0.1294,0.2668) | 0.0016 | (0.0011,0.0023) |
| LCA | 2008 | Male | 0.5088 | (0.3390,0.7326) | 0.0044 | (0.0029,0.0063) |
| LCA | 2008 | Total | 0.7136 | (0.4913,1.0072) | 0.0062 | (0.0042,0.0087) |
| LCA | 2009 | Female | 0.2162 | (0.1470,0.3050) | 0.0017 | (0.0012,0.0024) |
| LCA | 2009 | Male | 0.5659 | (0.3801,0.8142) | 0.0045 | (0.0030,0.0064) |
| LCA | 2009 | Total | 0.8017 | (0.5501,1.1267) | 0.0063 | (0.0043,0.0089) |
| LCA | 2010 | Female | 0.2417 | (0.1637,0.3409) | 0.0017 | (0.0012,0.0024) |
| LCA | 2010 | Male | 0.6254 | (0.4185,0.8931) | 0.0045 | (0.0030,0.0064) |
| LCA | 2010 | Total | 0.8901 | (0.6094,1.2556) | 0.0064 | (0.0043,0.0090) |
| LCA | 2011 | Female | 0.2634 | (0.1807,0.3699) | 0.0017 | (0.0012,0.0024) |
| LCA | 2011 | Male | 0.6747 | (0.4544,0.9610) | 0.0044 | (0.0030,0.0063) |
| LCA | 2011 | Total | 0.9638 | (0.6586,1.3561) | 0.0063 | (0.0043,0.0088) |
| LCA | 2012 | Female | 0.2865 | (0.1958,0.4002) | 0.0017 | (0.0012,0.0024) |
| LCA | 2012 | Male | 0.7452 | (0.5032,1.0609) | 0.0045 | (0.0030,0.0064) |
| LCA | 2012 | Total | 1.0631 | (0.7257,1.5079) | 0.0064 | (0.0044,0.0091) |
| LCA | 2013 | Female | 0.3141 | (0.2135,0.4374) | 0.0018 | (0.0012,0.0025) |
| LCA | 2013 | Male | 0.8230 | (0.5490,1.1820) | 0.0046 | (0.0031,0.0066) |
| LCA | 2013 | Total | 1.1747 | (0.7998,1.6699) | 0.0066 | (0.0045,0.0094) |
| LCA | 2014 | Female | 0.3539 | (0.2404,0.4907) | 0.0018 | (0.0013,0.0026) |
| LCA | 2014 | Male | 0.9370 | (0.6322,1.3474) | 0.0049 | (0.0033,0.0070) |
| LCA | 2014 | Total | 1.3385 | (0.9146,1.8999) | 0.0070 | (0.0048,0.0099) |
| LCA | 2015 | Female | 0.3888 | (0.2629,0.5389) | 0.0019 | (0.0013,0.0026) |
| LCA | 2015 | Male | 1.0632 | (0.7149,1.5184) | 0.0052 | (0.0035,0.0074) |
| LCA | 2015 | Total | 1.5109 | (1.0366,2.1308) | 0.0074 | (0.0051,0.0104) |
| LCA | 2016 | Female | 0.4193 | (0.2814,0.5830) | 0.0019 | (0.0013,0.0027) |
| LCA | 2016 | Male | 1.1976 | (0.8037,1.7191) | 0.0055 | (0.0037,0.0079) |
| LCA | 2016 | Total | 1.6889 | (1.1559,2.3867) | 0.0077 | (0.0053,0.0109) |
| LCA | 2017 | Female | 0.4366 | (0.2979,0.6130) | 0.0019 | (0.0013,0.0026) |
| LCA | 2017 | Male | 1.2880 | (0.8588,1.8754) | 0.0055 | (0.0037,0.0080) |
| LCA | 2017 | Total | 1.8069 | (1.2278,2.5918) | 0.0077 | (0.0052,0.0111) |
| LCA | 2018 | Female | 0.4297 | (0.2955,0.6060) | 0.0017 | (0.0012,0.0024) |
| LCA | 2018 | Male | 1.3339 | (0.8946,1.9523) | 0.0053 | (0.0036,0.0078) |
| LCA | 2018 | Total | 1.8534 | (1.2600,2.6603) | 0.0074 | (0.0050,0.0107) |
| LCA | 2019 | Female | 0.4152 | (0.2872,0.5807) | 0.0016 | (0.0011,0.0022) |
| LCA | 2019 | Male | 1.3254 | (0.8966,1.8931) | 0.0050 | (0.0034,0.0071) |
| LCA | 2019 | Total | 1.8313 | (1.2548,2.5860) | 0.0069 | (0.0047,0.0098) |
| LCA | 2020 | Female | 0.4029 | (0.2794,0.5577) | 0.0015 | (0.0010,0.0021) |
| LCA | 2020 | Male | 1.2930 | (0.8844,1.8455) | 0.0048 | (0.0033,0.0068) |
| LCA | 2020 | Total | 1.7870 | (1.2435,2.5236) | 0.0066 | (0.0046,0.0093) |
| LCA | 2021 | Female | 0.4572 | (0.3102,0.6474) | 0.0016 | (0.0011,0.0022) |
| LCA | 2021 | Male | 1.5358 | (1.0467,2.2186) | 0.0052 | (0.0036,0.0075) |
| LCA | 2021 | Total | 2.0957 | (1.4459,2.9811) | 0.0071 | (0.0049,0.0101) |
| LCA | 2022 | Female | 0.5202 | (0.3359,0.7711) | 0.0017 | (0.0011,0.0025) |
| LCA | 2022 | Male | 1.7482 | (1.1295,2.6680) | 0.0058 | (0.0037,0.0088) |
| LCA | 2022 | Total | 2.3950 | (1.5836,3.5608) | 0.0079 | (0.0052,0.0117) |
| LCA | 2023 | Female | 0.5691 | (0.3498,0.8469) | 0.0018 | (0.0011,0.0026) |
| LCA | 2023 | Male | 1.7909 | (1.1041,2.8382) | 0.0056 | (0.0034,0.0089) |
| LCA | 2023 | Total | 2.4833 | (1.5943,3.8076) | 0.0078 | (0.0050,0.0119) |

Abbreviations: AUD, alcohol use disorders; ACM, alcoholic cardiomyopathy; ALD, cirrhosis and other chronic liver diseases due to alcohol use; LCA, liver cancer due to alcohol use; 95% UI, 95% uncertainty interval; VLW, value of lost welfare; GDP, gross domestic product.

## Table 2. Sensitivity analysis of VLW and VLW/GDP estimates for alcohol-attributable diseases in China, 1990–2023, based on GBD 2023 under the assumption of IE = 0.55

| year | AUD | |  | ACM | |  | ALD | |  | LCA | |
| --- | --- | --- | --- | --- | --- | --- | --- | --- | --- | --- | --- |
|  | VLW ($ billion) | VLW/GDP (%) |  | VLW ($ billion) | VLW/GDP (%) |  | VLW ($ billion) | VLW/GDP (%) |  | VLW ($ billion) | VLW/GDP (%) |
| 1990 | 5.735(3.935,8.120) | 0.303(0.208,0.429) |  | 0.129(0.038,0.417) | 0.007(0.002,0.022) |  | 0.677(0.404,1.106) | 0.036(0.021,0.059) |  | 0.274(0.184,0.396) | 0.015(0.010,0.021) |
| 1991 | 6.273(4.297,8.901) | 0.303(0.208,0.430) |  | 0.137(0.042,0.443) | 0.007(0.002,0.021) |  | 0.702(0.427,1.119) | 0.034(0.021,0.054) |  | 0.292(0.197,0.422) | 0.014(0.010,0.020) |
| 1992 | 6.972(4.733,9.875) | 0.295(0.200,0.418) |  | 0.148(0.046,0.484) | 0.006(0.002,0.021) |  | 0.748(0.462,1.161) | 0.032(0.020,0.049) |  | 0.320(0.215,0.455) | 0.014(0.009,0.019) |
| 1993 | 7.678(5.204,10.867) | 0.285(0.193,0.403) |  | 0.159(0.049,0.525) | 0.006(0.002,0.020) |  | 0.797(0.496,1.213) | 0.030(0.018,0.045) |  | 0.351(0.235,0.499) | 0.013(0.009,0.019) |
| 1994 | 8.385(5.692,11.871) | 0.275(0.187,0.390) |  | 0.170(0.053,0.569) | 0.006(0.002,0.019) |  | 0.850(0.539,1.265) | 0.028(0.018,0.042) |  | 0.389(0.263,0.546) | 0.013(0.009,0.018) |
| 1995 | 8.968(6.048,12.667) | 0.265(0.179,0.374) |  | 0.178(0.056,0.603) | 0.005(0.002,0.018) |  | 0.905(0.580,1.326) | 0.027(0.017,0.039) |  | 0.427(0.286,0.601) | 0.013(0.009,0.018) |
| 1996 | 9.449(6.409,13.293) | 0.254(0.172,0.357) |  | 0.184(0.057,0.616) | 0.005(0.002,0.017) |  | 0.958(0.622,1.405) | 0.026(0.017,0.038) |  | 0.461(0.312,0.658) | 0.012(0.008,0.018) |
| 1997 | 9.744(6.653,13.629) | 0.240(0.164,0.335) |  | 0.190(0.061,0.627) | 0.005(0.002,0.015) |  | 1.014(0.662,1.485) | 0.025(0.016,0.037) |  | 0.488(0.330,0.688) | 0.012(0.008,0.017) |
| 1998 | 9.932(6.944,13.826) | 0.226(0.158,0.315) |  | 0.193(0.064,0.645) | 0.004(0.002,0.015) |  | 1.076(0.699,1.552) | 0.025(0.016,0.035) |  | 0.508(0.346,0.712) | 0.012(0.008,0.016) |
| 1999 | 10.120(7.175,14.045) | 0.214(0.152,0.297) |  | 0.197(0.065,0.664) | 0.004(0.001,0.014) |  | 1.144(0.756,1.673) | 0.024(0.016,0.035) |  | 0.533(0.366,0.750) | 0.011(0.008,0.016) |
| 2000 | 10.525(7.488,14.576) | 0.205(0.146,0.284) |  | 0.202(0.070,0.669) | 0.004(0.001,0.013) |  | 1.224(0.818,1.756) | 0.024(0.016,0.034) |  | 0.565(0.392,0.791) | 0.011(0.008,0.015) |
| 2001 | 11.101(8.002,15.268) | 0.200(0.144,0.275) |  | 0.205(0.072,0.676) | 0.004(0.001,0.012) |  | 1.286(0.875,1.836) | 0.023(0.016,0.033) |  | 0.603(0.415,0.845) | 0.011(0.008,0.015) |
| 2002 | 11.801(8.538,16.130) | 0.194(0.141,0.266) |  | 0.209(0.074,0.679) | 0.003(0.001,0.011) |  | 1.364(0.947,1.942) | 0.022(0.016,0.032) |  | 0.630(0.432,0.880) | 0.010(0.007,0.015) |
| 2003 | 12.706(9.257,17.201) | 0.190(0.138,0.257) |  | 0.224(0.084,0.689) | 0.003(0.001,0.010) |  | 1.459(1.038,2.036) | 0.022(0.016,0.030) |  | 0.657(0.448,0.914) | 0.010(0.007,0.014) |
| 2004 | 13.631(10.056,18.381) | 0.185(0.137,0.250) |  | 0.234(0.092,0.699) | 0.003(0.001,0.010) |  | 1.573(1.138,2.165) | 0.021(0.015,0.029) |  | 0.683(0.471,0.933) | 0.009(0.006,0.013) |
| 2005 | 14.654(10.819,19.572) | 0.179(0.132,0.238) |  | 0.260(0.109,0.746) | 0.003(0.001,0.009) |  | 1.668(1.214,2.269) | 0.020(0.015,0.028) |  | 0.742(0.513,1.025) | 0.009(0.006,0.013) |
| 2006 | 15.743(11.651,20.964) | 0.170(0.126,0.227) |  | 0.299(0.132,0.793) | 0.003(0.001,0.009) |  | 1.754(1.276,2.339) | 0.019(0.014,0.025) |  | 0.831(0.574,1.159) | 0.009(0.006,0.013) |
| 2007 | 16.944(12.497,22.649) | 0.160(0.118,0.215) |  | 0.344(0.150,0.810) | 0.003(0.001,0.008) |  | 1.853(1.354,2.469) | 0.018(0.013,0.023) |  | 0.950(0.658,1.337) | 0.009(0.006,0.013) |
| 2008 | 17.768(13.139,23.729) | 0.153(0.113,0.205) |  | 0.386(0.155,0.835) | 0.003(0.001,0.007) |  | 1.906(1.407,2.535) | 0.017(0.012,0.022) |  | 1.055(0.726,1.489) | 0.009(0.006,0.013) |
| 2009 | 18.619(13.726,24.915) | 0.147(0.108,0.197) |  | 0.422(0.159,0.865) | 0.003(0.001,0.007) |  | 1.942(1.436,2.550) | 0.015(0.011,0.020) |  | 1.141(0.783,1.603) | 0.009(0.006,0.013) |
| 2010 | 19.556(14.444,26.068) | 0.140(0.103,0.186) |  | 0.455(0.164,0.903) | 0.003(0.001,0.006) |  | 1.987(1.470,2.586) | 0.014(0.011,0.019) |  | 1.213(0.830,1.711) | 0.009(0.006,0.012) |
| 2011 | 20.492(14.925,27.532) | 0.134(0.097,0.180) |  | 0.482(0.164,0.938) | 0.003(0.001,0.006) |  | 2.074(1.522,2.730) | 0.014(0.010,0.018) |  | 1.264(0.864,1.779) | 0.008(0.006,0.012) |
| 2012 | 21.516(15.482,28.892) | 0.130(0.094,0.175) |  | 0.501(0.162,0.961) | 0.003(0.001,0.006) |  | 2.248(1.635,2.982) | 0.014(0.010,0.018) |  | 1.352(0.923,1.918) | 0.008(0.006,0.012) |
| 2013 | 22.575(16.120,30.547) | 0.127(0.090,0.171) |  | 0.516(0.160,0.970) | 0.003(0.001,0.005) |  | 2.397(1.744,3.179) | 0.013(0.010,0.018) |  | 1.449(0.986,2.059) | 0.008(0.006,0.012) |
| 2014 | 23.820(16.972,32.351) | 0.124(0.089,0.169) |  | 0.526(0.159,0.971) | 0.003(0.001,0.005) |  | 2.573(1.879,3.401) | 0.013(0.010,0.018) |  | 1.603(1.095,2.275) | 0.008(0.006,0.012) |
| 2015 | 25.128(17.944,34.245) | 0.123(0.088,0.167) |  | 0.534(0.160,0.978) | 0.003(0.001,0.005) |  | 2.717(1.980,3.620) | 0.013(0.010,0.018) |  | 1.760(1.207,2.482) | 0.009(0.006,0.012) |
| 2016 | 26.525(18.974,36.308) | 0.121(0.087,0.166) |  | 0.547(0.166,1.008) | 0.003(0.001,0.005) |  | 2.841(2.070,3.843) | 0.013(0.010,0.018) |  | 1.915(1.310,2.706) | 0.009(0.006,0.012) |
| 2017 | 27.945(19.933,38.431) | 0.119(0.085,0.164) |  | 0.546(0.170,1.012) | 0.002(0.001,0.004) |  | 2.922(2.112,3.964) | 0.013(0.009,0.017) |  | 1.993(1.354,2.859) | 0.009(0.006,0.012) |
| 2018 | 29.098(20.722,39.967) | 0.117(0.083,0.160) |  | 0.519(0.168,0.963) | 0.002(0.001,0.004) |  | 3.025(2.171,4.104) | 0.012(0.009,0.016) |  | 1.990(1.353,2.856) | 0.008(0.005,0.011) |
| 2019 | 29.981(21.083,41.391) | 0.113(0.080,0.156) |  | 0.487(0.161,0.938) | 0.002(0.001,0.004) |  | 3.101(2.245,4.168) | 0.012(0.009,0.016) |  | 1.917(1.314,2.708) | 0.007(0.005,0.010) |
| 2020 | 30.191(21.134,41.669) | 0.111(0.078,0.154) |  | 0.461(0.154,0.919) | 0.002(0.001,0.003) |  | 3.136(2.288,4.204) | 0.012(0.008,0.016) |  | 1.854(1.290,2.618) | 0.007(0.005,0.010) |
| 2021 | 31.845(22.481,43.887) | 0.108(0.076,0.149) |  | 0.496(0.166,1.006) | 0.002(0.001,0.003) |  | 3.332(2.383,4.478) | 0.011(0.008,0.015) |  | 2.096(1.446,2.981) | 0.007(0.005,0.010) |
| 2022 | 32.662(22.927,45.216) | 0.108(0.076,0.149) |  | 0.530(0.170,1.074) | 0.002(0.001,0.004) |  | 3.467(2.282,5.004) | 0.011(0.008,0.017) |  | 2.362(1.562,3.511) | 0.008(0.005,0.012) |
| 2023 | 31.183(21.870,43.034) | 0.097(0.068,0.135) |  | 0.509(0.164,1.072) | 0.002(0.001,0.003) |  | 3.140(2.059,4.626) | 0.010(0.006,0.015) |  | 2.390(1.535,3.665) | 0.008(0.005,0.012) |

Abbreviations: AUD, alcohol use disorders; ACM, alcoholic cardiomyopathy; ALD, cirrhosis and other chronic liver diseases due to alcohol use; LCA, liver cancer due to alcohol use; VLW, value of lost welfare; GDP, gross domestic product.

## Table 3. Sensitivity analysis of VLW and VLW/GDP estimates for alcohol-attributable diseases in China, 1990–2023, based on GBD 2023 under the assumption of IE = 1.5

| year | AUD | |  | ACM | |  | ALD | |  | LCA | |
| --- | --- | --- | --- | --- | --- | --- | --- | --- | --- | --- | --- |
|  | VLW ($ billion) | VLW/GDP (%) |  | VLW ($ billion) | VLW/GDP (%) |  | VLW ($ billion) | VLW/GDP (%) |  | VLW ($ billion) | VLW/GDP (%) |
| 1990 | 0.520(0.357,0.737) | 0.028(0.019,0.039) |  | 0.012(0.003,0.038) | 0.001(0.000,0.002) |  | 0.061(0.037,0.100) | 0.003(0.002,0.005) |  | 0.025(0.017,0.036) | 0.001(0.001,0.002) |
| 1991 | 0.612(0.419,0.868) | 0.030(0.020,0.042) |  | 0.013(0.004,0.043) | 0.001(0.000,0.002) |  | 0.068(0.042,0.109) | 0.003(0.002,0.005) |  | 0.028(0.019,0.041) | 0.001(0.001,0.002) |
| 1992 | 0.763(0.518,1.081) | 0.032(0.022,0.046) |  | 0.016(0.005,0.053) | 0.001(0.000,0.002) |  | 0.082(0.051,0.127) | 0.004(0.002,0.005) |  | 0.035(0.024,0.050) | 0.002(0.001,0.002) |
| 1993 | 0.941(0.638,1.332) | 0.035(0.024,0.049) |  | 0.019(0.006,0.064) | 0.001(0.000,0.002) |  | 0.098(0.061,0.149) | 0.004(0.002,0.006) |  | 0.043(0.029,0.061) | 0.002(0.001,0.002) |
| 1994 | 1.142(0.775,1.617) | 0.038(0.025,0.053) |  | 0.023(0.007,0.077) | 0.001(0.000,0.003) |  | 0.116(0.073,0.172) | 0.004(0.002,0.006) |  | 0.053(0.036,0.074) | 0.002(0.001,0.002) |
| 1995 | 1.336(0.901,1.886) | 0.040(0.027,0.056) |  | 0.027(0.008,0.090) | 0.001(0.000,0.003) |  | 0.135(0.086,0.197) | 0.004(0.003,0.006) |  | 0.064(0.043,0.090) | 0.002(0.001,0.003) |
| 1996 | 1.525(1.034,2.145) | 0.041(0.028,0.058) |  | 0.030(0.009,0.099) | 0.001(0.000,0.003) |  | 0.155(0.100,0.227) | 0.004(0.003,0.006) |  | 0.074(0.050,0.106) | 0.002(0.001,0.003) |
| 1997 | 1.694(1.157,2.370) | 0.042(0.029,0.058) |  | 0.033(0.011,0.109) | 0.001(0.000,0.003) |  | 0.176(0.115,0.258) | 0.004(0.003,0.006) |  | 0.085(0.057,0.120) | 0.002(0.001,0.003) |
| 1998 | 1.840(1.287,2.561) | 0.042(0.029,0.058) |  | 0.036(0.012,0.120) | 0.001(0.000,0.003) |  | 0.199(0.129,0.288) | 0.005(0.003,0.007) |  | 0.094(0.064,0.132) | 0.002(0.002,0.003) |
| 1999 | 1.996(1.415,2.770) | 0.042(0.030,0.059) |  | 0.039(0.013,0.131) | 0.001(0.000,0.003) |  | 0.226(0.149,0.330) | 0.005(0.003,0.007) |  | 0.105(0.072,0.148) | 0.002(0.002,0.003) |
| 2000 | 2.228(1.585,3.086) | 0.043(0.031,0.060) |  | 0.043(0.015,0.142) | 0.001(0.000,0.003) |  | 0.259(0.173,0.372) | 0.005(0.003,0.007) |  | 0.120(0.083,0.167) | 0.002(0.002,0.003) |
| 2001 | 2.518(1.815,3.463) | 0.045(0.033,0.062) |  | 0.046(0.016,0.153) | 0.001(0.000,0.003) |  | 0.292(0.198,0.416) | 0.005(0.004,0.008) |  | 0.137(0.094,0.192) | 0.003(0.002,0.003) |
| 2002 | 2.892(2.093,3.953) | 0.048(0.034,0.065) |  | 0.051(0.018,0.166) | 0.001(0.000,0.003) |  | 0.334(0.232,0.476) | 0.006(0.004,0.008) |  | 0.154(0.106,0.216) | 0.003(0.002,0.004) |
| 2003 | 3.393(2.472,4.593) | 0.051(0.037,0.069) |  | 0.060(0.022,0.184) | 0.001(0.000,0.003) |  | 0.390(0.277,0.544) | 0.006(0.004,0.008) |  | 0.175(0.120,0.244) | 0.003(0.002,0.004) |
| 2004 | 3.967(2.926,5.349) | 0.054(0.040,0.073) |  | 0.068(0.027,0.204) | 0.001(0.000,0.003) |  | 0.458(0.331,0.630) | 0.006(0.005,0.009) |  | 0.199(0.137,0.271) | 0.003(0.002,0.004) |
| 2005 | 4.701(3.471,6.279) | 0.057(0.042,0.077) |  | 0.083(0.035,0.239) | 0.001(0.000,0.003) |  | 0.535(0.390,0.728) | 0.007(0.005,0.009) |  | 0.238(0.165,0.329) | 0.003(0.002,0.004) |
| 2006 | 5.627(4.164,7.493) | 0.061(0.045,0.081) |  | 0.107(0.047,0.283) | 0.001(0.001,0.003) |  | 0.627(0.456,0.836) | 0.007(0.005,0.009) |  | 0.297(0.205,0.414) | 0.003(0.002,0.005) |
| 2007 | 6.834(5.040,9.134) | 0.065(0.048,0.087) |  | 0.139(0.060,0.327) | 0.001(0.001,0.003) |  | 0.747(0.546,0.996) | 0.007(0.005,0.009) |  | 0.383(0.266,0.539) | 0.004(0.003,0.005) |
| 2008 | 7.784(5.756,10.396) | 0.067(0.050,0.090) |  | 0.169(0.068,0.366) | 0.002(0.001,0.003) |  | 0.835(0.616,1.110) | 0.007(0.005,0.010) |  | 0.462(0.318,0.652) | 0.004(0.003,0.006) |
| 2009 | 8.842(6.519,11.833) | 0.070(0.051,0.093) |  | 0.200(0.075,0.411) | 0.002(0.001,0.003) |  | 0.922(0.682,1.211) | 0.007(0.005,0.010) |  | 0.542(0.372,0.761) | 0.004(0.003,0.006) |
| 2010 | 10.173(7.514,13.561) | 0.073(0.054,0.097) |  | 0.236(0.085,0.470) | 0.002(0.001,0.003) |  | 1.034(0.765,1.345) | 0.007(0.006,0.010) |  | 0.631(0.432,0.890) | 0.005(0.003,0.006) |
| 2011 | 11.556(8.416,15.526) | 0.075(0.055,0.101) |  | 0.272(0.093,0.529) | 0.002(0.001,0.003) |  | 1.170(0.858,1.539) | 0.008(0.006,0.010) |  | 0.713(0.487,1.003) | 0.005(0.003,0.007) |
| 2012 | 12.954(9.321,17.394) | 0.078(0.056,0.105) |  | 0.302(0.098,0.578) | 0.002(0.001,0.004) |  | 1.353(0.984,1.795) | 0.008(0.006,0.011) |  | 0.814(0.556,1.154) | 0.005(0.003,0.007) |
| 2013 | 14.501(10.355,19.623) | 0.081(0.058,0.110) |  | 0.332(0.103,0.623) | 0.002(0.001,0.004) |  | 1.539(1.120,2.042) | 0.009(0.006,0.012) |  | 0.931(0.634,1.323) | 0.005(0.004,0.007) |
| 2014 | 16.286(11.604,22.119) | 0.085(0.061,0.115) |  | 0.359(0.109,0.664) | 0.002(0.001,0.004) |  | 1.759(1.285,2.325) | 0.009(0.007,0.012) |  | 1.096(0.749,1.555) | 0.006(0.004,0.008) |
| 2015 | 18.217(13.008,24.826) | 0.089(0.064,0.121) |  | 0.387(0.116,0.709) | 0.002(0.001,0.004) |  | 1.970(1.435,2.625) | 0.010(0.007,0.013) |  | 1.276(0.875,1.799) | 0.006(0.004,0.009) |
| 2016 | 20.354(14.559,27.861) | 0.093(0.067,0.127) |  | 0.420(0.127,0.774) | 0.002(0.001,0.004) |  | 2.180(1.588,2.949) | 0.010(0.007,0.014) |  | 1.469(1.006,2.076) | 0.007(0.005,0.010) |
| 2017 | 22.714(16.202,31.237) | 0.097(0.069,0.134) |  | 0.444(0.138,0.822) | 0.002(0.001,0.004) |  | 2.375(1.716,3.222) | 0.010(0.007,0.014) |  | 1.620(1.101,2.324) | 0.007(0.005,0.010) |
| 2018 | 25.055(17.843,34.415) | 0.100(0.071,0.138) |  | 0.447(0.145,0.829) | 0.002(0.001,0.003) |  | 2.604(1.869,3.534) | 0.010(0.008,0.014) |  | 1.713(1.165,2.459) | 0.007(0.005,0.010) |
| 2019 | 27.210(19.134,37.565) | 0.103(0.072,0.142) |  | 0.442(0.146,0.851) | 0.002(0.001,0.003) |  | 2.814(2.037,3.783) | 0.011(0.008,0.014) |  | 1.740(1.192,2.457) | 0.007(0.005,0.009) |
| 2020 | 27.946(19.563,38.570) | 0.103(0.072,0.142) |  | 0.427(0.143,0.851) | 0.002(0.001,0.003) |  | 2.903(2.118,3.892) | 0.011(0.008,0.014) |  | 1.716(1.194,2.423) | 0.006(0.004,0.009) |
| 2021 | 31.845(22.481,43.887) | 0.108(0.076,0.149) |  | 0.496(0.166,1.006) | 0.002(0.001,0.003) |  | 3.332(2.383,4.478) | 0.011(0.008,0.015) |  | 2.096(1.446,2.981) | 0.007(0.005,0.010) |
| 2022 | 33.638(23.612,46.567) | 0.111(0.078,0.153) |  | 0.546(0.175,1.106) | 0.002(0.001,0.004) |  | 3.571(2.350,5.153) | 0.012(0.008,0.017) |  | 2.432(1.608,3.616) | 0.008(0.005,0.012) |
| 2023 | 33.798(23.704,46.643) | 0.106(0.074,0.146) |  | 0.552(0.177,1.162) | 0.002(0.001,0.004) |  | 3.403(2.231,5.014) | 0.011(0.007,0.016) |  | 2.591(1.663,3.972) | 0.008(0.005,0.012) |

Abbreviations: AUD, alcohol use disorders; ACM, alcoholic cardiomyopathy; ALD, cirrhosis and other chronic liver diseases due to alcohol use; LCA, liver cancer due to alcohol use; VLW, value of lost welfare; GDP, gross domestic product.

## Table 4. Joinpoint regression analysis of VLW from 1990 to 2023 for alcohol-attributable diseases in China

| **Disease** | **Sex** | **Segment** | **Start** | **End** | **AAPC/APC** | **APC 95% LCL** | **APC 95% UCL** | **Test Statistic** | **P-Value** |
| --- | --- | --- | --- | --- | --- | --- | --- | --- | --- |
| AUD | Total | Full Range | 1990 | 2023 | 9.130 | 8.865 | 9.394 | 70.611 | <0.001 |
| ACM | Total | Full Range | 1990 | 2023 | 8.157 | 7.599 | 8.719 | 29.688 | <0.001 |
| ALD | Total | Full Range | 1990 | 2023 | 8.612 | 8.064 | 9.164 | 31.963 | <0.001 |
| LCA | Total | Full Range | 1990 | 2023 | 10.852 | 10.158 | 11.550 | 32.168 | <0.001 |
| AUD | Total | 1 | 1990 | 1995 | 15.123 | 14.467 | 15.782 | 51.997 | <0.001 |
| AUD | Total | 2 | 1995 | 2001 | 6.452 | 5.873 | 7.034 | 24.196 | <0.001 |
| AUD | Total | 3 | 2001 | 2008 | 12.271 | 11.864 | 12.680 | 67.214 | <0.001 |
| AUD | Total | 4 | 2008 | 2017 | 8.555 | 8.297 | 8.813 | 72.841 | <0.001 |
| AUD | Total | 5 | 2017 | 2021 | 5.678 | 4.451 | 6.920 | 9.976 | <0.001 |
| AUD | Total | 6 | 2021 | 2023 | 1.708 | -0.674 | 4.148 | 1.508 | 0.150 |
| ACM | Total | 1 | 1990 | 1995 | 12.223 | 10.263 | 14.217 | 13.812 | <0.001 |
| ACM | Total | 2 | 1995 | 2003 | 5.805 | 4.702 | 6.919 | 11.366 | <0.001 |
| ACM | Total | 3 | 2003 | 2010 | 17.057 | 15.962 | 18.162 | 35.359 | <0.001 |
| ACM | Total | 4 | 2010 | 2016 | 6.059 | 5.081 | 7.047 | 13.392 | <0.001 |
| ACM | Total | 5 | 2016 | 2020 | -1.730 | -3.745 | 0.327 | -1.777 | 0.093 |
| ACM | Total | 6 | 2020 | 2023 | 5.977 | 3.568 | 8.441 | 5.328 | 0.000 |
| ALD | Total | 1 | 1990 | 2003 | 10.284 | 9.989 | 10.579 | 77.121 | <0.001 |
| ALD | Total | 2 | 2003 | 2007 | 11.352 | 9.282 | 13.460 | 12.092 | <0.001 |
| ALD | Total | 3 | 2007 | 2010 | 6.387 | 2.753 | 10.150 | 3.759 | 0.002 |
| ALD | Total | 4 | 2010 | 2015 | 10.154 | 8.939 | 11.382 | 18.399 | <0.001 |
| ALD | Total | 5 | 2015 | 2021 | 5.852 | 4.982 | 6.730 | 14.529 | <0.001 |
| ALD | Total | 6 | 2021 | 2023 | 0.661 | -4.038 | 5.589 | 0.291 | 0.775 |
| LCA | Total | 1 | 1990 | 1996 | 14.631 | 13.610 | 15.662 | 32.192 | <0.001 |
| LCA | Total | 2 | 1996 | 2004 | 8.570 | 7.840 | 9.305 | 25.703 | <0.001 |
| LCA | Total | 3 | 2004 | 2008 | 17.142 | 14.290 | 20.066 | 13.540 | <0.001 |
| LCA | Total | 4 | 2008 | 2017 | 11.110 | 10.494 | 11.729 | 40.001 | <0.001 |
| LCA | Total | 5 | 2017 | 2020 | -0.086 | -5.155 | 5.254 | -0.035 | 0.973 |
| LCA | Total | 6 | 2020 | 2023 | 12.157 | 9.009 | 15.397 | 8.502 | <0.001 |
| AUD | Male | Full Range | 1990 | 2023 | 9.220 | 8.971 | 9.471 | 75.560 | <0.001 |
| ACM | Male | Full Range | 1990 | 2023 | 8.510 | 7.936 | 9.088 | 30.138 | <0.001 |
| ALD | Male | Full Range | 1990 | 2023 | 8.750 | 8.236 | 9.267 | 34.677 | <0.001 |
| LCA | Male | Full Range | 1990 | 2023 | 10.969 | 10.190 | 11.754 | 28.959 | <0.001 |
| AUD | Male | 1 | 1990 | 1995 | 15.138 | 14.502 | 15.778 | 53.699 | <0.001 |
| AUD | Male | 2 | 1995 | 2001 | 6.275 | 5.725 | 6.828 | 24.755 | <0.001 |
| AUD | Male | 3 | 2001 | 2007 | 13.021 | 12.515 | 13.528 | 57.612 | <0.001 |
| AUD | Male | 4 | 2007 | 2017 | 8.763 | 8.564 | 8.962 | 96.921 | <0.001 |
| AUD | Male | 5 | 2017 | 2021 | 6.332 | 5.191 | 7.485 | 12.013 | <0.001 |
| AUD | Male | 6 | 2021 | 2023 | 1.033 | -1.174 | 3.289 | 0.982 | 0.340 |
| ACM | Male | 1 | 1990 | 1995 | 12.876 | 10.885 | 14.902 | 14.362 | <0.001 |
| ACM | Male | 2 | 1995 | 2003 | 5.398 | 4.268 | 6.540 | 10.293 | <0.001 |
| ACM | Male | 3 | 2003 | 2010 | 18.478 | 17.308 | 19.659 | 36.055 | <0.001 |
| ACM | Male | 4 | 2010 | 2016 | 6.540 | 5.523 | 7.566 | 13.940 | <0.001 |
| ACM | Male | 5 | 2016 | 2020 | -1.773 | -3.827 | 0.324 | -1.787 | 0.092 |
| ACM | Male | 6 | 2020 | 2023 | 5.961 | 3.497 | 8.483 | 5.192 | 0.000 |
| ALD | Male | 1 | 1990 | 2003 | 10.518 | 10.230 | 10.806 | 80.989 | <0.001 |
| ALD | Male | 2 | 2003 | 2007 | 11.597 | 9.628 | 13.602 | 13.002 | <0.001 |
| ALD | Male | 3 | 2007 | 2010 | 6.388 | 3.018 | 9.867 | 4.059 | 0.001 |
| ALD | Male | 4 | 2010 | 2015 | 10.200 | 9.072 | 11.339 | 19.920 | <0.001 |
| ALD | Male | 5 | 2015 | 2021 | 5.984 | 5.178 | 6.797 | 16.058 | <0.001 |
| ALD | Male | 6 | 2021 | 2023 | 0.457 | -3.958 | 5.075 | 0.214 | 0.833 |
| LCA | Male | 1 | 1990 | 1996 | 14.170 | 12.994 | 15.358 | 26.998 | <0.001 |
| LCA | Male | 2 | 1996 | 2004 | 9.004 | 8.169 | 9.845 | 23.668 | <0.001 |
| LCA | Male | 3 | 2004 | 2008 | 17.085 | 13.883 | 20.377 | 12.001 | <0.001 |
| LCA | Male | 4 | 2008 | 2017 | 11.110 | 10.420 | 11.804 | 35.696 | <0.001 |
| LCA | Male | 5 | 2017 | 2020 | 1.023 | -4.656 | 7.040 | 0.371 | 0.715 |
| LCA | Male | 6 | 2020 | 2023 | 12.013 | 8.449 | 15.695 | 7.401 | 0.000 |
| AUD | Female | Full Range | 1990 | 2023 | 8.390 | 8.137 | 8.644 | 67.460 | <0.001 |
| ACM | Female | Full Range | 1990 | 2023 | 4.965 | 4.130 | 5.807 | 11.892 | <0.001 |
| ALD | Female | Full Range | 1990 | 2023 | 7.106 | 6.446 | 7.770 | 21.779 | <0.001 |
| LCA | Female | Full Range | 1990 | 2023 | 9.734 | 9.216 | 10.254 | 38.466 | <0.001 |
| AUD | Female | 1 | 1990 | 1995 | 15.049 | 14.506 | 15.594 | 62.555 | <0.001 |
| AUD | Female | 2 | 1995 | 2003 | 7.231 | 6.913 | 7.549 | 49.687 | <0.001 |
| AUD | Female | 3 | 2003 | 2011 | 10.866 | 10.517 | 11.216 | 69.023 | <0.001 |
| AUD | Female | 4 | 2011 | 2016 | 7.565 | 6.730 | 8.405 | 19.759 | <0.001 |
| AUD | Female | 5 | 2016 | 2020 | 1.341 | 0.129 | 2.568 | 2.335 | 0.032 |
| AUD | Female | 6 | 2020 | 2023 | 5.347 | 4.077 | 6.632 | 9.061 | <0.001 |
| ACM | Female | 1 | 1990 | 2008 | 8.319 | 8.073 | 8.566 | 74.208 | <0.001 |
| ACM | Female | 2 | 2008 | 2011 | 1.955 | -3.156 | 7.335 | 0.794 | 0.438 |
| ACM | Female | 3 | 2011 | 2014 | -2.723 | -7.054 | 1.809 | -1.279 | 0.218 |
| ACM | Female | 4 | 2014 | 2017 | 1.128 | -3.026 | 5.460 | 0.564 | 0.580 |
| ACM | Female | 5 | 2017 | 2020 | -2.432 | -6.470 | 1.780 | -1.229 | 0.236 |
| ACM | Female | 6 | 2020 | 2023 | 7.816 | 5.444 | 10.241 | 7.138 | 0.000 |
| ALD | Female | 1 | 1990 | 1997 | 8.428 | 7.431 | 9.433 | 18.495 | <0.001 |
| ALD | Female | 2 | 1997 | 2002 | 7.416 | 5.380 | 9.492 | 7.885 | <0.001 |
| ALD | Female | 3 | 2002 | 2007 | 9.540 | 7.721 | 11.390 | 11.481 | <0.001 |
| ALD | Female | 4 | 2007 | 2010 | 5.281 | 0.445 | 10.350 | 2.309 | 0.034 |
| ALD | Female | 5 | 2010 | 2015 | 8.101 | 6.475 | 9.752 | 10.846 | <0.001 |
| ALD | Female | 6 | 2015 | 2023 | 4.365 | 3.759 | 4.975 | 15.474 | <0.001 |
| LCA | Female | 1 | 1990 | 1996 | 14.334 | 13.502 | 15.172 | 38.697 | <0.001 |
| LCA | Female | 2 | 1996 | 2005 | 7.583 | 7.111 | 8.057 | 35.073 | <0.001 |
| LCA | Female | 3 | 2005 | 2008 | 18.903 | 14.390 | 23.593 | 9.441 | <0.001 |
| LCA | Female | 4 | 2008 | 2016 | 10.467 | 9.910 | 11.028 | 41.524 | <0.001 |
| LCA | Female | 5 | 2016 | 2020 | -1.198 | -3.036 | 0.674 | -1.355 | 0.193 |
| LCA | Female | 6 | 2020 | 2023 | 11.854 | 9.513 | 14.246 | 11.173 | <0.001 |

Note: For the segment labeled "Full Range," the value in the AAPC/APC column represents the AAPC; for all other segments, it represents the APC.

Abbreviations: AUD, alcohol use disorders; ACM, alcoholic cardiomyopathy; ALD, cirrhosis and other chronic liver diseases due to alcohol use; LCA, liver cancer due to alcohol use; VLW, value of lost welfare; AAPC, average annual percentage change; APC, annual percentage change.

## Table 5. Forecasting accuracy of three models for predicting VLW (Billion USD) using a 5-year rolling window approach

| **Disease** | **Gender** | **Model** | **Forecasting accuracy** | | | | | | | | |  | **Rolling forecast performance metrics** | | | | | |
| --- | --- | --- | --- | --- | --- | --- | --- | --- | --- | --- | --- | --- | --- | --- | --- | --- | --- | --- |
|  |  |  | **Validation year** | **Training period** | **Actual value** | | **Predicted value** | | **MAE** | **APE**  **(%)** | **95% Interval Coverage** |  | **MAE** | **MSE** | **RMSE** | **sMAPRE** | **MAPE** | **95% Interval Coverage** |
|  |  |  |  |  | **Value** | **95%UI** | **Value** | **95% UI** |  |  |  |  |  |  |  |  |  |  |
| AUD | Total | ARIMA | 2019 | 1990-2018 | 28.635 | 20.136-39.532 | 28.883 | 28.619-29.147 | 0.248 | 0.867 | Yes |  | 1.486 | 3.886 | 1.971 | 4.71% | 4.70% | 40.00% |
|  |  |  | 2020 | 1990-2019 | 29.106 | 20.375-40.171 | 30.162 | 29.887-30.437 | 1.056 | 3.628 | No |  |  |  |  |  |  |  |
|  |  |  | 2021 | 1990-2020 | 31.845 | 22.481-43.887 | 28.895 | 28.461-29.329 | 2.950 | 9.264 | No |  |  |  |  |  |  |  |
|  |  |  | 2022 | 1990-2021 | 33.120 | 23.249-45.851 | 33.036 | 32.244-33.828 | 0.084 | 0.254 | Yes |  |  |  |  |  |  |  |
|  |  |  | 2023 | 1990-2022 | 32.395 | 22.721-44.707 | 35.484 | 34.700-36.269 | 3.089 | 9.536 | No |  |  |  |  |  |  |  |
|  |  | XGBoost | 2019 | 1990-2018 | 28.635 | 20.136-39.532 | 24.221 | 21.106-25.098 | 4.413 | 15.410 | No |  | 3.507 | 13.693 | 3.700 | 12.10% | 11.33% | 20.00% |
|  |  |  | 2020 | 1990-2019 | 29.106 | 20.375-40.171 | 26.899 | 22.226-29.236 | 2.207 | 7.580 | Yes |  |  |  |  |  |  |  |
|  |  |  | 2021 | 1990-2020 | 31.845 | 22.481-43.887 | 27.063 | 22.409-27.614 | 4.782 | 15.020 | No |  |  |  |  |  |  |  |
|  |  |  | 2022 | 1990-2021 | 33.120 | 23.249-45.851 | 28.947 | 24.487-29.524 | 4.174 | 12.600 | No |  |  |  |  |  |  |  |
|  |  |  | 2023 | 1990-2022 | 32.395 | 22.721-44.707 | 30.438 | 25.618-31.222 | 1.957 | 6.040 | No |  |  |  |  |  |  |  |
|  |  | LightGBM | 2019 | 1990-2018 | 28.635 | 20.136-39.532 | 27.107 | 24.302-29.861 | 1.527 | 5.330 | Yes |  | 1.345 | 2.442 | 1.563 | 4.45% | 4.33% | 100% |
|  |  |  | 2020 | 1990-2019 | 29.106 | 20.375-40.171 | 28.635 | 25.662-31.557 | 0.472 | 1.620 | Yes |  |  |  |  |  |  |  |
|  |  |  | 2021 | 1990-2020 | 31.845 | 22.481-43.887 | 29.106 | 26.057-32.055 | 2.739 | 8.600 | Yes |  |  |  |  |  |  |  |
|  |  |  | 2022 | 1990-2021 | 33.120 | 23.249-45.851 | 31.845 | 28.534-35.135 | 1.276 | 3.850 | Yes |  |  |  |  |  |  |  |
|  |  |  | 2023 | 1990-2022 | 32.395 | 22.721-44.707 | 33.120 | 29.811-36.693 | 0.725 | 2.240 | Yes |  |  |  |  |  |  |  |
|  | Male | ARIMA | 2019 | 1990-2018 | 24.688 | 17.629-33.780 | 24.931 | 24.696-25.165 | 0.242 | 0.981 | No |  | 1.394 | 3.449 | 1.857 | 5.13% | 5.11% | 20.00% |
|  |  |  | 2020 | 1990-2019 | 25.136 | 17.765-34.455 | 26.091 | 25.851-26.330 | 0.955 | 3.799 | No |  |  |  |  |  |  |  |
|  |  |  | 2021 | 1990-2020 | 27.565 | 19.637-37.849 | 24.851 | 24.447-25.255 | 2.714 | 9.847 | No |  |  |  |  |  |  |  |
|  |  |  | 2022 | 1990-2021 | 28.632 | 20.226-39.338 | 28.709 | 28.004-29.413 | 0.077 | 0.268 | Yes |  |  |  |  |  |  |  |
|  |  |  | 2023 | 1990-2022 | 27.757 | 19.549-38.065 | 30.740 | 30.045-31.436 | 2.984 | 10.750 | No |  |  |  |  |  |  |  |
|  |  | XGBoost | 2019 | 1990-2018 | 24.688 | 17.629-33.780 | 20.640 | 17.880-21.281 | 4.049 | 16.400 | No |  | 3.139 | 10.974 | 3.313 | 12.61% | 11.78% | 0.00% |
|  |  |  | 2020 | 1990-2019 | 25.136 | 17.765-34.455 | 22.929 | 18.909-24.939 | 2.207 | 8.780 | No |  |  |  |  |  |  |  |
|  |  |  | 2021 | 1990-2020 | 27.565 | 19.637-37.849 | 23.329 | 19.237-23.681 | 4.237 | 15.370 | No |  |  |  |  |  |  |  |
|  |  |  | 2022 | 1990-2021 | 28.632 | 20.226-39.338 | 25.000 | 20.923-25.290 | 3.631 | 12.680 | No |  |  |  |  |  |  |  |
|  |  |  | 2023 | 1990-2022 | 27.757 | 19.549-38.065 | 26.185 | 21.993-26.869 | 1.571 | 5.660 | No |  |  |  |  |  |  |  |
|  |  | LightGBM | 2019 | 1990-2018 | 24.688 | 17.629-33.780 | 23.215 | 20.808-25.577 | 1.474 | 5.970 | Yes |  | 1.259 | 2.065 | 1.437 | 4.81% | 4.69% | 100.00% |
|  |  |  | 2020 | 1990-2019 | 25.136 | 17.765-34.455 | 24.688 | 22.120-27.214 | 0.448 | 1.780 | Yes |  |  |  |  |  |  |  |
|  |  |  | 2021 | 1990-2020 | 27.565 | 19.637-37.849 | 25.136 | 22.497-27.689 | 2.429 | 8.810 | Yes |  |  |  |  |  |  |  |
|  |  |  | 2022 | 1990-2021 | 28.632 | 20.226-39.338 | 27.565 | 24.693-30.419 | 1.066 | 3.720 | Yes |  |  |  |  |  |  |  |
|  |  |  | 2023 | 1990-2022 | 27.757 | 19.549-38.065 | 28.632 | 25.765-31.726 | 0.875 | 3.150 | Yes |  |  |  |  |  |  |  |
|  | Female | ARIMA | 2019 | 1990-2018 | 3.392 | 2.143-5.048 | 3.372 | 3.330-3.413 | 0.020 | 0.596 | Yes |  | 0.110 | 0.025 | 0.157 | 3.06% | 3.00% | 40.00% |
|  |  |  | 2020 | 1990-2019 | 3.385 | 2.144-5.045 | 3.431 | 3.389-3.472 | 0.045 | 1.336 | No |  |  |  |  |  |  |  |
|  |  |  | 2021 | 1990-2020 | 3.671 | 2.309-5.472 | 3.356 | 3.312-3.400 | 0.316 | 8.595 | No |  |  |  |  |  |  |  |
|  |  |  | 2022 | 1990-2021 | 3.811 | 2.387-5.641 | 3.957 | 3.842-4.073 | 0.146 | 3.832 | No |  |  |  |  |  |  |  |
|  |  |  | 2023 | 1990-2022 | 3.989 | 2.507-5.936 | 3.964 | 3.850-4.079 | 0.025 | 0.619 | Yes |  |  |  |  |  |  |  |
|  |  | XGBoost | 2019 | 1990-2018 | 3.392 | 2.143-5.048 | 2.763 | 2.339-2.833 | 0.629 | 18.540 | No |  | 0.650 | 0.449 | 0.670 | 19.45% | 17.65% | 0.00% |
|  |  |  | 2020 | 1990-2019 | 3.385 | 2.144-5.045 | 3.029 | 2.413-3.338 | 0.356 | 10.520 | No |  |  |  |  |  |  |  |
|  |  |  | 2021 | 1990-2020 | 3.671 | 2.309-5.472 | 2.970 | 2.410-3.251 | 0.701 | 19.090 | No |  |  |  |  |  |  |  |
|  |  |  | 2022 | 1990-2021 | 3.811 | 2.387-5.641 | 3.068 | 2.537-3.080 | 0.743 | 19.510 | No |  |  |  |  |  |  |  |
|  |  |  | 2023 | 1990-2022 | 3.989 | 2.507-5.936 | 3.167 | 2.618-3.215 | 0.822 | 20.610 | No |  |  |  |  |  |  |  |
|  |  | LightGBM | 2019 | 1990-2018 | 3.392 | 2.143-5.048 | 3.348 | 2.984-3.706 | 0.044 | 1.290 | Yes |  | 0.130 | 0.027 | 0.164 | 3.57% | 3.47% | 100.00% |
|  |  |  | 2020 | 1990-2019 | 3.385 | 2.144-5.045 | 3.392 | 3.042-3.736 | 0.007 | 0.190 | Yes |  |  |  |  |  |  |  |
|  |  |  | 2021 | 1990-2020 | 3.671 | 2.309-5.472 | 3.385 | 3.036-3.722 | 0.286 | 7.800 | Yes |  |  |  |  |  |  |  |
|  |  |  | 2022 | 1990-2021 | 3.811 | 2.387-5.641 | 3.671 | 3.296-4.043 | 0.141 | 3.690 | Yes |  |  |  |  |  |  |  |
|  |  |  | 2023 | 1990-2022 | 3.989 | 2.507-5.936 | 3.814 | 3.439-4.216 | 0.175 | 4.390 | Yes |  |  |  |  |  |  |  |
| ACM | Total | ARIMA | 2019 | 1990-2018 | 0.465 | 0.154-0.896 | 0.462 | 0.451-0.473 | 0.004 | 0.765 | Yes |  | 0.032 | 0.002 | 0.041 | 6.48% | 6.35% | 40.00% |
|  |  |  | 2020 | 1990-2019 | 0.445 | 0.149-0.886 | 0.450 | 0.439-0.461 | 0.005 | 1.203 | Yes |  |  |  |  |  |  |  |
|  |  |  | 2021 | 1990-2020 | 0.496 | 0.166-1.006 | 0.423 | 0.412-0.434 | 0.073 | 14.730 | No |  |  |  |  |  |  |  |
|  |  |  | 2022 | 1990-2021 | 0.537 | 0.172-1.089 | 0.577 | 0.551-0.602 | 0.040 | 7.370 | No |  |  |  |  |  |  |  |
|  |  |  | 2023 | 1990-2022 | 0.529 | 0.170-1.114 | 0.569 | 0.543-0.596 | 0.041 | 7.689 | No |  |  |  |  |  |  |  |
|  |  | XGBoost | 2019 | 1990-2018 | 0.465 | 0.154-0.896 | 0.361 | 0.278-0.403 | 0.104 | 22.360 | No |  | 0.110 | 0.013 | 0.113 | 24.87% | 22.03% | 0.00% |
|  |  |  | 2020 | 1990-2019 | 0.445 | 0.149-0.886 | 0.378 | 0.291-0.422 | 0.067 | 14.970 | No |  |  |  |  |  |  |  |
|  |  |  | 2021 | 1990-2020 | 0.496 | 0.166-1.006 | 0.386 | 0.302-0.428 | 0.110 | 22.250 | No |  |  |  |  |  |  |  |
|  |  |  | 2022 | 1990-2021 | 0.537 | 0.172-1.089 | 0.388 | 0.313-0.426 | 0.149 | 27.730 | No |  |  |  |  |  |  |  |
|  |  |  | 2023 | 1990-2022 | 0.529 | 0.170-1.114 | 0.408 | 0.327-0.448 | 0.121 | 22.830 | No |  |  |  |  |  |  |  |
|  |  | LightGBM | 2019 | 1990-2018 | 0.465 | 0.154-0.896 | 0.473 | 0.413-0.512 | 0.007 | 1.580 | Yes |  | 0.027 | 0.001 | 0.031 | 5.50% | 5.36% | 80.00% |
|  |  |  | 2020 | 1990-2019 | 0.445 | 0.149-0.886 | 0.467 | 0.413-0.512 | 0.022 | 4.930 | Yes |  |  |  |  |  |  |  |
|  |  |  | 2021 | 1990-2020 | 0.496 | 0.166-1.006 | 0.455 | 0.408-0.507 | 0.041 | 8.310 | Yes |  |  |  |  |  |  |  |
|  |  |  | 2022 | 1990-2021 | 0.537 | 0.172-1.089 | 0.488 | 0.428-0.528 | 0.050 | 9.210 | No |  |  |  |  |  |  |  |
|  |  |  | 2023 | 1990-2022 | 0.529 | 0.170-1.114 | 0.514 | 0.447-0.556 | 0.015 | 2.730 | Yes |  |  |  |  |  |  |  |
|  | Male | ARIMA | 2019 | 1990-2018 | 0.419 | 0.121-0.823 | 0.417 | 0.407-0.427 | 0.003 | 0.628 | Yes |  | 0.030 | 0.001 | 0.039 | 6.68% | 6.55% | 40.00% |
|  |  |  | 2020 | 1990-2019 | 0.401 | 0.118-0.816 | 0.406 | 0.396-0.415 | 0.005 | 1.333 | Yes |  |  |  |  |  |  |  |
|  |  |  | 2021 | 1990-2020 | 0.449 | 0.131-0.928 | 0.380 | 0.371-0.390 | 0.068 | 15.265 | No |  |  |  |  |  |  |  |
|  |  |  | 2022 | 1990-2021 | 0.484 | 0.143-1.015 | 0.522 | 0.498-0.546 | 0.038 | 7.928 | No |  |  |  |  |  |  |  |
|  |  |  | 2023 | 1990-2022 | 0.475 | 0.139-1.042 | 0.511 | 0.487-0.536 | 0.036 | 7.580 | No |  |  |  |  |  |  |  |
|  |  | XGBoost | 2019 | 1990-2018 | 0.419 | 0.121-0.823 | 0.317 | 0.236-0.358 | 0.102 | 24.400 | No |  | 0.106 | 0.012 | 0.109 | 26.90% | 23.63% | 0.00% |
|  |  |  | 2020 | 1990-2019 | 0.401 | 0.118-0.816 | 0.332 | 0.250-0.373 | 0.069 | 17.100 | No |  |  |  |  |  |  |  |
|  |  |  | 2021 | 1990-2020 | 0.449 | 0.131-0.928 | 0.341 | 0.261-0.381 | 0.108 | 23.970 | No |  |  |  |  |  |  |  |
|  |  |  | 2022 | 1990-2021 | 0.484 | 0.143-1.015 | 0.344 | 0.273-0.380 | 0.140 | 28.860 | No |  |  |  |  |  |  |  |
|  |  |  | 2023 | 1990-2022 | 0.475 | 0.139-1.042 | 0.362 | 0.286-0.400 | 0.113 | 23.810 | No |  |  |  |  |  |  |  |
|  |  | LightGBM | 2019 | 1990-2018 | 0.419 | 0.121-0.823 | 0.430 | 0.379-0.470 | 0.010 | 2.460 | Yes |  | 0.023 | 0.001 | 0.027 | 5.25% | 5.13% | 100.00% |
|  |  |  | 2020 | 1990-2019 | 0.401 | 0.118-0.816 | 0.421 | 0.372-0.462 | 0.020 | 5.040 | Yes |  |  |  |  |  |  |  |
|  |  |  | 2021 | 1990-2020 | 0.449 | 0.131-0.928 | 0.407 | 0.364-0.455 | 0.042 | 9.370 | Yes |  |  |  |  |  |  |  |
|  |  |  | 2022 | 1990-2021 | 0.484 | 0.143-1.015 | 0.448 | 0.400-0.491 | 0.035 | 7.330 | Yes |  |  |  |  |  |  |  |
|  |  |  | 2023 | 1990-2022 | 0.475 | 0.139-1.042 | 0.482 | 0.421-0.520 | 0.007 | 1.450 | Yes |  |  |  |  |  |  |  |
|  | Female | ARIMA | 2019 | 1990-2018 | 0.028 | 0.009-0.065 | 0.028 | 0.028-0.029 | 0.000 | 1.544 | Yes |  | 0.001 | <0.001 | 0.002 | 4.47% | 4.32% | 60.00% |
|  |  |  | 2020 | 1990-2019 | 0.027 | 0.008-0.063 | 0.027 | 0.026-0.028 | 0.000 | 0.146 | Yes |  |  |  |  |  |  |  |
|  |  |  | 2021 | 1990-2020 | 0.03 | 0.009-0.071 | 0.026 | 0.025-0.027 | 0.004 | 12.969 | No |  |  |  |  |  |  |  |
|  |  |  | 2022 | 1990-2021 | 0.033 | 0.009-0.079 | 0.032 | 0.031-0.034 | 0.000 | 1.010 | Yes |  |  |  |  |  |  |  |
|  |  |  | 2023 | 1990-2022 | 0.034 | 0.009-0.082 | 0.036 | 0.034-0.037 | 0.002 | 5.949 | No |  |  |  |  |  |  |  |
|  |  | XGBoost | 2019 | 1990-2018 | 0.028 | 0.009-0.065 | 0.023 | 0.019-0.025 | 0.006 | 19.740 | No |  | 0.007 | <0.001 | 0.007 | 27.25% | 23.81% | 0.00% |
|  |  |  | 2020 | 1990-2019 | 0.027 | 0.008-0.063 | 0.023 | 0.020-0.025 | 0.004 | 16.060 | No |  |  |  |  |  |  |  |
|  |  |  | 2021 | 1990-2020 | 0.03 | 0.009-0.071 | 0.023 | 0.020-0.026 | 0.007 | 23.690 | No |  |  |  |  |  |  |  |
|  |  |  | 2022 | 1990-2021 | 0.033 | 0.009-0.079 | 0.023 | 0.020-0.026 | 0.010 | 29.210 | No |  |  |  |  |  |  |  |
|  |  |  | 2023 | 1990-2022 | 0.034 | 0.009-0.082 | 0.023 | 0.021-0.026 | 0.010 | 30.370 | No |  |  |  |  |  |  |  |
|  |  | LightGBM | 2019 | 1990-2018 | 0.028 | 0.009-0.065 | 0.029 | 0.026-0.032 | 0.001 | 3.330 | Yes |  | 0.002 | <0.001 | 0.002 | 5.79% | 5.63% | 80.00% |
|  |  |  | 2020 | 1990-2019 | 0.027 | 0.008-0.063 | 0.028 | 0.025-0.031 | 0.001 | 3.600 | Yes |  |  |  |  |  |  |  |
|  |  |  | 2021 | 1990-2020 | 0.030 | 0.009-0.071 | 0.027 | 0.024-0.030 | 0.003 | 9.720 | No |  |  |  |  |  |  |  |
|  |  |  | 2022 | 1990-2021 | 0.033 | 0.009-0.079 | 0.030 | 0.027-0.033 | 0.003 | 8.260 | Yes |  |  |  |  |  |  |  |
|  |  |  | 2023 | 1990-2022 | 0.034 | 0.009-0.082 | 0.033 | 0.030-0.036 | 0.001 | 3.020 | Yes |  |  |  |  |  |  |  |
| ALD | Total | ARIMA | 2019 | 1990-2018 | 2.962 | 2.144-3.981 | 2.970 | 2.895-3.046 | 0.009 | 0.293 | Yes |  | 0.143 | 0.036 | 0.189 | 4.33% | 4.27% | 40.00% |
|  |  |  | 2020 | 1990-2019 | 3.023 | 2.206-4.053 | 3.049 | 2.975-3.124 | 0.026 | 0.854 | No |  |  |  |  |  |  |  |
|  |  |  | 2021 | 1990-2020 | 3.332 | 2.383-4.478 | 3.097 | 3.024-3.171 | 0.235 | 7.051 | No |  |  |  |  |  |  |  |
|  |  |  | 2022 | 1990-2021 | 3.516 | 2.314-5.074 | 3.632 | 3.527-3.738 | 0.116 | 3.310 | Yes |  |  |  |  |  |  |  |
|  |  |  | 2023 | 1990-2022 | 3.262 | 2.139-4.806 | 3.592 | 3.478-3.707 | 0.331 | 10.138 | No |  |  |  |  |  |  |  |
|  |  | ARIMA (Sensitivity analysis)* | 2019 | 1990-2018 | 2.962 | 2.144-3.981 | 2.986 | 2.945-3.027 | 0.025 | 0.829 | Yes |  | 0.183 | 0.055 | 0.235 | 5.59% | 5.46% | 20.00% |
|  |  |  | 2020 | 1990-2019 | 3.023 | 2.206-4.053 | 3.106 | 3.064-3.147 | 0.082 | 2.718 | No |  |  |  |  |  |  |  |
|  |  |  | 2021 | 1990-2020 | 3.332 | 2.383-4.478 | 3.085 | 3.035-3.136 | 0.247 | 7.418 | No |  |  |  |  |  |  |  |
|  |  |  | 2022 | 1990-2021 | 3.516 | 2.314-5.074 | 3.641 | 3.540-3.743 | 0.125 | 3.567 | No |  |  |  |  |  |  |  |
|  |  |  | 2023 | 1990-2022 | 3.262 | 2.139-4.806 | 3.700 | 3.590-3.809 | 0.438 | 13.426 | No |  |  |  |  |  |  |  |
|  |  | XGBoost | 2019 | 1990-2018 | 2.962 | 2.144-3.981 | 2.199 | 1.854-2.266 | 0.763 | 25.760 | No |  | 0.644 | 0.448 | 0.669 | 22.42% | 19.99% | 0.00% |
|  |  |  | 2020 | 1990-2019 | 3.023 | 2.206-4.053 | 2.510 | 1.949-2.791 | 0.514 | 16.990 | No |  |  |  |  |  |  |  |
|  |  |  | 2021 | 1990-2020 | 3.332 | 2.383-4.478 | 2.546 | 2.030-2.804 | 0.786 | 23.590 | No |  |  |  |  |  |  |  |
|  |  |  | 2022 | 1990-2021 | 3.516 | 2.314-5.074 | 2.707 | 2.178-2.972 | 0.809 | 23.010 | No |  |  |  |  |  |  |  |
|  |  |  | 2023 | 1990-2022 | 3.262 | 2.139-4.806 | 2.915 | 2.330-3.208 | 0.346 | 10.620 | No |  |  |  |  |  |  |  |
|  |  | LightGBM | 2019 | 1990-2018 | 2.962 | 2.144-3.981 | 2.818 | 2.528-3.102 | 0.144 | 4.860 | Yes |  | 0.191 | 0.044 | 0.209 | 5.93% | 5.84% | 80.00% |
|  |  |  | 2020 | 1990-2019 | 3.023 | 2.206-4.053 | 2.962 | 2.657-3.261 | 0.062 | 2.040 | Yes |  |  |  |  |  |  |  |
|  |  |  | 2021 | 1990-2020 | 3.332 | 2.383-4.478 | 3.023 | 2.710-3.327 | 0.309 | 9.270 | No |  |  |  |  |  |  |  |
|  |  |  | 2022 | 1990-2021 | 3.516 | 2.314-5.074 | 3.332 | 2.989-3.674 | 0.184 | 5.220 | Yes |  |  |  |  |  |  |  |
|  |  |  | 2023 | 1990-2022 | 3.262 | 2.139-4.806 | 3.516 | 3.167-3.893 | 0.254 | 7.800 | Yes |  |  |  |  |  |  |  |
|  | Male | ARIMA | 2019 | 1990-2018 | 2.538 | 1.834-3.433 | 2.563 | 2.525-2.601 | 0.025 | 0.979 | Yes |  | 0.149 | 0.041 | 0.203 | 5.36% | 5.24% | 40.00% |
|  |  |  | 2020 | 1990-2019 | 2.584 | 1.878-3.477 | 2.668 | 2.629-2.706 | 0.084 | 3.248 | No |  |  |  |  |  |  |  |
|  |  |  | 2021 | 1990-2020 | 2.862 | 2.039-3.864 | 2.629 | 2.581-2.678 | 0.233 | 8.134 | No |  |  |  |  |  |  |  |
|  |  |  | 2022 | 1990-2021 | 3.005 | 1.960-4.366 | 3.030 | 2.945-3.115 | 0.024 | 0.815 | Yes |  |  |  |  |  |  |  |
|  |  |  | 2023 | 1990-2022 | 2.783 | 1.800-4.178 | 3.163 | 3.079-3.247 | 0.379 | 13.631 | No |  |  |  |  |  |  |  |
|  |  | XGBoost | 2019 | 1990-2018 | 2.538 | 1.834-3.433 | 1.837 | 1.532-1.889 | 0.701 | 27.620 | No |  | 0.594 | 0.377 | 0.614 | 24.36% | 21.55% | 0.00% |
|  |  |  | 2020 | 1990-2019 | 2.584 | 1.878-3.477 | 2.112 | 1.608-2.364 | 0.472 | 18.270 | No |  |  |  |  |  |  |  |
|  |  |  | 2021 | 1990-2020 | 2.862 | 2.039-3.864 | 2.134 | 1.690-2.355 | 0.729 | 25.450 | No |  |  |  |  |  |  |  |
|  |  |  | 2022 | 1990-2021 | 3.005 | 1.960-4.366 | 2.283 | 1.819-2.514 | 0.723 | 24.050 | No |  |  |  |  |  |  |  |
|  |  |  | 2023 | 1990-2022 | 2.783 | 1.800-4.178 | 2.440 | 1.946-2.687 | 0.343 | 12.330 | No |  |  |  |  |  |  |  |
|  |  | LightGBM | 2019 | 1990-2018 | 2.538 | 1.834-3.433 | 2.409 | 2.161-2.652 | 0.130 | 5.100 | Yes |  | 0.164 | 0.033 | 0.182 | 5.96% | 5.86% | 80.00% |
|  |  |  | 2020 | 1990-2019 | 2.584 | 1.878-3.477 | 2.538 | 2.276-2.796 | 0.046 | 1.760 | Yes |  |  |  |  |  |  |  |
|  |  |  | 2021 | 1990-2020 | 2.862 | 2.039-3.864 | 2.584 | 2.315-2.844 | 0.278 | 9.730 | No |  |  |  |  |  |  |  |
|  |  |  | 2022 | 1990-2021 | 3.005 | 1.960-4.366 | 2.862 | 2.566-3.156 | 0.143 | 4.760 | Yes |  |  |  |  |  |  |  |
|  |  |  | 2023 | 1990-2022 | 2.783 | 1.800-4.178 | 3.005 | 2.706-3.328 | 0.222 | 7.960 | Yes |  |  |  |  |  |  |  |
|  | Female | ARIMA | 2019 | 1990-2018 | 0.243 | 0.168-0.336 | 0.244 | 0.241-0.247 | 0.001 | 0.575 | Yes |  | 0.012 | <0.001 | 0.016 | 4.30% | 4.% | 60.00% |
|  |  |  | 2020 | 1990-2019 | 0.247 | 0.170-0.343 | 0.251 | 0.247-0.254 | 0.003 | 1.270 | Yes |  |  |  |  |  |  |  |
|  |  |  | 2021 | 1990-2020 | 0.27 | 0.180-0.388 | 0.252 | 0.249-0.256 | 0.018 | 6.726 | No |  |  |  |  |  |  |  |
|  |  |  | 2022 | 1990-2021 | 0.29 | 0.186-0.432 | 0.284 | 0.277-0.291 | 0.006 | 2.002 | Yes |  |  |  |  |  |  |  |
|  |  |  | 2023 | 1990-2022 | 0.278 | 0.172-0.422 | 0.308 | 0.301-0.316 | 0.030 | 10.931 | No |  |  |  |  |  |  |  |
|  |  | XGBoost | 2019 | 1990-2018 | 0.243 | 0.168-0.336 | 0.131 | 0.100-0.139 | 0.112 | 46.000 | No |  | 0.113 | 0.013 | 0.114 | 54.16% | 42.56% | 0.00% |
|  |  |  | 2020 | 1990-2019 | 0.247 | 0.170-0.343 | 0.141 | 0.106-0.143 | 0.106 | 42.930 | No |  |  |  |  |  |  |  |
|  |  |  | 2021 | 1990-2020 | 0.27 | 0.180-0.388 | 0.153 | 0.112-0.174 | 0.117 | 43.420 | No |  |  |  |  |  |  |  |
|  |  |  | 2022 | 1990-2021 | 0.29 | 0.186-0.432 | 0.163 | 0.117-0.185 | 0.127 | 43.940 | No |  |  |  |  |  |  |  |
|  |  |  | 2023 | 1990-2022 | 0.278 | 0.172-0.422 | 0.177 | 0.124-0.203 | 0.102 | 36.500 | No |  |  |  |  |  |  |  |
|  |  | LightGBM | 2019 | 1990-2018 | 0.243 | 0.168-0.336 | 0.235 | 0.211-0.257 | 0.008 | 3.300 | Yes |  | 0.013 | <0.001 | 0.015 | 5.06% | 4.94% | 100.00% |
|  |  |  | 2020 | 1990-2019 | 0.247 | 0.170-0.343 | 0.243 | 0.219-0.266 | 0.005 | 1.960 | Yes |  |  |  |  |  |  |  |
|  |  |  | 2021 | 1990-2020 | 0.27 | 0.180-0.388 | 0.247 | 0.223-0.271 | 0.023 | 8.520 | Yes |  |  |  |  |  |  |  |
|  |  |  | 2022 | 1990-2021 | 0.29 | 0.186-0.432 | 0.271 | 0.244-0.297 | 0.019 | 6.680 | Yes |  |  |  |  |  |  |  |
|  |  |  | 2023 | 1990-2022 | 0.278 | 0.172-0.422 | 0.290 | 0.262-0.319 | 0.012 | 4.240 | Yes |  |  |  |  |  |  |  |
| LCA | Total | ARIMA | 2019 | 1990-2018 | 1.831 | 1.255-2.586 | 1.858 | 1.817-1.898 | 0.026 | 1.434 | Yes |  | 0.122 | 0.034 | 0.185 | 5.77% | 5.52% | 60.00% |
|  |  |  | 2020 | 1990-2019 | 1.787 | 1.244-2.524 | 1.780 | 1.745-1.815 | 0.007 | 0.386 | Yes |  |  |  |  |  |  |  |
|  |  |  | 2021 | 1990-2020 | 2.096 | 1.446-2.981 | 1.741 | 1.707-1.776 | 0.354 | 16.909 | No |  |  |  |  |  |  |  |
|  |  |  | 2022 | 1990-2021 | 2.395 | 1.584-3.561 | 2.404 | 2.268-2.541 | 0.009 | 0.395 | Yes |  |  |  |  |  |  |  |
|  |  |  | 2023 | 1990-2022 | 2.483 | 1.594-3.808 | 2.694 | 2.560-2.828 | 0.211 | 8.496 | No |  |  |  |  |  |  |  |
|  |  | XGBoost | 2019 | 1990-2018 | 1.831 | 1.255-2.586 | 1.448 | 1.122-1.486 | 0.383 | 20.920 | No |  | 0.491 | 0.279 | 0.528 | 25.56% | 22.40% | 20.00% |
|  |  |  | 2020 | 1990-2019 | 1.787 | 1.244-2.524 | 1.615 | 1.195-1.825 | 0.172 | 9.620 | Yes |  |  |  |  |  |  |  |
|  |  |  | 2021 | 1990-2020 | 2.096 | 1.446-2.981 | 1.564 | 1.198-1.569 | 0.532 | 25.400 | No |  |  |  |  |  |  |  |
|  |  |  | 2022 | 1990-2021 | 2.395 | 1.584-3.561 | 1.689 | 1.284-1.891 | 0.706 | 29.470 | No |  |  |  |  |  |  |  |
|  |  |  | 2023 | 1990-2022 | 2.483 | 1.594-3.808 | 1.823 | 1.391-1.843 | 0.660 | 26.580 | No |  |  |  |  |  |  |  |
|  |  | LightGBM | 2019 | 1990-2018 | 1.831 | 1.255-2.586 | 1.854 | 1.652-2.051 | 0.022 | 1.210 | Yes |  | 0.153 | 0.039 | 0.198 | 7.30% | 6.90% | 60.00% |
|  |  |  | 2020 | 1990-2019 | 1.787 | 1.244-2.524 | 1.831 | 1.632-2.027 | 0.044 | 2.490 | Yes |  |  |  |  |  |  |  |
|  |  |  | 2021 | 1990-2020 | 2.096 | 1.446-2.981 | 1.787 | 1.586-1.981 | 0.309 | 14.720 | No |  |  |  |  |  |  |  |
|  |  |  | 2022 | 1990-2021 | 2.395 | 1.584-3.561 | 2.096 | 1.874-2.316 | 0.299 | 12.500 | No |  |  |  |  |  |  |  |
|  |  |  | 2023 | 1990-2022 | 2.483 | 1.594-3.808 | 2.395 | 2.151-2.658 | 0.088 | 3.560 | Yes |  |  |  |  |  |  |  |
|  | Male | ARIMA | 2019 | 1990-2018 | 1.325 | 0.897-1.893 | 1.357 | 1.328-1.386 | 0.031 | 2.353 | No |  | 0.146 | 0.034 | 0.183 | 9.04% | 8.92% | 20.00% |
|  |  |  | 2020 | 1990-2019 | 1.293 | 0.884-1.846 | 1.285 | 1.261-1.310 | 0.008 | 0.585 | Yes |  |  |  |  |  |  |  |
|  |  |  | 2021 | 1990-2020 | 1.536 | 1.047-2.219 | 1.250 | 1.225-1.274 | 0.286 | 18.622 | No |  |  |  |  |  |  |  |
|  |  |  | 2022 | 1990-2021 | 1.748 | 1.130-2.668 | 1.986 | 1.886-2.085 | 0.237 | 13.582 | No |  |  |  |  |  |  |  |
|  |  |  | 2023 | 1990-2022 | 1.791 | 1.104-2.838 | 1.961 | 1.856-2.065 | 0.170 | 9.473 | No |  |  |  |  |  |  |  |
|  |  | XGBoost | 2019 | 1990-2018 | 1.325 | 0.897-1.893 | 0.973 | 0.766-1.077 | 0.353 | 26.590 | No |  | 0.422 | 0.196 | 0.443 | 31.23% | 26.82% | 0.00% |
|  |  |  | 2020 | 1990-2019 | 1.293 | 0.884-1.846 | 1.087 | 0.814-1.224 | 0.206 | 15.920 | No |  |  |  |  |  |  |  |
|  |  |  | 2021 | 1990-2020 | 1.536 | 1.047-2.219 | 1.093 | 0.842-1.218 | 0.443 | 28.840 | No |  |  |  |  |  |  |  |
|  |  |  | 2022 | 1990-2021 | 1.748 | 1.130-2.668 | 1.160 | 0.927-1.276 | 0.589 | 33.660 | No |  |  |  |  |  |  |  |
|  |  |  | 2023 | 1990-2022 | 1.791 | 1.104-2.838 | 1.270 | 1.008-1.402 | 0.521 | 29.070 | No |  |  |  |  |  |  |  |
|  |  | LightGBM | 2019 | 1990-2018 | 1.325 | 0.897-1.893 | 1.334 | 1.182-1.483 | 0.009 | 0.640 | Yes |  | 0.108 | 0.021 | 0.146 | 7.12% | 6.70% | 60.00% |
|  |  |  | 2020 | 1990-2019 | 1.293 | 0.884-1.846 | 1.325 | 1.174-1.474 | 0.032 | 2.500 | Yes |  |  |  |  |  |  |  |
|  |  |  | 2021 | 1990-2020 | 1.536 | 1.047-2.219 | 1.293 | 1.140-1.441 | 0.243 | 15.810 | No |  |  |  |  |  |  |  |
|  |  |  | 2022 | 1990-2021 | 1.748 | 1.130-2.668 | 1.536 | 1.373-1.697 | 0.212 | 12.150 | No |  |  |  |  |  |  |  |
|  |  |  | 2023 | 1990-2022 | 1.791 | 1.104-2.838 | 1.748 | 1.569-1.941 | 0.043 | 2.390 | Yes |  |  |  |  |  |  |  |
|  | Female | ARIMA | 2019 | 1990-2018 | 0.415 | 0.287-0.581 | 0.405 | 0.394-0.416 | 0.010 | 2.415 | Yes |  | 0.019 | 0.001 | 0.028 | 4.20% | 4.01% | 80.00% |
|  |  |  | 2020 | 1990-2019 | 0.403 | 0.279-0.558 | 0.397 | 0.386-0.408 | 0.006 | 1.538 | Yes |  |  |  |  |  |  |  |
|  |  |  | 2021 | 1990-2020 | 0.457 | 0.310-0.647 | 0.397 | 0.387-0.407 | 0.060 | 13.171 | No |  |  |  |  |  |  |  |
|  |  |  | 2022 | 1990-2021 | 0.52 | 0.336-0.771 | 0.518 | 0.493-0.543 | 0.002 | 0.435 | Yes |  |  |  |  |  |  |  |
|  |  |  | 2023 | 1990-2022 | 0.569 | 0.350-0.847 | 0.583 | 0.556-0.610 | 0.014 | 2.481 | Yes |  |  |  |  |  |  |  |
|  |  | XGBoost | 2019 | 1990-2018 | 0.415 | 0.287-0.581 | 0.288 | 0.201-0.331 | 0.127 | 30.690 | No |  | 0.151 | 0.025 | 0.157 | 37.26% | 31.24% | 0.00% |
|  |  |  | 2020 | 1990-2019 | 0.403 | 0.279-0.558 | 0.315 | 0.216-0.364 | 0.088 | 21.920 | No |  |  |  |  |  |  |  |
|  |  |  | 2021 | 1990-2020 | 0.457 | 0.310-0.647 | 0.319 | 0.225-0.365 | 0.139 | 30.310 | No |  |  |  |  |  |  |  |
|  |  |  | 2022 | 1990-2021 | 0.52 | 0.336-0.771 | 0.334 | 0.249-0.376 | 0.187 | 35.880 | No |  |  |  |  |  |  |  |
|  |  |  | 2023 | 1990-2022 | 0.569 | 0.350-0.847 | 0.356 | 0.269-0.400 | 0.213 | 37.400 | No |  |  |  |  |  |  |  |
|  |  | LightGBM | 2019 | 1990-2018 | 0.415 | 0.287-0.581 | 0.430 | 0.381-0.478 | 0.015 | 3.480 | Yes |  | 0.038 | 0.002 | 0.044 | 8.19% | 7.83% | 60.00% |
|  |  |  | 2020 | 1990-2019 | 0.403 | 0.279-0.558 | 0.415 | 0.368-0.461 | 0.012 | 3.070 | Yes |  |  |  |  |  |  |  |
|  |  |  | 2021 | 1990-2020 | 0.457 | 0.310-0.647 | 0.403 | 0.357-0.448 | 0.054 | 11.880 | No |  |  |  |  |  |  |  |
|  |  |  | 2022 | 1990-2021 | 0.52 | 0.336-0.771 | 0.457 | 0.410-0.504 | 0.063 | 12.110 | No |  |  |  |  |  |  |  |
|  |  |  | 2023 | 1990-2022 | 0.569 | 0.350-0.847 | 0.520 | 0.468-0.577 | 0.049 | 8.590 | Yes |  |  |  |  |  |  |  |

Abbreviations: AUD, alcohol use disorders; ACM, alcoholic cardiomyopathy; ALD, cirrhosis and other chronic liver diseases due to alcohol use; LCA, liver cancer due to alcohol use; 95% UI, uncertainty interval; 95% UI, 95% uncertainty interval.

## Table 6. Comparative performance of forecasting models across diseases and sexes with Diebold-Mariano test

| **Disease** | **Gender** | **ARIMA vs XGBoost** | |  | **ARIMA vs LightGBM** | |  | **XGBoost vs LightGBM** | |
| --- | --- | --- | --- | --- | --- | --- | --- | --- | --- |
|  |  | **DM Statistic** | ***P*-value** |  | **DM Statistic** | ***P*-value** |  | **DM Statistic** | ***P*-value** |
| AUD | Total | -2.075 | 0.107 |  | 0.718 | 0.513 |  | 3.760 | 0.020 |
|  | Male | -1.863 | 0.136 |  | 0.787 | 0.475 |  | 3.684 | 0.021 |
|  | Female | -4.651 | 0.010 |  | -0.267 | 0.802 |  | 4.865 | 0.008 |
| ACM | Total | -3.976 | 0.017 |  | 0.909 | 0.415 |  | 4.579 | 0.010 |
|  | Male | -4.497 | 0.011 |  | 1.259 | 0.276 |  | 4.920 | 0.008 |
|  | Female | -2.791 | 0.049 |  | 0.000 | 0.999 |  | 2.854 | 0.046 |
| ALD | Total | -3.440 | 0.026 |  | -0.557 | 0.607 |  | 3.785 | 0.019 |
|  | Male | -3.170 | 0.034 |  | 0.370 | 0.730 |  | 4.038 | 0.016 |
|  | Female | -11.067 | <0.001 |  | 0.172 | 0.872 |  | 12.960 | <0.001 |
| LCA | Total | -2.827 | 0.048 |  | -0.210 | 0.844 |  | 3.087 | 0.037 |
|  | Male | -3.595 | 0.023 |  | 2.188 | 0.094 |  | 3.614 | 0.023 |
|  | Female | -3.429 | 0.027 |  | -1.342 | 0.251 |  | 3.601 | 0.023 |

The Diebold-Mariano (DM) test was used to compare predictive accuracy between model pairs using squared error loss. A positive DM statistic indicates that the first model in the comparison has higher forecast error than the second, implying the second model performs better; a negative DM statistic indicates the first model has lower forecast error. Bold p-values (*P* < 0.05) indicate statistically significant differences in predictive accuracy.

Abbreviations: AUD, alcohol use disorders; ACM, alcoholic cardiomyopathy; ALD, cirrhosis and other chronic liver diseases due to alcohol use; LCA, liver cancer due to alcohol use; 95% UI, uncertainty interval.

## Table 7. ARIMA model-based projections of VLW (Billion USD) for alcohol-attributable diseases in China, 2024–2030

| **Disease** | **Gender** | **ARIMA** | **Quality assessment** | | |  | **Ljung-Box** | | **Residual ACF Test** | **Prediction of VLW (95% UI) ($ billion)** | | | | | | |
| --- | --- | --- | --- | --- | --- | --- | --- | --- | --- | --- | --- | --- | --- | --- | --- | --- |
|  |  |  | **AICc** | **AIC** | **BIC** |  | ***Q*** | ***P*** |  | **2024** | **2025** | **2026** | **2027** | **2028** | **2029** | **2030** |
| AUD | Total | ARIMA(0,2,1) | 60.490 | 60.076 | 63.008 |  | 6.757 | 0.662 | No | 33.135(31.987-34.283) | 33.876(31.929-35.822) | 34.616(31.829-37.403) | 35.356(31.666-39.046) | 36.096(31.438-40.754) | 36.836(31.148-42.525) | 37.577(30.797-44.356) |
|  | Male | ARIMA(0,2,1) | 56.818 | 56.045 | 59.336 |  | 6.701 | 0.668 | No | 28.258(27.173-29.343) | 28.759(26.894-30.624) | 29.26(26.563-31.958) | 29.762(26.164-33.359) | 30.263(25.696-34.830) | 30.764(25.162-36.366) | 31.265(24.565-37.966) |
|  | Female | ARIMA(0,2,1) | -88.037 | -88.450 | -85.519 |  | 3.054 | 0.962 | No | 4.156(4.043-4.270) | 4.324(4.113-4.534) | 4.491(4.170-4.812) | 4.658(4.215-5.102) | 4.826(4.248-5.403) | 4.993(4.270-5.716) | 5.16(4.282-6.039) |
| ACM | Total | ARIMA(0,1,1) | -182.461 | -183.289 | -178.799 |  | 4.383 | 0.821 | No | 0.533(0.505-0.560) | 0.547(0.492-0.602) | 0.561(0.489-0.634) | 0.576(0.489-0.662) | 0.59(0.491-0.689) | 0.604(0.495-0.714) | 0.618(0.499-0.738) |
|  | Male | ARIMA(0,1,1) | -187.040 | -187.867 | -183.378 |  | 4.849 | 0.774 | No | 0.480(0.454-0.505) | 0.493(0.442-0.543) | 0.506(0.439-0.573) | 0.519(0.439-0.599) | 0.532(0.440-0.623) | 0.545(0.444-0.646) | 0.558(0.447-0.668) |
|  | Female | ARIMA(0,1,1) | -373.112 | -373.940 | -369.450 |  | 9.142 | 0.330 | No | 0.035(0.033-0.036) | 0.036(0.033-0.039) | 0.036(0.032-0.041) | 0.037(0.032-0.042) | 0.038(0.032-0.044) | 0.039(0.033-0.045) | 0.04(0.033-0.047) |
| ALD | Total | ARIMA(0,1,1) | -57.799 | -57.933 | -56.467 |  | 7.4321 | 0.986 | No | 3.117(2.979-3.255) | 3.201(2.895-3.506) | 3.284(2.875-3.694) | 3.368(2.877-3.860) | 3.452(2.890-4.014) | 3.536(2.912-4.160) | 3.620(2.939-4.301) |
|  | Total (Sensitivity analysis)* | ARIMA(0,2,0) | -74.076 | -74.904 | -70.414 |  | 6.097 | 0.807 | No | 3.007(2.821-3.193) | 2.753(2.337-3.169) | 2.499(1.803-3.194) | 2.244(1.226-3.263) | 1.99(0.611-3.369) | 1.736(-0.038-3.509) | 1.481(-0.719-3.681) |
|  | Male | ARIMA(0,2,1) | -66.977 | -67.390 | -64.459 |  | 5.539 | 0.785 | No | 2.843(2.687-2.999) | 2.903(2.654-3.152) | 2.962(2.621-3.303) | 3.022(2.586-3.458) | 3.082(2.547-3.616) | 3.141(2.503-3.780) | 3.201(2.455-3.947) |
|  | Female | ARIMA(0,2,1) | -233.261 | -233.630 | -230.698 |  | 6.242 | 0.715 | No | 0.287(0.275-0.298) | 0.295(0.277-0.313) | 0.304(0.280-0.328) | 0.312(0.282-0.342) | 0.321(0.284-0.357) | 0.329(0.287-0.372) | 0.338(0.288-0.387) |
| LCA | Total | ARIMA(0,2,0) | -71.202 | -71.335 | -69.869 |  | 10.816 | 0.372 | No | 2.572(2.421-2.722) | 2.66(2.323-2.997) | 2.748(2.184-3.312) | 2.836(2.011-3.662) | 2.925(1.806-4.043) | 3.013(1.575-4.452) | 3.101(1.317-4.886) |
|  | Male | ARIMA(0,2,1) | -89.911 | -90.325 | -87.393 |  | 12.228 | 0.201 | No | 1.889(1.780-1.998) | 1.987(1.816-2.158) | 2.085(1.854-2.316) | 2.183(1.890-2.476) | 2.281(1.925-2.637) | 2.379(1.957-2.801) | 2.477(1.987-2.967) |
|  | Female | ARIMA(0,2,0) | -181.895 | -182.028 | -180.563 |  | 11.429 | 0.325 | No | 0.618(0.591-0.645) | 0.667(0.607-0.727) | 0.716(0.616-0.816) | 0.765(0.618-0.911) | 0.814(0.615-1.012) | 0.862(0.607-1.118) | 0.911(0.595-1.228) |

Abbreviations: AUD, alcohol use disorders; ACM, alcoholic cardiomyopathy; ALD, cirrhosis and other chronic liver diseases due to alcohol use; LCA, liver cancer due to alcohol use; 95% UI, 95% uncertainty interval.

* Sensitivity analysis using ARIMA(0,2,0), the information-criterion-preferred model. The negative lower prediction bounds for 2029-2030 are arithmetic artifacts of the model's extrapolation; VLW cannot be negative by definition.

## Table 8. XGBoost model-based projections of VLW (Billion USD) for alcohol-attributable diseases in China, 2024–2030

| **Disease** | **Gender** | **Optimal parameters** | | | | | | | | |  | **Prediction of VLW (95% UI) ($ billion)** | | | | | | |
| --- | --- | --- | --- | --- | --- | --- | --- | --- | --- | --- | --- | --- | --- | --- | --- | --- | --- | --- |
|  |  | **eta** | **max_depth** | **subsample** | **colsample_bytree** | **min_child_weight** | **gamma** | **lambda** | **alpha** | **nrounds** |  | **2024** | **2025** | **2026** | **2027** | **2028** | **2029** | **2030** |
| AUD | Total | 0.082 | 5 | 0.783 | 0.950 | 1 | 0.178 | 0.870 | 0.599 | 163 |  | 28.604(26.252-31.590) | 28.893(22.258-31.168) | 28.730(21.191-31.687) | 25.441(19.952-32.505) | 25.418(18.032-30.930) | 25.298(16.496-30.242) | 26.353(16.428-31.745) |
|  | Male | 0.082 | 5 | 0.783 | 0.950 | 1 | 0.178 | 0.870 | 0.599 | 163 |  | 24.583(22.480-27.031) | 24.863(19.159-26.833) | 24.760(18.025-27.350) | 21.654(17.283-27.916) | 21.348(15.501-26.352) | 21.277(13.850-25.619) | 22.145(13.377-26.646) |
|  | Female | 0.096 | 6 | 0.715 | 0.950 | 1 | 0.101 | 0.500 | 0.500 | 135 |  | 3.175(2.723-3.330) | 3.383(2.509-3.539) | 3.384(2.405-3.658) | 2.927(2.341-3.742) | 3.028(2.182-3.799) | 2.970(1.831-3.877) | 2.942(1.813-3.712) |
| ACM | Total | 0.048 | 3 | 0.909 | 0.950 | 1 | <0.001 | 3.079 | 0.500 | 264 |  | 0.397(0.337-0.400) | 0.397(0.282-0.430) | 0.391(0.269-0.440) | 0.358(0.247-0.461) | 0.341(0.199-0.479) | 0.331(0.176-0.484) | 0.330(0.177-0.463) |
|  | Male | 0.048 | 3 | 0.909 | 0.950 | 1 | <0.001 | 3.079 | 0.500 | 264 |  | 0.353(0.296-0.382) | 0.353(0.248-0.380) | 0.347(0.237-0.389) | 0.317(0.216-0.410) | 0.302(0.173-0.426) | 0.293(0.151-0.433) | 0.291(0.150-0.415) |
|  | Female | 0.010 | 3 | 0.800 | 0.800 | 3 | 0.100 | 1.000 | 1.000 | 300 |  | 0.024(0.021-0.027) | 0.024(0.019-0.028) | 0.024(0.018-0.029) | 0.024(0.018-0.030) | 0.024(0.017-0.030) | 0.024(0.017-0.031) | 0.024(0.017-0.031) |
| ALD | Total | 0.090 | 6 | 0.600 | 0.950 | 1 | 0.037 | 0.532 | 0.500 | 176 |  | 2.829(2.428-2.942) | 2.954(2.210-3.122) | 2.944(2.110-3.218) | 2.527(2.015-3.290) | 2.590(1.844-3.244) | 2.562(1.578-3.300) | 2.576(1.525-3.176) |
|  | Male | 0.090 | 6 | 0.600 | 0.950 | 1 | 0.037 | 0.532 | 0.500 | 176 |  | 2.380(2.042-2.471) | 2.506(1.853-2.632) | 2.504(1.771-2.713) | 2.146(1.732-2.791) | 2.163(1.569-2.777) | 2.176(1.317-2.838) | 2.175(1.296-2.792) |
|  | Female | 0.036 | 3 | 0.909 | 0.950 | 1 | 0.000 | 2.861 | 0.500 | 351 |  | 0.180(0.130-0.205) | 0.181(0.117-0.188) | 0.179(0.112-0.193) | 0.170(0.106-0.204) | 0.164(0.101-0.206) | 0.162(0.097-0.210) | 0.161(0.096-0.210) |
| LCA | Total | 0.158 | 8 | 0.605 | 0.950 | 1 | 0.011 | 0.530 | 0.500 | 176 |  | 1.962(1.511-2.006) | 2.029(1.295-2.179) | 2.037(1.270-2.240) | 1.761(1.242-2.340) | 1.880(1.142-2.351) | 1.864(0.972-2.467) | 1.942(0.941-2.483) |
|  | Male | 0.070 | 6 | 0.600 | 0.950 | 1 | 0.005 | 1.414 | 0.500 | 189 |  | 1.335(1.098-1.363) | 1.377(0.984-1.423) | 1.374(0.969-1.477) | 1.203(0.934-1.543) | 1.216(0.874-1.520) | 1.207(0.740-1.577) | 1.215(0.741-1.511) |
|  | Female | 0.057 | 4 | 0.830 | 0.950 | 1 | <0.001 | 0.981 | 0.500 | 206 |  | 0.358(0.292-0.365) | 0.359(0.242-0.387) | 0.354(0.230-0.399) | 0.319(0.212-0.422) | 0.303(0.161-0.437) | 0.295(0.145-0.439) | 0.292(0.140-0.427) |

Abbreviations: AUD, alcohol use disorders; ACM, alcoholic cardiomyopathy; ALD, cirrhosis and other chronic liver diseases due to alcohol use; LCA, liver cancer due to alcohol use; 95% UI, 95% uncertainty interval.

## Table 9. LightGBM model-based projections of VLW (Billion USD) for alcohol-attributable diseases in China, 2024–2030

| **Disease** | **Gender** | **Optimal parameters** | | | | | | | | | |  | **Prediction of VLW (95% UI) ($ billion)** | | | | | | |
| --- | --- | --- | --- | --- | --- | --- | --- | --- | --- | --- | --- | --- | --- | --- | --- | --- | --- | --- | --- |
|  |  | **learning_rate** | **num_leaves** | **max_depth** | **feature_fraction** | **bagging_fraction** | **min_data_in_leaf** | **lambda_l1** | **lambda_l2** | **min_gain_to_split** | **nrounds** |  | **2024** | **2025** | **2026** | **2027** | **2028** | **2029** | **2030** |
| AUD | Total | 0.418 | 18 | 3 | 0.733 | 0.901 | 2 | <0.001 | <0.001 | <0.001 | 198 |  | 31.768(28.338-35.220) | 31.840(27.663-34.544) | 31.671(28.189-35.071) | 32.375(28.486-35.367) | 32.109(28.980-35.862) | 31.832(19.039-44.499) | 31.781(16.120-47.384) |
|  | Male | 0.485 | 7 | 4 | 1 | 0.744 | 2 | <0.001 | 0.082 | <0.001 | 195 |  | 27.530(23.805-29.766) | 26.422(23.225-29.186) | 27.094(24.177-30.138) | 27.457(24.046-30.007) | 27.601(24.532-30.493) | 27.109(24.674-30.635) | 27.182(22.462-30.448) |
|  | Female | 0.398 | 13 | 6 | 0.756 | 0.700 | 2 | <0.001 | <0.001 | <0.001 | 53 |  | 3.922(3.417-4.233) | 3.999(3.511-4.327) | 3.706(3.385-4.294) | 3.807(3.362-4.180) | 3.925(3.497-4.316) | 3.907(3.528-4.346) | 3.889(3.426-4.403) |
| ACM | Total | 0.500 | 20 | 6 | 0.981 | 0.700 | 4 | 0.070 | 0.100 | <0.001 | 200 |  | 0.494(0.437-0.547) | 0.496(0.422-0.531) | 0.490(0.430-0.540) | 0.500(0.445-0.554) | 0.505(0.452-0.561) | 0.500(0.446-0.555) | 0.499(0.422-0.564) |
|  | Male | 0.300 | 16 | 4 | 0.700 | 0.7 | 5 | <0.001 | 2.562 | <0.001 | 50 |  | 0.441(0.394-0.493) | 0.443(0.384-0.483) | 0.444(0.389-0.488) | 0.454(0.401-0.500) | 0.466(0.418-0.517) | 0.462(0.412-0.511) | 0.462(0.412-0.511) |
|  | Female | 0.500 | 16 | 6 | 0.753 | 0.704 | 2 | <0.001 | 0.099 | <0.001 | 50 |  | 0.033(0.030-0.036) | 0.033(0.029-0.035) | 0.033(0.030-0.036) | 0.033(0.026-0.041) | 0.033(0.024-0.042) | 0.033(0.022-0.043) | 0.032(0.021-0.044) |
| ALD | Total | 0.300 | 15 | 3 | 1 | 0.776 | 2 | <0.001 | 0.039 | <0.001 | 190 |  | 3.253(2.891-3.617) | 3.180(2.824-3.550) | 3.204(2.466-3.952) | 3.202(2.276-4.133) | 3.193(2.114-4.273) | 3.210(1.874-4.564) | 3.180(1.610-4.731) |
|  | Male | 0.464 | 14 | 5 | 1 | 0.834 | 3 | <0.001 | 0.007 | <0.001 | 200 |  | 2.771(2.445-3.067) | 2.715(2.386-3.008) | 2.711(2.090-3.336) | 2.703(1.920-3.476) | 2.755(2.459-3.081) | 2.794(2.450-3.214) | 2.800(2.405-3.127) |
|  | Female | 0.415 | 16 | 3 | 1 | 1 | 2 | <0.001 | 0.081 | <0.001 | 186 |  | 0.280(0.247-0.304) | 0.264(0.241-0.298) | 0.269(0.213-0.326) | 0.276(0.245-0.302) | 0.279(0.251-0.308) | 0.271(0.250-0.307) | 0.276(0.245-0.302) |
| LCA | Total | 0.471 | 5 | 3 | 1 | 0.893 | 2 | <0.001 | 0.093 | <0.001 | 50 |  | 2.439(2.110-2.637) | 2.413(2.136-2.663) | 2.423(1.895-2.955) | 2.438(1.762-3.105) | 2.449(1.613-3.290) | 2.448(1.509-3.407) | 2.418(1.288-3.550) |
|  | Male | 0.500 | 10 | 6 | 0.700 | 0.799 | 2 | <0.001 | 0.079 | <0.001 | 50 |  | 1.791(1.531-1.911) | 1.736(1.573-1.953) | 1.735(1.351-2.121) | 1.774(1.582-1.963) | 1.786(1.193-2.387) | 1.785(1.058-2.528) | 1.763(1.533-2.025) |
|  | Female | 0.457 | 5 | 6 | 1 | 0.791 | 2 | <0.001 | <0.001 | <0.001 | 178 |  | 0.518(0.459-0.578) | 0.520(0.459-0.578) | 0.534(0.469-0.588) | 0.536(0.479-0.598) | 0.534(0.480-0.599) | 0.517(0.465-0.596) | 0.512(0.452-0.594) |

Abbreviations: AUD, alcohol use disorders; ACM, alcoholic cardiomyopathy; ALD, cirrhosis and other chronic liver diseases due to alcohol use; LCA, liver cancer due to alcohol use; 95% UI, 95% uncertainty interval.
